# Annotated type catalogue of the Megaspiridae, Orthalicidae, and Simpulopsidae (Mollusca, Gastropoda, Orthalicoidea) in the Natural History Museum, London

**DOI:** 10.3897/zookeys.470.8548

**Published:** 2015-01-12

**Authors:** Abraham S. H. Breure, Jonathan D. Ablett

**Affiliations:** 1 Naturalis Biodiversity Center, P.O. Box 9517, Leiden, the Netherlands; 2 Natural History Museum, Division of Higher Invertebrates, London, SW7 5BD, UK

**Keywords:** Megaspiridae, Orthalicidae, Simpulopsidae, types

## Abstract

The type status is described for 65 taxa of the Orthalicoidea, classified within the families Megaspiridae (14), Orthalicidae (30), and Simpulopsidae (20); one taxon is considered a nomen inquirendum. Lectotypes are designated for the following taxa: *Helix brephoides* d’Orbigny, 1835; *Simpulopsis cumingi* Pfeiffer, 1861; Bulimulus (Protoglyptus) dejectus Fulton, 1907; *Bulimus iris* Pfeiffer, 1853. The type status of *Bulimus salteri* Sowerby III, 1890, and Strophocheilus (Eurytus) subirroratus da Costa, 1898 is now changed to lectotype according Art. 74.6 ICZN. The taxa *Bulimus loxostomus* Pfeiffer, 1853, *Bulimus marmatensis* Pfeiffer, 1855, *Bulimus meobambensis* Pfeiffer, 1855, and Orthalicus powissianus var. niveus Preston 1909 are now figured for the first time. The following taxa are now considered junior subjective synonyms: *Bulimus marmatensis* Pfeiffer, 1855 = Helix (Cochlogena) citrinovitrea Moricand, 1836; *Vermiculatus* Breure, 1978 = *Bocourtia* Rochebrune, 1882. New combinations are: Kuschelenia (Bocourtia) Rochebrune, 1882; Kuschelenia (Bocourtia) aequatoria (Pfeiffer, 1853); Kuschelenia (Bocourtia) anthisanensis (Pfeiffer, 1853); Kuschelenia (Bocourtia) aquila (Reeve, 1848); Kuschelenia (Bocourtia) badia (Sowerby I, 1835); Kuschelenia (Bocourtia) bicolor (Sowerby I, 1835); Kuschelenia (Bocourtia) caliginosa (Reeve, 1849); Kuschelenia (Bocourtia) coagulata (Reeve, 1849); Kuschelenia (Bocourtia) cotopaxiensis (Pfeiffer, 1853); Kuschelenia (Bocourtia) filaris (Pfeiffer, 1853); *Kara indentata* (da Costa, 1901); *Clathrorthalicus magnificus* (Pfeiffer, 1848); Simpulopsis (Eudioptus) marmartensis (Pfeiffer, 1855); Kuschelenia (Bocourtia) nucina (Reeve, 1850); Kuschelenia (Bocourtia) ochracea (Morelet, 1863); Kuschelenia (Bocourtia) peaki (Breure, 1978); Kuschelenia (Bocourtia) petiti (Pfeiffer, 1846); *Clathrorthalicus phoebus* (Pfeiffer, 1863); Kuschelenia (Bocourtia) polymorpha (d’Orbigny, 1835); *Scholvienia porphyria* (Pfeiffer, 1847); Kuschelenia (Bocourtia) purpurata (Reeve, 1849); Kuschelenia (Bocourtia) quechuarum Crawford, 1939; *Quechua salteri* (Sowerby III, 1890); Kuschelenia (Bocourtia) subfasciata Pfeiffer, 1853; *Clathrorthalicus victor* (Pfeiffer, 1854). In an addedum a lectotype is being designated for Bulimulus (Drymaeus) interruptus var. pallidus Preston, 1909. An index is included to all taxa mentioned in this paper and the preceding ones in this series (Breure and Ablett 2011, 2012, 2014).

## Introduction

This is the fourth paper on the types of Orthalicoidea in the Natural History Museum, London. Earlier papers (Breure and Ablett 2011, 2012, 2014) have presented the context of the collection, the criteria used for the selection of lectotypes, some biohistorical notes, and a list of type specimens belonging to the Amphibulimidae, Bothriembryontidae, Bulimulidae, and Odontostomidae. The aim of this paper is to provide data on the type specimens classified within the Megaspiridae, Orthalicidae, and Simpulopsidae (sensu Breure and Romero 2012). The paper is concluded with an addenda and corrigenda to the whole series of papers, including a list of taxa of which no type material could be found; in the Appendix, an index to all taxa treated in the four papers is given.

References are given to the original publication, plus those of following papers where type material has been mentioned or is (re-)figured. Dates of publication are in accordance with Coan et al. (2013a, 2013b) and Duncan (1937). Abbreviations used for depositories of material are: ANSP, Academy of Natural Sciences, Philadelphia, U.S.A.; MHNG, Muséum d’Histoire naturelle, Genève, Switzerland; MNHN, Muséum nationale d’Histoire naturelle, Paris, France; MZSP, Museu de Zoología, São Paulo, Brazil; NHMUK, Natural History Museum, London, U.K.; RBINS, Royal Belgian Institute of Natural Sciences, Brussels, Belgium. Other abbreviations used are: / end of line in cited text; coll., collection; D, diameter; H, shell height; ICZN, the International Commission on Zoological Nomenclature; leg., *legit*, collected by; W, number of whorls; +, used for specimens with a broken apex. See Breure and Whisson (2012: fig. 1) for the way measurements on the shell have been taken. Label styles in the Cuming collection (“M.C. label style”) are explained in Breure and Ablett (2011: 7–8). Although most figures have been composed with the shells enlarged, their relative size is approximately maintained; the actual shell height is given in the figures legends.

## Systematics

### Systematic list of taxa arranged in generic order

This systematic list follows Breure (1979) as far as appropriate. The generic classification has been adapted from Breure (1979), Breure and Schouten (1985), and unpublished data from the senior author; the family classification is amended as proposed by Breure and Romero (2012). It may be noted that ongoing phylogenetic research may alter the classification. Within the family, genus and species level taxa are presented in alphabetical order.

#### Family Megaspiridae Pilsbry, 1904

##### Megaspira

Taxon classificationAnimaliaStylommatophoraMegaspiridae

Jay, 1836

elata Gould, 1847.

##### Thaumastus

Taxon classificationAnimaliaStylommatophoraMegaspiridae

Albers, 1860

achilles Pfeiffer, 1853; *ascendens* Pfeiffer, 1853; *buckleyi* Higgins, 1872; *consimilis* Reeve, 1848; *foveolatus* Reeve, 1849; *hartwegi* Pfeiffer in Philippi, 1846; *inca* d’Orbigny, 1835; *insolitus* Preston, 1909; *integer* Pfeiffer, 1855; *loxostomus* Pfeiffer 1853; *magnificus* Grateloup, 1839; *plumbeus* Pfeiffer, 1855; *requieni* Pfeiffer, 1853.

###### Remarks.

Molecular studies (Breure and Romero 2012, Breure unpublished data) strongly suggest that this genus, treated with several subgenera by Breure (1979), is polyphyletic and only the nominate taxon is grouping with *Megaspira*. However, further studies are needed as taxon sampling has been relatively low until now.

#### Family Orthalicidae Martens in Albers, 1860

##### Clathrorthalicus

Taxon classificationAnimaliaStylommatophoraOrthalicidae

Strebel, 1909

magnifica Pfeiffer, 1848; *phoebus* Pfeiffer, 1863; *victor* Pfeiffer, 1854.

##### Corona

Taxon classificationAnimaliaStylommatophoraOrthalicidae

Albers, 1850

gracilis E.A. Smith, 1902.

##### Kara

Taxon classificationAnimaliaStylommatophoraOrthalicidae

Strebel, 1910

indentatus da Costa, 1901; *thompsonii* Pfeiffer, 1845; *yanamensis* Morelet, 1863.

##### Liguus

Taxon classificationAnimaliaStylommatophoraOrthalicidae

Montfort, 1810

murrea Reeve, 1849.

##### Orthalicus

Taxon classificationAnimaliaStylommatophoraOrthalicidae

Beck, 1837

bensoni Reeve, 1849; *bifulguratus* Reeve, 1849; *boucardi* Pfeiffer, 1860; *mars* Pfeiffer, 1861; *phlogera* d’Orbigny, 1835.

##### Porphyrobaphe (Oxyorthalicus)

Taxon classificationAnimaliaStylommatophoraOrthalicidae

Strebel, 1909

subirroratus da Costa, 1898.

##### Porphyrobaphe (Porphyrobaphe)

Taxon classificationAnimaliaStylommatophoraOrthalicidae

Shuttleworth, 1856

approximata Fulton, 1896; *iris* Pfeiffer, 1853; *irroratus* Reeve, 1849; *saturanus* Pfeiffer, 1860.

##### Quechua

Taxon classificationAnimaliaStylommatophoraOrthalicidae

Strebel, 1910

salteri Sowerby III, 1890.

###### Remarks.

Both this taxon described by Strebel and the next one (*Scholvienia* Strebel, 1910), previously treated as subgenera of *Thaumastus* (Breure 1979, Schileyko 1999), are now elevated to generic rank as several species appear as distinct groups in molecular studies (Breure unpublished data). This genus is only provisionally ranked with this family, and further molecular research with increased taxon sampling is needed to give better insight into the systematic position of this group.

##### Scholvienia

Taxon classificationAnimaliaStylommatophoraOrthalicidae

Strebel, 1910

alutaceus Reeve, 1849; *brephoides* d’Orbigny, 1835; *porphyrius* Pfeiffer, 1847.

##### Sultana (Metorthalicus)

Taxon classificationAnimaliaStylommatophoraOrthalicidae

Pilsbry, 1899

deburghiae Reeve, 1859; *fraseri* Pfeiffer, 1858; *gloriosus* Pfeiffer, 1862; *kelletti* Reeve, 1850; *niveus* Preston, 1909; *vicaria* Fulton, 1896; *yatesi* Pfeiffer, 1855.

##### Sultana (Sultana)

Taxon classificationAnimaliaStylommatophoraOrthalicidae

Shuttleworth, 1856

meobambensis Pfeiffer, 1855.

#### Family Simpulopsidae Schileyko, 1999

##### Leiostracus

Taxon classificationAnimaliaStylommatophoraSimpulopsidae

Albers, 1850

clouei Pfeiffer, 1857; *demerarensis* Pfeiffer, 1861; *jeffreysi* Pfeiffer, 1852; *obliquus* Reeve, 1849; *opalinus* Sowerby I, 1833; *sarcochilus* Pfeiffer, 1857; *subtuszonata* Pilsbry, 1899.

##### Rhinus

Taxon classificationAnimaliaStylommatophoraSimpulopsidae

Albers, 1860

hyaloideus Pfeiffer, 1855; *ovulum* Reeve, 1849.

##### Simpulopsis (Eudioptus)

Taxon classificationAnimaliaStylommatophoraSimpulopsidae

Albers, 1860

ephippium Ancey, 1904; *marmatensis* Pfeiffer, 1855.

##### Simpulopsis (Simpulopsis)

Taxon classificationAnimaliaStylommatophoraSimpulopsidae

Beck, 1837

aenea Pfeiffer, 1861; *corrugatus* Guppy, 1866; *cumingi* Pfeiffer 1861; *decussata* Pfeiffer, 1857; *gomesae* da Silva & Thomé, 2006; *miersi* Pfeiffer, 1857; *salomonia* Pfeiffer, 1953; *simulus* Morelet, 1851; *vincentina* E.A. Smith, 1895.

##### Nomen inquirendum

*dejectus* Fulton, 1907.

### Alphabetic list of taxa by species name

#### Bulimus achilles

Taxon classificationAnimaliaStylommatophoraBulimulidae

Pfeiffer, 1853

Figs 1i–ii , L1i

Bulimus achilles Pfeiffer 1853b: 378; Pfeiffer 1854b: 137; Pfeiffer 1855 in Küster and Pfeiffer 1840–1865: 247, pl. 66 fig. 9; Breure 1979: 44.
Thaumastus (Thaumastus) taunaisii achilles ; Breure 1978: 32 (lectotype designation).

##### Type locality.

[Brazil] “in ripis fluvii Amazonum”.

##### Label.

“Banks of Amazon”, taxon label in Pfeiffer’s handwriting. M.C. label style IV.

##### Dimensions.

“Long. 57, diam. 25 mill.”. Figured specimen H 58.0, D 25.5, W 6.4.

##### Type material.

NHMUK 1975268, lectotype, 1975269, 2 paralectotypes (Cuming coll.).

##### Remarks.

Pfeiffer did not state on how many specimens his description was based, but the species was described from the Cuming collection. The original label also mentions “between B. Largilliertii / + taunasii”; a label in a second (later?) hand has added “Prov. S. Paulo, Brazil / (Nehring)”.The current systematic position is according to Richardson (1995: 384).

##### Current systematic position.

Megaspiridae, *Thaumastus taunaisii* (Férussac, 1822).

#### Simpulopsis aenea

Taxon classificationAnimaliaStylommatophoraOrthalicidae

Pfeiffer, 1861

Figs 27i–iii , L1ii

Simpulopsis aenea Pfeiffer 1861a [April]: 84; Pfeiffer 1861b [May]: 27; Reeve 1862 [1860–1862]: pl. 1 fig. 7; Pfeiffer 1868a: 22; Breure 1979: 134.

##### Type locality.

“Parada, reipublicae mexicanae (*Sallé*)”.

##### Label.

“Parada, Mexico, M^r^ Sallé”, taxon label in Pfeiffer’s handwriting. M.C. label style III.

##### Dimensions.

“Diam. maj. 9, min. 8, alt. 5 1/2 mill.”. Figured specimen H 8.98, D 10.6, W 2.5.

##### Type material.

NHMUK 20140830, three syntypes, Sallé leg. (Cuming coll.).

##### Remarks.

Pfeiffer did not state on how many specimens his description was based. The current systematic position follows Thompson (2011: 130). The reference to this species in Richardson (1995: 361) cites the wrong author; the first two citations in his list should be omitted.

##### Current systematic position.

Simpulopsidae, Simpulopsis (Simpulopsis) aenea Pfeiffer, 1861.

#### Bulimus alutaceus

Taxon classificationAnimaliaStylommatophoraBulimulidae

Reeve, 1849

Figs 10i–iv , L1iii

Bulimus alutaceus Reeve 1849 [1848–1850]: pl. 72 fig. 522; Reeve 1850b: 99; Pfeiffer 1853b: 324; Breure 1979: 40 [cited with the wrong year].
Strophocheilus alutaceus ; Pilsbry 1895 [1895–1896]: 59, pl. 23 fig. 61.
Thaumastus (Scholvienia) alutaceus ; Breure 1978: 40, fig. 47 (lectotype designation).

##### Type locality.

“Cuzco, Bolivia; W. Lobb”.

##### Label.

“Cuzco”. M.C. label style III, V.

##### Dimensions.

Not given. Figured specimen H 35.5, D 16.5, W 6.6.

##### Type material.

NHMUK 1975148, lectotype, 1975149, one paralectotype. W. Lobb leg. (Cuming coll.).

##### Remarks.

Reeve did not state on how many specimens his description was based. As Weyrauch (1964: 46) has argued, the type locality is probably in error. The current systematic position follows Richardson (1995: 371) at the species level. The familiar arrangement cannot be ascertained at present; tentatively this taxon is classified with the Orthalicidae until further research has proven its relationships.

##### Current systematic position.

?Orthalicidae, *Scholvienia alutacea* (Reeve, 1849).

#### Porphyrobaphe approximata

Taxon classificationAnimaliaStylommatophoraOrthalicidae

Fulton, 1896

Figs 14i–ii , L1iv

Porphyrobaphe approximata Fulton 1896: 103; Fulton 1897: pl. 6 fig. 6; Pilsbry 1899: 208, pl. 40 fig. 1; Linares and Vera 2012: 156 [incorrect original name].

##### Type locality.

[Colombia] “Bogota”.

##### Label.

“Bogota”, in Fulton’s handwriting.

##### Dimensions.

“Long. 67 millim., maj. diam. 31 millim.”. Figured specimen H 65.7, D 32.5, W 6.5.

##### Type material.

NHMUK 1895.12.19.44, one syntype (ex Fulton).

##### Remarks.

Fulton did not state on how many specimens his description was based; the single specimen found corresponds to his figure and is herein considered as syntype. The current systematic position is according to Richardson (1993: 117).

##### Current systematic position.

Orthalicidae, Porphyrobaphe (Porphyrobaphe) approximata Fulton, 1896.

#### Bulimus ascendens

Taxon classificationAnimaliaStylommatophoraBulimulidae

Pfeiffer, 1853

Figs 2v–vi , L2i

Bulimus ascendens Pfeiffer 1853b: 378; Pfeiffer 1854b: 136; Pfeiffer 1855 in Küster and Pfeiffer 1840–1865: 247, pl. 66 fig. 7; Breure 1979: 44.
Thaumastus (Thaumastus) ascendens ; Breure 1978: 26 (lectotype designation).
Thaumastus ascendens ; Simone 2006: 152, fig. 515.

##### Type locality.

“Brasilia”.

##### Label.

“Brazils”, taxon label in Pfeiffer’s handwriting. M.C. label style IV.

##### Dimensions.

“Long. 95 mill., diam. 34 mill.”. Figured specimen H 92.0, D 39.0, W –.

##### Type material.

NHMUK 1975274, lectotype (Cuming coll.).

##### Remarks.

Pfeiffer did not state on how many specimens his description was based, but the species was described from the Cuming collection. The top whorls of the specimen are missing. The current systematic position is according to Simone (2006).

##### Current systematic position.

Megaspiridae, *Thaumastus ascendens* (Pfeiffer, 1853).

#### Bulimus bensoni

Taxon classificationAnimaliaStylommatophoraBulimulidae

Reeve, 1849

Figs 11v–vii , L2ii

Bulimus bensoni Reeve 1849 [1848–1850]: pl. 78 fig. 571; Pfeiffer 1853 in Küster and Pfeiffer 1840–1865: 75, pl. 21 fig. 1.
Oxystyla bensoni ; Pilsbry 1899: 147, pl. 31 fig. 64.

##### Type locality.

“Banks of the river Amazon”.

##### Label.

“Brazil”. M.C. label style I, V.

##### Dimensions.

Not given. Figured specimen H 66.6, D 35.0, W 7.9.

##### Type material.

NHMUK 1975582, three possible syntypes (Cuming coll.).

##### Remarks.

Reeve described this taxon from “Mus. Benson”, but did not state on how many specimens his description was based. According to Tillier (1980: 73), the figured specimen is in the collection of the Museum of Zoology, University of Cambridge. The material found is therefore considered as possible syntypes. The specimen figured by Pfeiffer 1853 (in Küster and Pfeiffer 1840–1865: pl. 21 fig. 1) was smaller than Reeve’s figure, but also originated from Benson’s collection.

##### Current systematic position.

Orthalicidae, *Orthalicus bensoni* (Reeve, 1849).

#### Bulimus bifulguratus

Taxon classificationAnimaliaStylommatophoraBulimulidae

Reeve, 1849

Figs 12i–ii , L2iii

Bulimus bifulguratus Reeve 1849 [1848–1850]: pl. 82 fig. 606.
Oxystyla bifulgurata ; Pilsbry 1899: 143, pl. 31 figs 59–60.
Orthalicus bifulguratus ; Breure and Schouten 1985: 29 (lectotype designation); Linares and Vera 2012: 151.

##### Type locality.

[Colombia] “Andes of Columbia”.

##### Label.

“Andes of Colombia”. M.C. label style I, V.

##### Dimensions.

Not given. Figured specimen H 56.9, D 32.8, W 5.8.

##### Type material.

NHMUK 20140082, lectotype (Cuming coll.).

##### Remarks.

Reeve did not state on how many specimens his description was based. The lectotype is not full-grown as shown by the lip. Pfeiffer (1853b: 388) mentioned specimens from both “Mus. Cuming, Benson. et Coll. Nr. 260 jun.”; as shell height he gave 65 mm, which was likely measured on a full-grown specimen. Richardson (1993: 98, 110) treated this taxon both as a separate species and as a junior subjective synonym of *Bulinus princeps* Broderip in Sowerby I 1833; tentatively this taxon is retained as a full species awaiting further studies.

##### Current systematic position.

Orthalicidae, *Orthalicus bifulguratus* (Reeve, 1849).

#### Orthalicus boucardi

Taxon classificationAnimaliaStylommatophoraOrthalicidae

Pfeiffer, 1860

Figs 12iii–v , L3i

Orthalicus boucardi Pfeiffer 1860: 138, pl. 51 fig. 7.

##### Type locality.

“Mexico (*Mr. Boucard*)”.

##### Label.

“Betaza Mexico / Mr. Boucard”, taxon label in Pfeiffer’s handwriting. M.C. label style IV.

##### Dimensions.

“Long. 43, diam. 25–26 mill.”. Figured specimen H 54.8, D 29.7, W 6.9.

##### Type material.

NHMUK 20140081, three syntypes, Boucard leg. (Cuming coll.).

##### Remarks.

Pfeiffer did not state on how many specimens his description was based. The specimens found are larger than the measurements given by Pfeiffer, but undoubtedly were collected by Mr. Boucard, bear Pfeiffer’s handwriting, and are considered syntypes herein. The type locality (Oaxaca, Sierra de Betaza) was specified by Martens 1893 [1890–1901]: 101 on the basis of Boucard’s material. The current systematic position follows Thompson (2011: 101).

##### Current systematic position.

Orthalicidae, *Orthalicus boucardi* (Pfeiffer, 1860).

#### Helix brephoides

Taxon classificationAnimaliaStylommatophoraHelicidae

d’Orbigny, 1835

Figs 10v–vii , L3ii

Helix brephoides d’Orbigny 1835: 17.
Bulimus brephoides ; d’Orbigny 1837 [1834–1847]: 294, pl. 38 figs 8–9 [text 6 May 1838]; Gray 1854: 19.
Strophocheilus brephoides ; Pilsbry 1895 [1895–1896]: 57, pl. 28 figs 4–5.

##### Type locality.

“republica Peruviana”.

##### Label.

“Pérou”, in d’Orbigny’s handwriting.

##### Dimensions.

“Longit. 52 millim., latit. 25 millim.”. Figured specimen H 51.9, D 25.1, W 5.6.

##### Type material.

NHMUK 1854.12.4.117, lectotype (d’Orbigny coll.).

##### Remarks.

d’Orbigny did not state on how many specimens his description was based. The specimen found corresponds to the figures of d’Orbigny and is here designated lectotype (**design. n.**) to define the taxon, which has been compared to *Bulimus taeniolus* Nyst, 1845 (Pilsbry 1895 [1895–1896]: 57). d’Orbigny (1838 [1834–1847]: 294) specified the type locality as follows: “Nous n’avons pas receuilli nous-même ce Bulime; nous le devons à la bonté toute particulière de M. Mathius, botaniste anglais, que nous avons rencontré à Lima, et qui l’avait apporté du verant oriental des Andes péruviennes, à peu près par la latitude de Lima”; this would indicate the eastern part of Dept. Junín (e.g., Chanchamayo region) as the likely source area. The classification at species level follows Richardson (1995: 373), but further studies are needed to ascertain its status; for the generic level see also the remarks under the systematic arrangement above.

##### Current systematic position.

?Orthalicidae, *Scholvienia brephoides* (d’Orbigny, 1835).

#### Orthalicus (Porphyrobaphe) buckleyi

Taxon classificationAnimaliaStylommatophoraOrthalicidae

Higgins, 1872

Figs 3iv–v , L3iii

Orthalicus (Porphyrobaphe) buckleyi Higgins 1872: 685, pl. 56 fig. 3.
Orthalicus (Methorthalicus) buckleyi ; Pilsbry 1899: 193, pl. 41 fig. 6.
Thaumastus (Thamastus) buckleyi ; Breure 1978: 27; Breure 1979: 44; Breure and Borrero 2008: 8.

##### Type locality.

[Ecuador, Prov. Loja] “San Lucas”.

##### Label.

“Ecuador”.

##### Dimensions.

“Long. 93, lat. 36 mill.”. Figured specimen H 92.0, D 36.0, W 6.0.

##### Type material.

NHMUK 1872.5.22.6, two syntypes (da Costa coll.).

##### Remarks.

Higgins did not state on how many specimens his description was based. Of the two syntypes mentioned by Breure (1978), only one could be found.

##### Current systematic position.

Megaspiridae, *Thaumastus buckleyi* (Higgins, 1872).

#### Bulimus clouei

Taxon classificationAnimaliaStylommatophoraBulimulidae

Pfeiffer, 1857

Figs 21i–iii , L4i

Bulimus clouei Pfeiffer 1857d: 390; Pfeiffer 1859: 408; Breure 1979: 127.
Drymaeus clouei ; Pilsbry 1899: 94.
Leiostracus (Leiostracus) clouei ; Breure 1978: 227 (lectotype designation).
Leiostracus clouei ; Simone 2006: 121, fig. 377.

##### Type locality.

“Brazil (*Mr. Cloué*)”.

##### Label.

“Brazils Mons Cloué”, taxon label in Pfeiffer’s handwriting. M.C. label style IV.

##### Dimensions.

“Long. 22, diam. 10 mill.”. Figured specimen H 22.2, D 11.1, W 7.2.

##### Type material.

NHMUK 1975491, lectotype; 1975492, one paralectotype, Cloué leg. (Cuming coll.).

##### Remarks.

Pfeiffer did not state on how many specimens his description was based. The current systematic position follows Simone (2006).

##### Current systematic position.

Simpulopsidae, *Leiostracus clouei* (Pfeiffer, 1857).

#### Bulimus consimilis

Taxon classificationAnimaliaStylommatophoraBulimulidae

Reeve, 1848

Figs 1iii–iv , L4ii

Bulimus consimilis Reeve 1848 [1848–1850]: pl. 53 fig. 346.

##### Type locality.

“—?”.

##### Label.

“Brazil”. M.C. label style I, V.

##### Dimensions.

Not given. Figured specimen H 52.9, D 22.8, W 6.5.

##### Type material.

NHMUK 20030189, three syntypes (Cuming coll.).

##### Remarks.

Reeve did not state on how many specimens his description was based, but wrote “[t]his shell approaches nearest to the *Bulimus Taunaisii*, but is certainly distinct”. Pfeiffer (1853: 406) considered this taxon a junior subjective synonym of *Bulimus largillierti* Philippi, 1842, which has been followed by later authors. The printed label also mentions this name, and this is consistent with the index (Reeve 1850 [1848–1850]: v); the locality “Brazil” has been added in a later hand.

##### Current systematic position.

Megaspiridae, *Thaumastus largillierti* (Philippi, 1842).

#### Simpulopsis corrugatus

Taxon classificationAnimaliaStylommatophoraOrthalicidae

Guppy, 1866

Figs 24i–ii , L5i

Simpulopsis corrugatus Guppy 1866: 53; Breure 1979: 134.

##### Type locality.

“Trinidad”.

##### Label.

“Trinidad”, presumably in Guppy’s handwriting.

##### Dimensions.

“Height 0.38 inch, greatest breadth 0.47 inch [H 9.65, D 11.9 mm]”. Figured specimen H 9.27 (damaged), D 10.8, W 3.5.

##### Type material.

NHMUK 1866.1.3.7, one syntype (ex Guppy).

##### Remarks.

Guppy did not state on how many specimens his description was based; the single specimen found is damaged. Guppy emendated the name “qu’il me paraît préférable de féminiser, à l’exemple de Pfeiffer et de la plupart des auteurs” (Guppy 1878: 323). He also wrote “...je n’avais pu trouver que deux individus complétement adultes et deux exemplaires jeunes de cette espèce (...) Au commencement de l’année 1877, j’ai été assez heureux pour découvrir six à sept autres individus adultes (...) Coll. L. Guppy et H. Crosse”. From this text it is clear that Guppy had multiple specimens at hand when originally describing this taxon, and also that the figure presented in this paper (Guppy 1878: pl. 10 fig. 5) is likely not from the type series.

##### Current systematic position.

Simpulopsidae, Simpulopsis (Simpulopsis) corrugata Guppy, 1866.

#### Simpulopsis cumingi

Taxon classificationAnimaliaStylommatophoraOrthalicidae

Pfeiffer, 1861

Figs 24iii–vi , L5ii

Simpulopsis cumingi Pfeiffer 1861a [April]: 84; Pfeiffer 1861b [May]: 27, pl. 3 fig. 2; Reeve 1862 [1860–1862]: pl. 1 fig. 5; Pfeiffer 1868a: 22; Pilsbry 1899: 220, pl. 63 figs 61–62; Breure 1979: 134.

##### Type locality.

“Mexico”.

##### Label.

“Mexico”, taxon label in Pfeiffer’s handwriting. M.C. label style III.

##### Dimensions.

“Diam. maj. 20 1/2, alt. 12 mill.”. Figured specimen H 14.1, D 19.0, W 3.4.

##### Type material.

NHMUK 1975486, lectotype and one paralectotype (Cuming coll.).

##### Remarks.

Pfeiffer did not state on how many specimens his description was based. Two specimens have been found in the collection, of which one is designated lectotype (**design. n.**) to fixate this poorly understood species. This taxon was compared to *Simpulopsis aenea* by Pilsbry (1899), but has not been recognised by later authors. Richardson’s references (1995: 363) to a citation for Venezuela [Richards and Wagenaar Hummelinck 1940: 7] and Brazil [Jaeckel 1952: 7] were in error; these authors mentioned “*Tomigerus cumingi* Pfeiffer” [Odontostomidae]. The current systematic position is according to Thompson (2011: 130), who expressed doubt about the locality from which it was reported.

##### Current systematic position.

Simpulopsidae, Simpulopsis (Simpulopsis) cumingi Pfeiffer, 1861.

#### Bulimus deburghiae

Taxon classificationAnimaliaStylommatophoraBulimulidae

Reeve, 1859

Figs 18i–ii , L5iii

Bulimus deburghiae Reeve 1859: 123; Pfeiffer 1868b: 15.
Sultana (Metorthalicus) deburghiae ; Breure and Schouten 1985: 27 (lectotype designation).

##### Type locality.

“Peruvian side of the Amazon”.

##### Label.

“Banks of Amazon River / (Reeve)”, in Dance’s handwriting; see below.

##### Dimensions.

“Long. 2 3/4 in. Lat. 1 1/4 in. [H 69.9, D 31.8 mm]”. Figured specimen H 64.7, D 33.6, W 6+.

##### Type material.

NHMUK 19601622, lectotype (ex DeBurgh).

##### Remarks.

Reeve wrote “[a] fine shell”, but otherwise it is not clear from the context that he had only one specimen at hand. The material is accompanied by a label written in 1961 by S.P. Dance “This specimen does not suit Reeve’s measurements but it is labelled by Mrs. de Burgh”; his selection as lectotype was interpreted as such by Breure and Schouten (1985). Their text may be ambiguous, but as all the qualifying data are given following Recommendation 74C jo. 73C, we feel that this designation qualifies Art. 74.5 ICZN. The specimen is slightly damaged at the top, hence the measurements depart from those given by Reeve.

##### Current systematic position.

Orthalicidae, Sultana (Metorthalicus) deburghiae (Reeve, 1859).

#### Simpulopsis decussata

Taxon classificationAnimaliaStylommatophoraOrthalicidae

Pfeiffer, 1857

Figs 25iv–vii , L5iv

Simpulopsis decussata Pfeiffer 1857a: 260; Breure 1978: 232; Breure 1979: 134 (lectotype designation); Simone 2006: 179, fig. 642; da Silva and Thomé 2007: 11, figs 16–17.

##### Type locality.

[Brazil] “Petropolis prope Rio Janeiro (Miers)”.

##### Label.

“Petropolis Rio / F. Miers E[sq.]”, taxon label in Pfeiffer’s handwriting. M.C. label style III.

##### Dimensions.

“Diam. maj. 12 1/2, alt. 11 mill.”. Figured specimen H 14.3, D 12.5, W 4.7.

##### Type material.

NHMUK 1975488, lectotype (Cuming coll.).

##### Remarks.

Pfeiffer did not state on how many specimens his description was based, but described this taxon from “Mus. Cuming”. This is contrasting the statement in da Silva and Thomé (2007), who said “Pfeiffer mentioned a single specimen”; they considered the specimen in NHMUK as the holotype (da Silva and Thomé 2007: 14), but this does not follow Art. 73.1 and Recommendation 73F ICZN Code. The current systematic position follows Simone (2006).

##### Current systematic position.

Simpulopsidae, Simpulopsis (Simpulopsis) decussata Pfeiffer, 1857.

#### Bulimus demerarensis

Taxon classificationAnimaliaStylommatophoraBulimulidae

Pfeiffer, 1861

Figs 21vi–viii , L6ii

Bulimus demerarensis Pfeiffer 1861a [April]: 14; Pfeiffer 1861b [May]: 24; Breure 1979: 127.
Drymaeus demerarensis ; Pilsbry 1898 [1897–1898]: 306.
Leiostracus (Leiostracus) demerarensis ; Breure 1978: 227 (lectotype designation).
Bostryx demerarensis ; Muratov and Gargominy 2011: 612, fig. 2B.

##### Type locality.

[Guiana] “Demerara”.

##### Label.

“Demerara”, taxon label in Pfeiffer’s handwriting. M.C. label style IV.

##### Dimensions.

“Long. 20 1/2, diam. 10 mill.”. Figured specimen H 20.1, D 10.9, W 6.5.

##### Type material.

NHMUK 1975501, lectotype (Cuming coll.).

##### Remarks.

Pfeiffer did not state on how many specimens his description was based; only a single specimen was found. Muratov and Gargominy (2011) re-described this taxon and studied the anatomy of a single dried individual. They concluded that this taxon “lacks the very characteristic, for *Leiostracus*, division of the spermathecal duct into an enlarged distal part and a slender proximal part that connect to the distal part sub-apically, which is essentially the only character that separates *Leiostracus* from *Bostryx*”. As Breure (1978: 239–240) has shown, these two genera also differ in their radula structure, which was not studied by Muratov and Gargominy. Moreover, molecular data lends support for clear differentiation of both genera, even in different families (Breure and Romero 2012); more research may be needed to ascertain the position of Pfeiffer’s taxon. The generic classification of Breure (1979) is retained herein.

##### Current systematic position.

Simpulopsidae, *Leiostracus demerarensis* (Pfeiffer, 1861).

#### Pupa (Megaspira) elata

Taxon classificationAnimaliaHeterostrophaActeonidae

Gould, 1847

Figs 2i–iv , L6iii

Pupa (Megaspira) elata Gould 1847: 197; Gould 1862: 34.
Megaspira elata ; Gould 1852: 91; Gould 1856: 5, pl. 7 fig. 101; Rehder 1945: 67 (lectotype designation); Simone 2006: 182, fig. 659.

##### Type locality.

“Brazil”.

##### Label.

“Brazil (Gould)”.

##### Dimensions.

“Long. 1 1/2, lat. 1/3 poll. [H 38.0, D 8.4 mm]”. Figured specimen H 37.2, D 8.4, W 18.1.

##### Type material.

NHMUK 1987060, three paralectotypes (ex Gould).

##### Remarks.

Gould did not state on how many specimens his description was based. The lectotype is USNM 5503 (Rehder 1945), who considered this taxon to be a junior synonym of *Pupa elatior* Spix, 1827; however, Simone (2006) considered the two taxa as distinct and the current systematic position follows his work.

##### Current systematic position.

Megaspiridae, *Megaspira elata* (Gould, 1847).

#### Bulimulus ephippium

Taxon classificationAnimaliaStylommatophoraOrthalicidae

Ancey, 1904

Figs 28iv–vi , L6iv

Bulimulus ephippium Ancey 1904: 102; Breure 1979: 62; Simone 2006: 118, fig. 361; Wood and Gallichan 2008: 44; Breure 2011: 25, fig. 16C, 16ii (lectotype designation).
?Bulimulus ephippium ; Breure 1978: 144, pl. 11 fig. 8.

##### Type locality.

“Bahia, Brazil (*teste* H. Fulton)”.

##### Label.

“Bahia”, in Fulton’s handwriting.

##### Dimensions.

“Longit. 20, diam. 12 mill.”. Figured specimen H 20.5, D 13.3, W 5.3.

##### Type material.

NHMUK 1905.12.30.12, one paralectotype.

##### Remarks.

Ancey did not state on how many specimens his description was based; the NHMUK specimens were considered syntypes by Breure (1978, 1979), and Simone (2006). Breure (2011) selected the specimen in RBINS (also mentioned as syntype by Wood and Gallichan 2008) as lectotype. Ancey (1904) considered his taxon as belonging to “the *Eudioptus* section” of *Bulimulus*; Breure (2011) re-classified it with Simpulopsis (Eudioptus) Albers, 1860. Further anatomical and molecular studies should provide more evidence for this classification.

##### Current systematic position.

Simpulopsidae, Simpulopsis (Eudioptus) ephippium Ancey, (1904).

#### Bulimus foveolatus

Taxon classificationAnimaliaStylommatophoraBulimulidae

Reeve, 1849

Figs 1v–vi , L7i

Bulimus mahogani Pfeiffer 1841: 42; Pfeiffer 1844 in Küster and Pfeiffer 1840–1865: 40, pl. 13 figs 1–2; Pfeiffer 1848b: 24. Not *Bulinus mahogani* Sowerby, 1838. See remarks.
Bulimus foveolatus Reeve 1849 [1848–1850]: pl. 73 fig. 526; Pfeiffer 1853 in Küster and Pfeiffer 1840–1865: xiv; Breure 1979: 44 (lectotype designation).
Strophocheilus foveolatus ; Pilsbry 1895 [1895–1896]: 46, pl. 24 fig. 71.

##### Type locality.

“Vitoe, near Sarma [sic, Tarma], Alto-Peru; W. Lobb”.

##### Label.

“Peru”. M.C. label style IV, V.

##### Dimensions.

“Long. 3 poll., diam. 15 lin. [H 76.0, D 31.7 mm]”; see remarks. Figured specimen H 71.5, D 37.0, W 5.7.

##### Type material.

NHMUK 1975275, lectotype; 1975276, one paralectotype (Cuming coll.).

##### Remarks.

Pfeiffer, in his original description, referred to Sowerby I 1838 in Sowerby I and II 1832–1841: fig. 59, for which no further data were presented; Pfeiffer (1848b) corrected the dimensions to “Long. 72, diam. 35 mill.”. In both instances the locality was presented as “Chile”, and the material as collected by Philippi (jun.); in Pfeiffer 1844 (Küster and Pfeiffer 1840–1865) “Aufenthalt: Chile und Peru” is given. The shell figured in the latter publication may be referred to what Reeve (1849) has named as *Bulimus foveolatus*; Pfeiffer (1853: xiv) remarked that his taxon was not identical to Sowerby’s *Bulinus mahogani*, however, he did not discuss the large geographical distance between the localities where Philippi and Lobb collected their material. Pfeiffer’s original material is most probably lost (Dance 1966), and whether his taxon is a synonym of Reeve’s may possibly never be fully ascertained. The current systematic position follows Richardson (1995: 375), who incorrectly assigned this taxon to Pfeiffer.

##### Current systematic position.

Megaspiridae, *Thaumastus foveolatus* (Reeve, 1849).

#### Bulimus fraseri

Taxon classificationAnimaliaStylommatophoraBulimulidae

Pfeiffer, 1858

Figs 19i–ii , L7iii

Bulimus fraseri Pfeiffer 1858: 239; Pfeiffer 1860: 137, pl. 51 fig. 5; Pfeiffer 1860 [1860–1866]: 157, pl. 42 figs 1–2; Pfeiffer 1868: 15.
Orthalicus fraseri ; Pilsbry 1899: 193, pl. 46 figs 31–33.
Sultana (Metorthalicus) fraseri ; Breure and Schouten 1985: 28 (lectotype designation).

##### Type locality.

“in provincia Cuenca reipublicae Aequatoris (Fraser)”.

##### Label.

“Found on the road from Gualaquiza / to Mendez— and near to the latter / place”, “Province of Cuenca / Republic of Ecuador / M^r^ Fraser”, taxon label in Pfeiffer’s handwriting. M.C. label style III.

##### Dimensions.

“Long. 89, diam. 37 mill.”. Figured specimen H 88.9, D 45.0, W 6.4.

##### Type material.

NHMUK 20140083, lectotype, Fraser leg. (Cuming coll.).

##### Remarks.

Pfeiffer did not state on how many specimens his description was based, but described this taxon from “Mus. Cuming”. The current systematic position follows Breure and Schouten (1985).

##### Current systematic position.

Orthalicidae, Sultana (Metorthalicus) fraseri (Pfeiffer, 1858).

#### Bulimus gloriosus

Taxon classificationAnimaliaStylommatophoraBulimulidae

Pfeiffer, 1862

Figs 18iii–iv , L7iv

Bulimus gloriosus Pfeiffer 1862: 387, pl. 37 fig. 4; Pfeiffer 1868b: 14.
Sultana (Metorthalicus) deburghiae (Reeve); Breure and Schouten 1985: 27 (lectotype designation).

##### Type locality.

“Republic of Ecuador”.

##### Label.

“Republic Ecuador / M^r^ Fraser”, taxon label in Pfeiffer’s handwriting. M.C. label style III.

##### Dimensions.

“Long. 78, diam. 34 mill.”. Figured specimen H 75.2, D 39.3, W 5.7+.

##### Type material.

NHMUK 1975243, lectotype (Cuming coll.).

##### Remarks.

Pfeiffer did not state on how many specimens his description was based; the single specimen found has the top damaged. The lectotype designation by Breure and Schouten (1985) may be viewed ambiguously, but as all the qualifying data are given following Recommendation 74C jo. 73C, we feel that this designation qualifies Art. 74.5 ICZN. The current systematic position follows Breure and Schouten (1985).

##### Current systematic position.

Orthalicidae, Sultana (Metorthalicus) deburghiae (Reeve, 1859).

#### Simpulopsis gomesae

Taxon classificationAnimaliaStylommatophoraOrthalicidae

da Silva & Thomé, 2006

Figs 25i–iii

Simpulopsis gomesae da Silva and Thomé 2006: 191, figs 19–32.

##### Type locality.

“Brasil, Rio Grande do Sul, São Francisco de Paula”.

##### Label.

No locality.

##### Dimensions.

Not given (range H 1.60–10.96, D 1.55–8.63 mm). Figured specimen H 6.46, D 6.93, W 3.5.

##### Type material.

NHMUK 20050238, one paratype in ethanol, J.W. Thomé leg.

##### Remarks.

This taxon was based on 17 specimens; the specimen present in NHMUK was mentioned in the original paper. Its systematic position may, however, need to be critically re-examined as many taxa have already been described from this region.

##### Current systematic position.

Simpulopsidae, Simpulopsis (Simpulopsis) gomesae da Silva & Thomé, 2006.

#### Corona pfeifferi gracilis

Taxon classificationAnimaliaStylommatophoraOrthalicidae

E.A. Smith, 1902

Figs 9i–ii , L7ii

Corona pfeifferi gracilis E.A. Smith 1902: 170.

##### Type locality.

“Rio Caqueta, S.E. Colombia”.

##### Label.

“Rio Caqueta, / S.E. Colombia”, in Smith’s handwriting.

##### Dimensions.

“[L]ength is 67 mm. and diameter 23”. Figured specimen H 67.3, D 24.8, W 8.8.

##### Type material.

NHMUK 1902.5.27.4, holotype.

##### Remarks.

This taxon was described from a single specimen. The morphological variation within *Corona pfeifferi* needs further study.

##### Current systematic position.

Orthalicidae, *Corona pfeifferi gracilis* E.A. Smith, 1902.

#### Bulimus hartwegi

Taxon classificationAnimaliaStylommatophoraBulimulidae

Pfeiffer in Philippi, 1846

Figs 3i–iii , L8i

Bulimus hartwegi Pfeiffer in Philippi 1846 [1845–1847]: 111, pl. 4 fig. 1; Reeve 1848 [1848–1850]: pl. 29 fig. 176; Pfeiffer 1848b: 140; Breure 1979: 44.
Strophocheilus hartwegi ; Pilsbry 1895 [1895–1896]: 52, pl. 26 fig. 82.
Thaumastus (Thaumastus) hartwegi ; Breure 1978: 29; Breure and Borrero 2008: 9.

##### Type locality.

“respublica [sic] Aequatoris, ubi ad ‘El Catamaija’ prope Loxa Hartweg”.

##### Label.

“El Catamaja near Loxa”. M.C. label style IV.

##### Dimensions.

“Long 28, diam. 13´´´ [H 61.0, D 28.3 mm]”. Figured specimen H 57.0, D 30.0, W 4.8.

##### Type material.

NHMUK 1975126, one syntype (Cuming coll.).

##### Remarks.

Pfeiffer did not state on how many specimens his description was based, but said the material was in “Sammlung des Hrn. Hugh Cuming”. Given the context of the publication, it is here assumed that the dimensions were given in German lines (1 line = 2.18 mm); Pfeiffer (1848b) quoted “Long. 57, diam. 26 mill.”, which shell height concurs with our measurement given above. The current systematic position follows Richardson (1995: 376).

##### Current systematic position.

Megaspiridae, *Thaumastus hartwegi* (Pfeiffer in Philippi, 1846).

#### Bulimus hyaloideus

Taxon classificationAnimaliaStylommatophoraBulimulidae

Pfeiffer, 1855

Figs 23iii–iv , L8ii

Bulimus hyaloideus Pfeiffer 1855b: 292; Pfeiffer 1859: 505; Breure 1979: 131.
Rhinus constrictus (Pfeiffer); Breure 1978: 232 (lectotype designation).
Rhinus hyaloideus ; Linares and Vera 2012: 206.

##### Type locality.

“Mendez, Andes of New Granada”.

##### Label.

“Mendes Andes of Granada”, taxon label in Pfeiffer’s handwriting. M.C. label style I.

##### Dimensions.

“Long. 12 1/2, diam. 7 1/2 mill.”. Figured specimen H 20.6, D 11.1, W 6.7.

##### Type material.

NHMUK 1975412, lectotype; 1975413, one paralectotype (Cuming coll.).

##### Remarks.

Pfeiffer did not state on how many specimens his description was based; he described this taxon from Cuming’s collection. The paralectotype specimen is juvenile. Breure (1978) mentioned that the original measurements given by Pfeiffer were in error. Linares and Vera (2012) said “*Bulimulus hyaloides* (Pfeiffer, 1855) es un sinónimo”, overlooking the fact that this is the same taxon; they probably mixed the classification of Richardson (1995: 76) [who placed this taxon with *Bulimulus* Leach, 1814], and the classification of Breure (1979) [who placed it under *Rhinus* Albers, 1860]. The current systematic position follows the synonymisation by Breure (1978).

##### Current systematic position.

Simpulopsidae, *Rhinus constrictus* (Pfeiffer, 1841).

#### Helix inca

Taxon classificationAnimaliaStylommatophoraHelicidae

d’Orbigny, 1835

Figs 4iv–vi , L8iii

Helix inca d’Orbigny 1835: 16; Breure 1979: 44.
Bulimus inca ; d’Orbigny 1837 [1834–1847]: 294, pl. 38 figs 6–7 [text 6 May 1838]; Gray 1854: 18.
Strophocheilus inca ; Pilsbry 1895 [1895–1896]: 56, pl. 28 figs 10–11.
Thaumastus (Atahualpa) inca ; Breure 1975: 1139.

##### Type locality.

“Tutulima (republica Boliviana)”.

##### Label.

“Tutulima, Bolivia”, in d’Orbigny’s handwriting.

##### Dimensions.

“Longit 72 millim., latit. 30 millim.”. Figured specimen H 75.4, D 32.3, W 8.3.

##### Type material.

NHMUK 1854.12.4.116, lectotype and three paralectotypes (d’Orbigny coll.).

##### Remarks.

d’Orbigny did not state on how many specimens his description was based. The lot found consists of four specimens, of which the one corresponding to d’Orbigny’s figure is now designated lectotype (**design. n.**) to fixate the taxon, which needs further study to clarify its status; the three paralectotypes are one subadult and two juveniles. Three other specimens are in the MNHN collection (Breure 1975), and are thus paralectotypes. d’Orbigny (1838 [1834–1847]: 295) specified the localities as follows: “deux localités differentes, au nord-est de la Cordillère orientale de Bolivia; la première fois dans le fond d’un ravin humide et boisé, près de Carcuata, province de Yungas, où nous n’en avons eu qu’un seul exemplaire; puis au nord de Cochabamba, dans le fond du ravin de ‘Tutulima’, d’où il nous a été apportes par les Indiens” (see also Breure 1973). The current systematic position follows Richardson (1995: 376).

##### Current systematic position.

Megaspiridae, *Thaumastus inca* (d’Orbigny, 1835).

#### Strophocheilus (Dryptus) indentatus

Taxon classificationAnimaliaStylommatophoraStrophocheilidae

da Costa, 1901

Figs 8iii–iv , L9i

Strophocheilus (Dryptus) indentatus da Costa 1901: 239, pl. 24 fig. 8; Pilsbry 1902 [1901–1902]: 281, pl. 49 fig. 7.
Thaumastus (Thaumastus) indentatus ; Breure 1979: 44; Breure and Borrero 2008: 8.

##### Type locality.

“Ecuador”.

##### Label.

“Ecuador”, in da Costa’s handwriting.

##### Dimensions.

“Long. 44, diam. 23 mm.”. Figured specimen H 44.0, D 24.0, W 4.8.

##### Type material.

NHMUK 1907.11.21.115, lectotype; 1907.11.21.116, one paralectotype (da Costa coll.).

##### Remarks.

da Costa did not state on how many specimens his description was based. This species has been classified by *Thaumastus* s.str. by Breure (1979). Upon re-studying the specimens found, however, the protoconch appears to be pit-reticulated and the taxon may be better placed in *Kara* Strebel, 1910. This taxon is closely allied to *Kara thompsonii* (Pfeiffer, 1845) and *Kara yanamensis* (Morelet, 1863), and upon further studies may prove to be a synonym of either of these species.

##### Current systematic position.

Orthalicidae, *Kara indentata* (da Costa, 1901) (**comb. n.**).

#### Bulimus (Thaumastus) insolitus

Taxon classificationAnimaliaStylommatophoraBulimulidae

Preston, 1909

Figs 4i–iii , L9ii

Bulimus (Thaumastus) insolitus Preston 1909: 509, pl. 10 fig. 9; Breure 1979: 44.

##### Type locality.

“Chanchamayo, Peru”.

##### Label.

“Chanchamayo Peru”.

##### Dimensions.

“Alt. 70, diam. maj. 29.5 mm.”. Figured specimen H 70.4, D 31.2, W 5.6.

##### Type material.

NHMUK 1947.3.11.1, holotype (ex Preston).

##### Remarks.

Preston wrote “[a]n extraordinary shell”; the singular implies that he had only one specimen at hand, the specimen thus is the holotype. A label states “Purchased from / Preston many years ago / by Mayor Connolly with / others / A. M. N. H. viii p. 509”. The current systematic position follows Richardson (1995: 377) at the species level, the generic classification should be re-evaluated by further studies of the anatomy and by molecular research; this could also affect the arrangement at family level.

##### Current systematic position.

Megaspiridae, *Thaumastus insolitus* (Preston, 1909).

#### Bulimus integer

Taxon classificationAnimaliaStylommatophoraBulimulidae

Pfeiffer, 1855

Figs 5i–iii , L10i

Bulimus integer Pfeiffer 1855d: 114; Pfeiffer 1859: 369; Breure 1979: 44.
Porphyrobaphe integer ; Pilsbry 1899: 153.
Thaumastus (Thaumastus) integer ; Breure 1978: 31 (lectotype designation); Breure and Borrero 2008: 8.

##### Type locality.

“Quito, Ecuador”.

##### Label.

“Quito”. M.C. label style IV.

##### Dimensions.

“Long. 82, diam. 39 mill.”. Figured specimen H 81.5, D 42.0, W 7.4.

##### Type material.

NHMUK 1975244, lectotype; 1975245, one paralectotype (Cuming coll.).

##### Remarks.

Pfeiffer did not state on how many specimens his description was based; he did, however, recognize a variety β for which he gave “Long. 65, diam. 31 mill.” as measurements. This was likely a shell from his own collection, as the paralectotype in the Cuming collection has a shell height of 71.6 mm. The protoconch of these type specimens is sculptured with axial wrinkles, becoming coarse granules on the lower part of the protoconch. The generic classification of Breure (1979) is herein tentatively retained, but further studies should clarify the systematic position of this taxon.

##### Current systematic position.

Megaspiridae, *Thaumastus integer* (Pfeiffer, 1855).

#### Bulimus iris

Taxon classificationAnimaliaStylommatophoraBulimulidae

Pfeiffer, 1853

Figs 14iii–v , L10ii

Bulimus iris Pfeiffer 1853b: 313; Pfeiffer 1854b: 136; Pfeiffer 1855 in Küster and Pfeiffer 1840–1865: 244, pl. 65 figs 4–5.
Porphyrobaphe iris ; Pilsbry 1899: 157, pl. 51 figs 28–29.

##### Type locality.

“Le Ceja, Rio Negro Novae Granadae (Bland)”.

##### Label.

“La Ceja. Rio Negro / New Grenada”, taxon label in Pfeiffer’s handwriting. M.C. label style I.

##### Dimensions.

“Long. 64, diam. 32 mill.”. Figured specimen H 72.6, D 41.1, W 5.8.

##### Type material.

NHMUK 20100506, lectotype (Cuming coll.).

##### Remarks.

Pfeiffer did not state on how many specimens his description was based, but described the species from “Mus. Cuming”. Although the specimen is larger than the measurements given by Pfeiffer, there is little doubt it is from the original series as the label confirms the original locality; it also bears the text “please to name this / ‘*Blandi*’ after the collector”. The specimen is now designated lectotype (**design. n.**) to fixate this taxon, which needs further study to clarify its status. The mentioning in Linares and Vera (2012: 157) of “ZMUZ 511864” as lectotype for this taxon is erroneous, as this refers to the type specimen of the junior subjective synonym *Bulimus wallisianus* Mousson, 1873 (see Breure 1976: 3). The current systematic position follows Richardson (1993: 119).

##### Current systematic position.

Orthalicidae, Porphyrobaphe (Porphyrobaphe) iris (Pfeiffer, 1853).

#### Bulimus irroratus

Taxon classificationAnimaliaStylommatophoraBulimulidae

Reeve, 1849

Figs 15i–ii , L10iii

Bulimus irroratus Reeve 1849 [1848–1850]: pl. 62 fig. 427; Reeve 1850a: 16; Pfeiffer 1853b: 304.
Porphyrobaphe irrorata ; Pilsbry 1899: 155, pl. 51 figs 36–37; Breure and Schouten 1985: 41 (lectotype designation).

##### Type locality.

“Brazil? New Granada?”.

##### Label.

“Quito Ecuador”, taxon label in Pfeiffer’s handwriting. M.C. label style III.

##### Dimensions.

Not given. Figured specimen H 77.0, D 44.0, W 6+.

##### Type material.

NHMUK 1975248, three syntypes, A.L. Gubba leg. (Cuming coll.).

##### Remarks.

Reeve did not state on how many specimens his description was based, but said it was the “Mus. Cuming (...) thanks to the liberality of A.L. Gubba, Esq., of [Le] Havre”. In Reeve (1850) the locality was mentioned as “—?”. The top of the specimen figured is damaged. The current systematic position is according to Richardson (1993: 119).

##### Current systematic position.

Orthalicidae, *Porphyrobaphe irrorata* (Reeve, 1849).

#### Bulimus jeffreysi

Taxon classificationAnimaliaStylommatophoraBulimulidae

Pfeiffer, 1852

Figs 21iv–v , L11i

Bulimus jeffreysi Pfeiffer 1852: 93; Pfeiffer 1853b: 342; Pfeiffer 1854 in Küster and Pfeiffer 1840–1865: 187, pl. 49 figs 9–10.
Drymaeus obliquus (Reeve); Pilsbry 1899: 93, pl. 14 fig. 15.
Leiostracus obliquus ; Simone 2006: 122, fig. 381.

##### Type locality.

“Brasilia”.

##### Label.

“Brazils”, taxon label in Pfeiffer’s handwriting. M.C. label style I.

##### Dimensions.

“Long. 19, diam. 11 mill.”. Figured specimen H 20.4, D 10.9, W 6+.

##### Type material.

NHMUK 20110083, three syntypes (Cuming coll.).

##### Remarks.

Pfeiffer did not state on how many specimens his description was based; he described the taxon “Ex Coll. Cl. Gruner” (Pfeiffer 1852) and specified this later to “Mus. Cuming ex Gruner” (Pfeiffer 1853b). The three specimens found are thus considered to be syntypes; one of these, possibly figured in Pfeiffer 1854 [Küster and Pfeiffer 1840–1865], is broken. The current systematic position follows Simone (2006).

##### Current systematic position.

Simpulopsidae, *Leiostracus obliquus* (Reeve, 1849).

#### Bulimus kelletti

Taxon classificationAnimaliaStylommatophoraBulimulidae

Reeve, 1850

Figs 19iii–iv , L11ii

Bulimus kelletti Reeve 1850 [1848–1850]: pl. 89 fig. 661; Pfeiffer 1853b: 305.
Orthalicus kellettii ; Pilsbry 1899: 204, pl. 45 figs 23–24.
Sultana (Metorthalicus) kellettii ; Breure and Schouten 1985: 28 (lectotype designation); Breure and Borrero 2008: 26.

##### Type locality.

“Ecuador?”.

##### Label.

“?Ecuador”. M.C. label style III, V.

##### Dimensions.

Not given. Figured specimen H 61.2, D 33.2, W 5.7.

##### Type material.

NHMUK 1975241, lectotype (Cuming coll.).

##### Remarks.

The material is accompanied by a label “the type specimen”. However, Reeve did not state on how many specimens his description was based, but only mentioned “this new and very beautiful species”. The specimen found should thus be regarded as lectotype, contradicting the statement by Breure and Borrero (2008), who considered it as holotype.

##### Current systematic position.

Orthalicidae, Sultana (Metorthalicus) kelletti (Reeve, 1850).

#### Bulimus loxostomus

Taxon classificationAnimaliaStylommatophoraBulimulidae

Pfeiffer, 1853

Figs 5iv–vi , L11iii

Bulimus loxostomus Pfeiffer 1853b: 379; Pfeiffer 1854a: 59.
Strophocheilus loxostomus ; Pilsbry 1895 [1895–1896]: 52.
Thaumastus (Thaumastus) loxostomus ; Linares and Vera 2012: 206.

##### Type locality.

“in Andibus Novae Granadae”.

##### Label.

“Andes N. Granada”. M.C. label style III.

##### Dimensions.

“Long. 71, diam. 34 mill.”. Figured specimen H 71.3, D 37.3, W 5.8.

##### Type material.

NHMUK 1975125, one syntype (Cuming coll.).

##### Remarks.

Pfeiffer did not state on how many specimens his description was based, but said the material was in “Mus. Cuming”. The protoconch is sculptured with spaced, indistinct wrinkles, becoming closer towards the transition to the teleoconch. The lip is white, which is quite unusual for *Thaumastus* *s.str.* Further research should thus shed more light on the systematic position of this taxon, which is here figured for the first time. Linares and Vera (2012) assumed that this taxon was collected in “Colombia, en una localidad no definida”. Although this cannot be excluded, this remains disputable as ‘New Granada’ had a broader political-administrative meaning at the time the specimen was collected. Therefore, at the moment the allocation of this taxon to the Colombian malacofauna remains doubtful at best.

##### Current systematic position.

Megaspiridae, *Thaumastus loxostomus* (Pfeiffer, 1853).

#### Achatina magnifica

Taxon classificationAnimaliaStylommatophoraAchatinidae

Pfeiffer, 1848

Figs 7i–ii , L11iv

Achatina magnifica Pfeiffer 1848a: 232; Pfeiffer 1848b: 255.
Liguus (Hemibulimus) magnificus ; E.A. Smith 1907: 314, fig.

##### Type locality.

“Quito, Ecuador; in woods (De Lattre)”.

##### Label.

“Quito, Ecuador”, taxon label in Pfeiffer’s handwriting. M.C. label style IV.

##### Dimensions.

“Long. 47, diam. 21 mill.”. Figured specimen H 46.6, D 23.0, W 5.5.

##### Type material.

NHMUK 20100508, two syntypes (Cuming coll.).

##### Remarks.

Pfeiffer did not state on how many specimens his description was based, but described this taxon from Cuming’s collection. Reeve’s figure (Reeve 1849 [1849–1850]: pl. 9 fig. 33) was from “the collection of J. Dennison, Esq., of which there is also a specimen in the possession of Mr. Cuming [i.e., Pfeiffer’s type]”. This taxon has been incorrectly classified with *Hemibulimus* Martens, 1885 by Pilsbry (1899: 185)—who copied Reeve’s figure—and Richardson (1993: 71); Pilsbry (1909 [1908–1910]: 117) corrected his mistake. The type material is here re-figured, after E.A. Smith (1907) had figured it for the first time. Although the specimen seems to be slightly subadult, this taxon might be closely allied to *Bulimus corydon* Crosse, 1869, *Bulimus phoebus* Pfeiffer, 1863, and *Bulimus victor* Pfeiffer, 1854. *Achatina magnifica* is now tentatively placed in *Clathrorthalicus* Strebel, 1909; however, further anatomical and molecular studies should reveal the correct systematic position.

##### Current systematic position.

Orthalicidae, *Clathrorthalicus magnificus* (Pfeiffer, 1848) (**comb. n.**).

#### Bulimus magnificus

Taxon classificationAnimaliaStylommatophoraBulimulidae

Grateloup, 1839

Figs 6i–iii , L12i

Bulimus magnificus Grateloup 1839a: 165; Grateloup 1839b: 419, pl. 4 fig. 1; Breure 1979: 44.
Strophocheilus magnificus ; Pilsbry 1895 [1895–1896]: 46, pl. 25 fig. 74.
Thaumastus (Thaumastus) magnificus ; Breure 1978: 31 (lectotype designation).
Thaumastus magnificus ; Simone 2006: 153, fig. 521.

##### Type locality.

“Pérou”.

##### Label.

“Le Perou (Brésil)”; see remarks.

##### Dimensions.

“Près de 3 pouces de longueur”; see remarks. Figured specimen H 78.0, D 36.0, W 6.9.

##### Type material.

NHMUK 1907.11.22.24, lectotype (da Costa coll., ex Grateloup).

##### Remarks.

Grateloup did not state on how many specimens his description was based; in Grateloup (1839b: 420) he gave as measurements “Hauteur: 80 mill. – Diamètre: 35 Mill.” and said it was from “Mon cabinet”. As Breure (1978) noted, this specimen “From Grateloup Coll^n^.” came to the NHMUK collection via da Costa, who purchased the specimen from the dealers Sowerby and Fulton. Reeve (1848 [1848–1850]) evidently based his description on a different specimen, as he wrote “The shell named *Bulimus magnificus* by M. Grateloup is, according to the specimen so marked in Mr. Cuming’s collection, a variety of *Bulimus Taunaysii* [supposed by Reeve to be Férussac’s species] of a lighter brown colour”. Despite the confusing localities (“Brésil” seems to be added in a later hand), the status of this specimen is not disputed herein; the Peruvian locality, however, still needs confirmation. The current systematic position follows Simone (2006), who reported this species from the states of Rio de Janeiro and São Paulo.

##### Current systematic position.

Megaspiridae, *Thaumastus magnificus* (Grateloup, 1839).

#### Bulimus marmatensis

Taxon classificationAnimaliaStylommatophoraBulimulidae

Pfeiffer, 1855

Figs 28i–iii , L12ii

Bulimus marmatensis Pfeiffer 1855a: 125; Pfeiffer 1859: 501.
Bulimulus marmatensis ; Pilsbry 1897 [1897–1898]: 61; Linares and Vera 2012: 163.

##### Type locality.

[Colombia] “Marmato, New Granada”.

##### Label.

“Marmata / New Grenada”, taxon label in Pfeiffer’s handwriting. M.C. label style III.

##### Dimensions.

“Long. 17, diam. 10 mill.”. Figured specimen H 15.0, D 11.0, W 5.0.

##### Type material.

NHMUK 1975330, three syntypes (Cuming coll.).

##### Remarks.

Pfeiffer did not state on how many specimens his description was based; he, however, described this taxon from the collection of Cuming. Three specimens were found, two damaged adults and one juvenile. The protoconch is sculptured with axial wrinkles and spiral lines; this taxon—classified by Breure (1979: 63) with *Bulimulus* Leach, 1814—is therefore now placed in *Simpulopsis* Beck, 1837 and is considered as junior subjective synonym of Simpulopsis (Eudioptus) citrinovitrea (Moricand, 1836) (**comb. n., syn. n.**). The taxon is here figured for the first time.

##### Current systematic position.

Simpulopsidae, Simpulopsis (Eudioptus) citrinovitrea (Moricand, 1836).

#### Orthalicus mars

Taxon classificationAnimaliaStylommatophoraOrthalicidae

Pfeiffer, 1861

Figs 13v–vi , L13i

Orthalicus mars Pfeiffer 1861b: 25, pl. 2 fig. 8; Pfeiffer 1868b: 202; Pilsbry 1899: 143, pl. 53 fig. 42.

##### Type locality.

“republica Aequatoris (Mr. Fraser)”.

##### Label.

“Republic of Ecuador”, taxon label in Pfeiffer’s handwriting. M.C. label style IV.

##### Dimensions.

“Long. 77, diam. 35 mill.”. Figured specimen H 76.6, D 38.4, W 6+.

##### Type material.

NHMUK 20100504, three syntypes (Cuming coll.).

##### Remarks.

Pfeiffer did not state on how many specimens his description was based, but described “from the collection of H. Cuming”. Although on the label has been written in a later hand “none quite like fig.”, the type status is not disputed as the shell height matches the original data. The top of the largest specimen, herein figured, is damaged. Also the top of one of the other specimens is damaged. The protoconch of the third, undamaged, specimen is smooth. This taxon is tentatively classified with *Orthalicus* Beck, 1837; however, further anatomical and molecular research should provide evidence to assess if this classification is correct or needs to be adjusted.

##### Current systematic position.

Orthalicidae, *Orthalicus mars* Pfeiffer, 1861.

#### Bulimus meobambensis

Taxon classificationAnimaliaStylommatophoraBulimulidae

Pfeiffer, 1855

Figs 17iii–iv , L13ii

Bulimus meobambensis Pfeiffer 1855c: 96; Pfeiffer 1859: 586.
Orthalicus meobambensis ; Pilsbry 1899: 191.

##### Type locality.

“Meobamba, Eastern Peru (Mr. Yates)”.

##### Label.

“Meobamba / East Peru”, taxon label in Pfeiffer’s handwriting. M.C. label style IV.

##### Dimensions.

“Long. 88, diam. 46 mill.”. Figured specimen H 84.9, D 52.8, W 6.4.

##### Type material.

NHMUK 20100505, two syntypes (Cuming coll.).

##### Remarks.

Pfeiffer did not state on how many specimens his description was based. There is no doubt, however, about the type status of the specimens found as he described this taxon from the Cuming collection and the taxon label is—although in pencil—in his handwriting. This is the first time this material is figured; Strebel (1909: pl. 29 fig. 429) figured a specimen from Huagabamba, Peru that E.A. Smith considered conspecific.

##### Current systematic position.

Orthalicidae, Sultana (Sultana) meobambensis (Pfeiffer, 1855).

#### Simpulopsis miersi

Taxon classificationAnimaliaStylommatophoraOrthalicidae

Pfeiffer, 1857

Figs 27iv–vi , L13iii

Simpulopsis miersi Pfeiffer 1857a: 260; Pfeiffer 1859: 800; Reeve 1862: pl. 1 fig. 4; Pilsbry 1899: 218; Breure 1979: 134 (lectotype designation); Simone 2006: 179, fig. 644.

##### Type locality.

[Brazil] “Espirito Santo Brasiliae (Miers)”.

##### Label.

“Espirits Santo / F. Miers Esq.”, taxon label in Pfeiffer’s handwriting. M.C. label style III.

##### Dimensions.

“Diam. maj. 24, alt. 17 1/2 mill.”. Figured specimen H 20.6, D 20.9, W 4.5.

##### Type material.

NHMUK 1975489, lectotype; 1975490 one paralectotype (Cuming coll., ex Miers).

##### Remarks.

Pfeiffer did not state on how many specimens his description was based, but refers to “Mus. Cuming”, and Miers as source. The lectotype is slightly damaged at the body of the last whorl and the lip. The references of Richardson (1993: 364) for this taxon to Pfeiffer 1853b: 333 and Pfeiffer 1859: 396 are erroneous, as these refer to *Bulinus miersii* Sowerby, 1838. The current systematic position is according to Simone (2006).

##### Current systematic position.

Simpulopsidae, Simpulopsis (Simpulopsis) miersi Pfeiffer, 1857.

#### Achatina murrea

Taxon classificationAnimaliaStylommatophoraAchatinidae

Reeve, 1849

Figs 9iv–vi , L14ii

Achatina murrea Reeve 1849 [1849–1850]: pl. 7 fig. 22.

##### Type locality.

“—?”.

##### Label.

No locality label, taxon label in Pfeiffer’s handwriting. M.C. label style IV, V.

##### Dimensions.

Not given. Figured specimen H 38.6, D 19.5, W 7.1.

##### Type material.

NHMUK 1975482, 20230332, three + three syntypes (Cuming coll.).

##### Remarks.

Reeve did not state on how many specimens his description was based, but figured two different specimens from “Mus. Cuming”. Three specimens were found in lot NHMUK 1975482, one of which corresponds to Reeve's figure 22a; Pfeiffer has identified this lot as “A. fasciata / Müller juv.”. Lot 20120332 also contains three specimens, one of which was figured as fig. 22b. The current systematic position follows Breure et al. (2014).

##### Current systematic position.

Orthalicidae, *Liguus murreus* (Reeve, 1849).

#### Orthalicus powissianus niveus

Taxon classificationAnimaliaStylommatophoraOrthalicidae

Preston, 1909

Figs 17i–ii , L14iii

Orthalicus powissianus var. niveus Preston 1909: 512.

##### Type locality.

“Jimenez, Rio Dagua, West Colombia”.

##### Label.

“Jimenez Rio Dagua / 1600 ft. Colombia”, in Preston’s handwriting.

##### Dimensions.

Not given. Figured specimen H 65.2, D 31.3, W 7.2.

##### Type material.

NHMUK 1909.8.18.85, holotype, M.G. Palmer leg., ex Preston.

##### Remarks.

Preston mentioned “taken with the animal alive”, from which may be inferred that he had only one specimen at hand. The specimen located is thus the holotype; the top is slightly damaged. The taxon is here figured for the first time. The current systematic position at species level follows Richardson (1993: 125).

##### Current systematic position.

Orthalicidae, Sultana (Methorthalicus) powisiana (Petit de la Saussaye, 1843).

#### Bulimus obliquus

Taxon classificationAnimaliaStylommatophoraBulimulidae

Reeve, 1849

Figs 22i–iii , L14iv

Bulimus obliquus Reeve 1849 [1848–1850]: pl. 76 fig. 551; Pfeiffer 1853b: 342; Breure 1979: 127.
Drymaeus obliquus ; Pilsbry 1899: 93, pl. 14 fig. 14.
Leiostracus (Leiostracus) obliquus ; Breure 1978: 227 (lectotype designation).
Leiostracus obliquus ; Simone 2006: 122, fig. 381.

##### Type locality.

[Brazil] “Bahia”.

##### Label.

“Brazil”. M.C. label style I, V.

##### Dimensions.

Not given. Figured specimen H 22.7, D 12.05, W 6+.

##### Type material.

NHMUK 1975493, lectotype (Cuming coll.).

##### Remarks.

Reeve did not state on how many specimens his description was based, but mentioned “[a] pink shell”; this is herein not considered as sufficient evidence that he had only one shell for his description. The material was in “Mus. Cuming”. The top and the apertural lip of the specimen found are damaged. The current systematic position follows Simone (2006).

##### Current systematic position.

Simpulopsidae, *Leiostracus obliquus* (Reeve, 1849).

#### Bulinus opalinus

Taxon classificationAnimaliaBasommatophoraPlanorbidae

Sowerby I, 1833

Figs 22iv–v , L15i

Bulinus opalinus Sowerby I 1833 in Sowerby I and II 1832–1841: 7, fig. 47; Sowerby I in Gray and Sowerby I 1839: 144, pl. 38 fig. 8.
Bulimus opalinus ; Pfeiffer 1848b: 107.
Leiostracus perlucidus ; Simone 2006: 123, fig. 384.

##### Type locality.

“Brazil”.

##### Label.

“Brazil”, taxon label in Pfeiffer’s handwriting. M.C. label style IV.

##### Dimensions.

Not given. Figured specimen H 27.8, D 14.3, W 7.4.

##### Type material.

NHMUK 1975442, three probable syntypes (Cuming coll.).

##### Remarks.

Sowerby did not state on how many specimens his description was based; he wrote “Nob.”, thus “ours”, meaning the author claimed his right as describer of the new taxon, not necessarily proof of presence in his own collection. As Breure and Ablett (2011: 10) suggested that these might have been swapped with Cuming, the three specimens found are treated as probable syntypes. They are accompanied by two labels in Pfeiffer’s handwriting; one “Bul. opalinus / Sow”, the other in different ink “perlucidus Spix”. In Pfeiffer 1848: 108 the dimensions “Long. 27, diam. 14 mill.” were given; this corresponds to the largest specimen in the lot. The citation in Richardson (1995: 207) to “Pfeiffer, Mono. Helic. Viv. 1: 231” refers to *Helix opalina* Sowerby I, 1841, and is thus in error. The current systematic position follows Simone (2006).

##### Current systematic position.

Simpulopsidae, *Leiostracus perlucidus* (Spix, 1827).

#### Bulimus ovulum

Taxon classificationAnimaliaStylommatophoraBulimulidae

Reeve, 1849

Figs 23v–vi , L15ii

Bulimus ovulum Reeve 1849 [1848–1850]: pl. 76 fig. 556; Breure 1979: 131. pl. 12 fig. 48.
Rhinus ovulum ; Breure 1978: 232 (lectotype designation); Simone 2006: 129, fig. 412.

##### Type locality.

“Philippine Islands; Cuming”.

##### Label.

“Pernambuco, Brazil”, taxon label in Pfeiffer’s handwriting. M.C. label style I, V.

##### Dimensions.

Not given. Figured specimen H 20.1, D 12.3, W 6.4.

##### Type material.

NHMUK 1975416, lectotype; 1975417, two paralectotypes (Cuming coll.).

##### Remarks.

Reeve did not state on how many specimens his description was based, but mentioned “A shell of rather solid growth...”; this is herein not considered as sufficient evidence that he had only one shell for his description. The material was in “Mus. Cuming”. Richardson (1995: 226) incorrectly classified this taxon with *Naesiotus* Albers, 1850. The current systematic position follows Simone (2006); the shell height given by him is erroneous.

##### Current systematic position.

Simpulopsidae, *Rhinus ovulum* (Reeve, 1849).

#### Helix phlogera

Taxon classificationAnimaliaStylommatophoraHelicidae

d’Orbigny, 1835

Figs 13iii–iv , L15iii

Helix phlogera d’Orbigny 1835: 8.
Bulimus phlogerus ; d’Orbigny 1837 [1834–1847]: 259, pl. 29 figs 6–8 [text 30 March 1838]; Gray 1854: 12.

##### Type locality.

“provincia Chiquitensi (republica Boliviana)”.

##### Label.

“Sn Xavier, Chquitos (Bolivia)”, in d’Orbigny’s handwriting.

##### Dimensions.

“Longit. 55 millim.; latit. 24 millim.”. Figured specimen H 59.8, D 26.8, W 6+.

##### Type material.

NHMUK 1854.12.4.86, six syntypes (d’Orbigny coll.).

##### Remarks.

d’Orbigny (1835) did not state on how many specimens his description was based; he said his taxon was identical to *Helix regina* var. β Férussac, 1821. In d’Orbigny (1838 [1834–1847]: 260) the locality was specified as “environs des Missions de San-Xavier et de Concepcion”; see Breure 1973. Of the material found, none of the shells corresponds exactly with d’Orbigny’s figure. The current systematic position is according to Richardson (1993: 108).

##### Current systematic position.

Orthalicidae, *Orthalicus phlogerus* (d’Orbigny, 1835).

#### Bulimus phoebus

Taxon classificationAnimaliaStylommatophoraBulimulidae

Pfeiffer, 1863

Figs 7iii–v , L15iv

Bulimus phoebus Pfeiffer 1863: 274; Pfeiffer 1868b: 9; Breure 1979: 30 (lectotype designation).
Plekocheilus phoebus ; Pilsbry 1895 [1895–1896]: 81.
Plekocheilus (Eurytus) phoebus ; Breure 1978: 15, pl. 11 fig. 6; Breure and Borrero 2008: 6.

##### Type locality.

“Ecuador”.

##### Label.

“Ecuador”, taxon label in Pfeiffer’s handwriting. M.C. label style IV.

##### Dimensions.

“Long. 31, diam. 15 mill.”. Figured specimen H 30.5, D 17.5, W 5.5.

##### Type material.

NHMUK 1975143, lectotype (Cuming coll.).

##### Remarks.

Pfeiffer did not state on how many specimens his description was based, but described his material “from the collection of H. Cuming”. This taxon has long been associated with Plekocheilus (Eurytus) Albers, 1850, but re-examination of the type—of which the protoconch proves to be smooth—plus recent collections in north-western Ecuador (Breure unpublished data) reveal that this taxon belongs to *Clathrorthalicus* Strebel, 1909. It may be closely allied to *Bulimus corydon* Crosse, 1869, *Bulimus magnificus* Pfeiffer, 1848 and *Bulimus victor* Pfeiffer, 1854; however, further anatomical and molecular studies should clarify the current systematic position.

##### Current systematic position.

Orthalicidae, *Clathrorthalicus phoebus* (Pfeiffer, 1863) (**comb. n.**).

#### Bulimus plumbeus

Taxon classificationAnimaliaStylommatophoraBulimulidae

Pfeiffer, 1855

Figs 6iv–vi , L16i

Bulimus plumbeus Pfeiffer 1855d: 114; Pfeiffer 1859: 369; Breure 1979: 44.
Strophocheilus plumbeus ; Pilsbry 1895 [1895–1896]: 49.
Thaumastus (Thaumastus) plumbeus ; Breure 1978: 31, pl. 11 fig. 1 (lectotype designation).

##### Type locality.

“Venezuela”.

##### Label.

“Venezuela”. M.C. label style III.

##### Dimensions.

“Long. 93, diam. 36 mill.”. Figured specimen H 93.0, D 40.5, W 5.9.

##### Type material.

NHMUK 1975130, lectotype; 1975131, one paralectotype (Cuming coll.).

##### Remarks.

Pfeiffer did not state on how many specimens his description was based, but described his material “from the collection of H. Cuming”. Although there is no label in Pfeiffer’s handwriting, the type status of these specimens is not disputed as the shell height matches the original data. This taxon has been considered a junior subjective synonym of Helix (Cochlogena) pardalis Férussac, 1821 (Richardson 1995: 202), but re-examination of the type leads us to tentatively retain the classification of Breure (1978). It may be noted that the locality of this taxon is well outside the range of *Thaumastus*; however, it could possibly occur in southwestern Venezuela. Once located, further anatomical and molecular studies should shed more light on its systematic position.

##### Current systematic position.

Megaspiridae, *Thaumastus plumbeus* (Pfeiffer, 1855).

#### Bulimus porphyrius

Taxon classificationAnimaliaStylommatophoraBulimulidae

Pfeiffer, 1847

Figs 11i–iv , L16iii

Bulimus porphyrius Pfeiffer 1847: 114; Reeve 1848 [1848–1850]: pl. 15 fig. 89; Pfeiffer 1848b: 199; Breure 1979: 41.
Thaumastus (Scholvienia) porphyrius ; Breure 1978: 46 (lectotype designation).

##### Type locality.

“Bolivia (T. Bridges)”.

##### Label.

“Bolivia”, “andes of Caxamarca / Peru”, taxon label in Pfeiffer’s handwriting. M.C. label style IV, V.

##### Dimensions.

“Long. 51, diam. 20 mill.”. Figured specimen H 51.5, D 22.0, W 6.6.

##### Type material.

NHMUK 1975277, lectotype; 1975278, two paralectotypes (Cuming coll.).

##### Remarks.

Pfeiffer did not state on how many specimens his description was based; it was, however, one of the taxa from the Cuming collection. Breure (1978) has discussed the localities and suggested that both labels are probably erroneous. This taxon has hitherto been classified with Thaumastus (Scholvienia) Strebel, 1910. Given the results of Breure and Romero (2012), who found that subgenera of *Thaumastus* belong to different families, the familiar association of this taxon is tentatively made to the Orthalicidae, and *Scholvienia* is provisionally given generic status. Further anatomical and molecular studies should shed more light on its systematic position.

##### Current systematic position.

?Orthalicidae, *Scholvienia porphyria* (Pfeiffer, 1847) (**comb. n.**).

#### Bulimus requieni

Taxon classificationAnimaliaStylommatophoraBulimulidae

Pfeiffer, 1853

Figs 29iii–iv , L16ii

Bulimus requieni Pfeiffer 1853b: 389; Pfeiffer 1854b: 137; Pfeiffer 1855 in Küster and Pfeiffer 1840–1865: 248, pl. 66 fig. 8; Breure 1979: 44.
Strophocheilus requieni ; Pilsbry 1895 [1895–1896]: 55, pl. 27 fig. 94.
Thaumastus (Thaumastus) requieni ; Breure 1978: 31 (lectotype designation).
Thaumastus requieni ; Simone 2006: 154, fig. 523.

##### Type locality.

“Brasilia”.

##### Label.

“Brazils”, taxon label in Pfeiffer’s handwriting. M.C. label style IV.

##### Dimensions.

“Long. 62, diam. 26 mill.”. Figured specimen H 62.0, D 29.0, W 5.3+.

##### Type material.

NHMUK 1975301, lectotype; 1975302, one paralectotype (Cuming coll.).

##### Remarks.

Pfeiffer did not state on how many specimens his description was based, but described his material from “Mus. Cuming”. The top of the lectotype is slightly damaged. The protoconch is sculptured with slightly waving axial riblets. Both specimens appear to be subadult; further studies are needed to ascertain the taxonomic position of this taxon. The current systematic position follows Simone (2006).

##### Current systematic position.

Megaspiridae, *Thaumastus requieni* (Pfeiffer, 1853).

#### Vitrina salomonia

Taxon classificationAnimaliaStylommatophoraVitrinidae

Pfeiffer, 1853

Figs 26i–iii , L16iv

Vitrina salomonia Pfeiffer 1853a: 51; Pfeiffer 1853b: 623; Pfeiffer 1854a: 60.
Simpulopsis salomonia ; Pfeiffer 1854 in Küster and Pfeiffer 1840–1865: 29, pl. 6 figs 17–19; Reeve 1862: pl. 2 fig. 8.
Simpulopsis (?) salomonia ; Pilsbry 1899: 226, pl. 63 figs 76–78.
Simpulopsis (Simpulopsis) rufovirens (Moricand); Breure 1978: 232 (lectotype designation).

##### Type locality.

“in insulis Salomonis”.

##### Label.

“Solomons Isl”, taxon label in Pfeiffer’s handwriting. M.C. label style III.

##### Dimensions.

“Long. 11, diam. 9 mill.”. Figured specimen H 11.1, D 10.7, W 4.5.

##### Type material.

NHMUK 1975485, lectotype (Cuming coll.).

##### Remarks.

Pfeiffer did not state on how many specimens his description was based, but noted “Mus. Cuming”. He also remarked “Diese Art ist mit der brasilianischen Gruppe Simpulopsis Beck sehr nahe verwandt”, which might have led Pilsbry (1899: 226) to suggest that the locality given by Pfeiffer was erroneous. Breure (1978) suggested that this taxon might be a junior subjective synonym of Helix (Succinea) rufovirens Moricand, 1846. Richardson (1995: 367) considered this taxon as a separate species; Simone (2006) did not mention it at all. Tentatively the classification of this taxon by Breure (1978) is herein retained, until a further revision of this group clarifies its taxonomic status.

##### Current systematic position.

Simpulopsidae, Simpulopsis (Simpulopsis) rufovirens (Moricand, 1846).

#### Bulimus salteri

Taxon classificationAnimaliaStylommatophoraBulimulidae

Sowerby III, 1890

Figs 13i–ii , L17i

Bulimus salteri Sowerby III 1890: 578, pl. 50 fig. 4; Breure 1979: 45.

##### Type locality.

“Catamarca, Andes Peruviae”.

##### Label.

“Andes of Peru”; printed label.

##### Dimensions.

“Long. 70, maj. diam. 35 mill.”. Figured specimen H 69.9, D 35.2, W 6.0.

##### Type material.

NHMUK 1907.11.21.118, lectotype (da Costa coll.).

##### Remarks.

Sowerby also described a (larger) “var. γ”, and remarked “[t]he two shells form part of the collection of Mr. S.J. Da Costa, and there is a specimen of each variety [typical one and var. γ] in the National Collection at South Kensington [= NHMUK]”. The original series thus seems to have comprised two specimens, and the reference in Breure (1979) to “HT BMNH 1907.11.21.118” has to be interpreted as lectotype designation under Art. 74.6 ICZN, also following Recommendation 73F. We have, however, not been able to locate a varietal form of this taxon in the da Costa collection within the NHMUK. In the General collection we found two specimens. One is labeled “*Thaumastus salteri* / Andes of Peru / Purch Sowerby”, and is registered as NHMUK 1883.10.24.8 (it is listed in the register as *Orthalicus* and no specific name). The second specimen is labelled “*salteri* var. / Peru / Mus. Cuming”; this is the only specimen with a varietal label, but nonetheless dubious if it belonged to the original series and Sowerby’s varietal shell may have been lost from the collection. This taxon has hitherto been classified with Thaumastus (Quechua) Strebel, 1910. Given the results of Breure and Romero (2012), who found that subgenera of *Thaumastus* belong to different families, the familial association of this taxon is tentatively made to the Orthalicidae, and *Quechua* is provisionally given generic status, pending further anatomical and molecular studies.

##### Current systematic position.

?Orthalicidae, *Quechua salteri* (Sowerby III, 1890) (**comb. n.**).

#### Bulimus sarcochilus

Taxon classificationAnimaliaStylommatophoraBulimulidae

Pfeiffer, 1857

Figs 22vi–viii , L16v

Bulimus sarcochilus Pfeiffer 1857e: 157; Pfeiffer 1859: 412; Breure 1979: 127.
Bulimulus sarcochilus ; Pilsbry 1897 [1897–1898]: 80.
Leiostracus (Leiostracus) sarcochilus ; Breure 1978: 227, figs 396–397 (lectotype designation).
Leiostracus sarcochilus ; Simone 2006: 123, fig. 386.

##### Type locality.

“in Brasilia septentrionali (Miers)”.

##### Label.

“North of Brazils / F. Miers Esq”, taxon label in Pfeiffer’s handwriting. M.C. label style I.

##### Dimensions.

“Long. 21–25, diam. 10–11 1/2 mill.”. Figured specimen H 24.7, D 13.1, W 7.7.

##### Type material.

NHMUK 1975398, lectotype; 1975399, one paralectotype (Cuming coll.).

##### Remarks.

Pfeiffer did not state on how many specimens his description was based, but from his dimensions it is clear that he had more than one specimen at hand. From Pfeiffer (1859) it becomes clear that this was one of the taxa described from “Mus. Cuming”. Breure (1978) re-described the species on the basis of additional material and established the first exact locality in state Espírito Santo. The current systematic position follows Simone (2006), whose reference to the figured type as “syntype” is erroneous.

##### Current systematic position.

Simpulopsidae, *Leiostracus sarcochilus* (Pfeiffer, 1857).

#### Bulimus saturnus

Taxon classificationAnimaliaStylommatophoraBulimulidae

Pfeiffer, 1860

Figs 15iii–v , L17v

Bulimus saturanus Pfeiffer 1860: 136.
Bulimus satuanus Pfeiffer 1860: pl. 51 fig. 6.
Bulimus saturnus ; Pfeiffer 1861a: 11; Pfeiffer 1868b: 14.
Porphyrobaphe saturnus ; Pilsbry 1899: 154, pl. 50 fig. 25.

##### Type locality.

“Pallatanga, Republic of Ecuador (*Mr. Fraser*)”.

##### Label.

“Pallatango Republic of Ecuador M^r^ Fraser”, taxon label in Pfeiffer’s handwriting. M.C. label style I.

##### Dimensions.

“Long. 76, diam. 33 mill.”. Figured specimen H 75.8, D 38.4, W 6.7.

##### Type material.

NHMUK 20140080, three syntypes, Fraser leg. (Cuming coll.).

##### Remarks.

Pfeiffer did not state on how many specimens his description was based. In the original paper he made twice an error in the name, which was corrected in Pfeiffer (1861a; without explicit comment), and Pfeiffer (1868; “sphalm. *Saturanus*”); this is treated as a *lapsus calami* under Art. 32.1 jo. 24.2.4 ICZN. Breure and Borrero (2008: 28) have pointed out that “Pallatanga” could not be assigned unequivocally to a locality, as it is found twice in modern gazetteers.

##### Current systematic position.

Orthalicidae, Porphyrobaphe (Porphyrobaphe) saturnus (Pfeiffer, 1860).

#### Bulimus simulus

Taxon classificationAnimaliaStylommatophoraBulimulidae

Morelet, 1851

Figs 26iv–vi , L17ii

Bulimus simulus Morelet 1851: 11; Pfeiffer 1853d: 383; Breure 1979: 134.

##### Type locality.

[Guatemala] “sylvas Petenensis”.

##### Label.

“forêt de Dolores”, taxon label in Morelet’s handwriting.

##### Dimensions.

“Longit. 11 – Diam. 9 [mm]”. Figured specimen H 8.26, D 6.79, W 4.3.

##### Type material.

NHMUK 1893.2.4.1128–1129, two syntypes (Morelet coll.).

##### Remarks.

Morelet did not state on how many specimens his description was based. On the board on which the labels are glued has been written in a later hand “Type largest / Test. Noviss. No. 101”. The locality on the label probably refers to the village of Dolores, Petén, Guatemala, which is thus the exact type locality. The current systematic position follows Thompson (2011: 130).

##### Current systematic position.

Simpulopsidae, Simpulopsis (Simpulopsis) simula (Morelet, 1851).

#### Strophocheilus (Eurytus) subirroratus

Taxon classificationAnimaliaStylommatophoraStrophocheilidae

da Costa, 1898

Figs 16i–iv , L17iv

Strophocheilus (Eurytus) subirroratus da Costa 1898: 83, fig. II; Breure and Schouten 1985: 54.
Porphyrobaphe subirroratus ; Pilsbry 1901 [1901–1902]: 163, pl. 24 fig. 11.
Porphyrobaphe (Oxyorthalicus) subirroratus ; Breure and Borrero 2008: 29.

##### Type locality.

“Paramba, Ecuador”.

##### Label.

“Paramba, Ecuador”, in da Costa’s handwriting.

##### Dimensions.

“Long. 63, diam. 33 mm.”. Figured specimen H 62.6, D 36.6, W 5.9.

##### Type material.

NHMUK 1907.11.21.114, lectotype (da Costa coll.).

##### Remarks.

da Costa did not state on how many specimens his description was based; the reference in Breure and Schouten (1985) to “HT BMNH 1907.11.21.114” has to be interpreted as lectotype designation under Art. 74.6 ICZN. The current systematic position follows Breure and Borrero (2008). However, it should be noted that Strebel (1909: 120)—after establishing the subgenus *Oxyorthalicus*—wrote “Die Skulpturbeschreibung [by da Costa] bezw. das Fehlen der erhabenen Streifen scheint mir für die Untergattung unwahrscheinlich”. Further anatomical and molecular research should thus shed more light on the taxonomic position.

##### Current systematic position.

Orthalicidae, Porphyrobaphe (Oxyorthalicus) subirroratus (da Costa, 1898).

#### Drymaeus (Leiostracus) onager subtuszonata

Taxon classificationAnimaliaStylommatophoraBulimulidae

Pilsbry, 1899

Figs 23i–ii , L19iii

Bulimus onager Reeve 1848 [1848–1850]: pl. 45 fig. 284. Not *Bulimulus onager* Beck, 1837.
Drymaeus (Leiostracus) onager var. subtuszonata Pilsbry 1899: 95, pl. 14 fig. 17.
Leiostracus subtuzonatus [sic]; Simone 2006: 123, fig. 387B.

##### Type locality.

Not given.

##### Label.

Not given [“Brazil” added in a later hand]. M.C. label style I, V.

##### Dimensions.

“[L]ength of 28 mm”. Figured specimen H 29.0, D 14.8, W 7.9.

##### Type material.

NHMUK 20130094, three probable paralectotypes (Cuming coll.).

##### Remarks.

Pilsbry did not state on how many specimens his description was based, and gave no type locality as he described what he regarded as a colour variation only. His figure was a black and white copy of Reeve’s figure. Salvador and Cavallari (2013) have given this variety specific status and designated a specimen from MZSP as neotype. In doing so they disregarded material in the NHMUK (Reeve) and ANSP (Pilsbry), and their designation did not fulfil the requirements of Art. 75 ICZN. Salvador et al. (2014) corrected this issue and selected the figure of Reeve 1848 [1848–1850]: pl. 45 fig. 284 as lectotype, in accordance with Recommendation 74B ICZN. The specimens found are accompanied by a Reeve label, but cannot be matched exactly to his figure; they are considered as probable paralectotypes.

##### Current systematic position.

Simpulopsidae, *Leiostracus subtuszonatus* (Pilsbry, 1899).

#### Bulimus thompsonii

Taxon classificationAnimaliaStylommatophoraBulimulidae

Pfeiffer, 1845

Figs 8i–ii , L17iii

Bulimus thompsonii Pfeiffer 1845: 74; Pfeiffer 1848b: 141; Reeve 1848 [1848–1850]: pl. 24 fig. 158; Breure 1979: 40.
Thaumastus (Kara) thompsoni [sic]; Breure 1978: 34 (lectotype designation); Breure and Borrero 2008: 7.

##### Type locality.

[Ecuador] “Quito. (Coll. Cuming)”.

##### Label.

“Quito”, taxon label in Pfeiffer’s handwriting. M.C. label style IV, V.

##### Dimensions.

“Long. 70, diam. 31 mill.”. Figured specimen H 71.0, D 32.0, W 6.2.

##### Type material.

NHMUK 1975464, lectotype; 1975465, two paralectotypes (Cuming coll.).

##### Remarks.

Pfeiffer did not state on how many specimens his description was based. The specimen figured by Reeve has been selected lectotype by Breure (1978); the paralectotypes are less slender. On the basis of molecular analyses of Breure and Romero (2012), the genus *Kara* Strebel, 1910 has been placed in the family Orthalicidae (Breure 2011).

##### Current systematic position.

Orthalicidae, *Kara thompsonii* (Pfeiffer, 1845).

#### Porphyrobaphe vicaria

Taxon classificationAnimaliaStylommatophoraOrthalicidae

Fulton, 1896

Figs 20i–ii , L18i

Bulimus labeo Reeve 1848 [1848–1850]: pl. 71 fig. 207b, pl. 72 fig. 207c. Not *Bulimus labeo* Broderip, 1828.
Porphyrobaphe vicaria Fulton 1896: 103.

##### Type locality.

“Leimabamba, Peru, 8000 feet (*O.T. Baron*)”.

##### Label.

“Limabambo Peru”. M.C. label style IV.

##### Dimensions.

Not given. Figured specimen H 82.2, D 46.7, W 6.3+.

##### Type material.

NHMUK 20100507, holotype, Lobb leg. (Cuming coll.).

##### Remarks.

Fulton mentioned “[t]ype in British Museum (Cuming Collection)”, and said his taxon had been figured by Reeve. Reeve wrote: “It is with much gratification that I am enabled to give an original figure of the *Bulimus labeo*, figured at. Pl. XXXV, from a figure in the Zoological Journal. This shell, from the Cumingian collection, which I take to be identical with the lost specimen [see Pain 1959] (...). It was collected by Mr. Lobb at Limabamba, Peru; a district seldom visited by travellers, and the same in which Lieut. Mawe obtained the original specimen.” From this text it may be concluded that Reeve had only seen one specimen, identical to the lost type of Broderip; Reeve’s shell is thus the holotype of Fulton’s taxon. It is also clear that the altitude and collector data given by Fulton are erroneous. The current systematic position at species level follows Richardson (1993: 128).

##### Current systematic position.

Orthalicidae, Sultana (Metorthalicus) yatesi (Pfeiffer, 1855).

#### Bulimus victor

Taxon classificationAnimaliaStylommatophoraBulimulidae

Pfeiffer, 1854

Figs 7vi–vii , L18iii

Bulimus victor Pfeiffer 1854d: 128; Pfeiffer 1859: 368; Pfeiffer 1861 [1860–1866]: 169, pl. 46 figs 1–2; Breure and Schouten 1985: 55 (lectotype designation).
Plekocheilus victor ; Pilsbry 1895 [1895–1896]: 82, pl. 33 figs 47–48; Linares and Vera 2012: 174.

##### Type locality.

“in provincia Antioquia, Columbiae (*Schlim*)”.

##### Label.

“Province of Antioquia / [...] Schlim [...]”, taxon label in Pfeiffer’s handwriting. M.C. label style III.

##### Dimensions.

“Long. 65, diam. 29 mill.”. Figured specimen H 64.0, D 36.7, W 5+.

##### Type material.

NHMUK 1975242, lectotype; 20100567, one paralectotype (Cuming coll.).

##### Remarks.

Pfeiffer did not state on how many specimens his description was based; besides the specimen corresponding to Pfeiffer’s dimensions, and selected lectotype by Breure and Schouten (1985), a second specimen was found designated as “var.” by Pfeiffer (1861: 169). The label accompanying the lectotype is partly fading away; the apex of this specimen is missing. This species has been listed by Richardson (1993: 120) under *Porphyrobaphe* Shuttleworth, 1856, and also under *Plekocheilus* Guilding, 1828 (Richardson 1995: 324). The reference of Linares and Vera (2012) for this species from Putumayo must be viewed with suspicion until the voucher specimen has been studied, as there may be a confusion with a local *Plekocheilus* species. This taxon has long been associated with Plekocheilus (Eurytus) Albers, 1850, but re-examination of the type material—the protoconch of the paralectotype proves to be smooth—plus recent collections in north-western Ecuador (Breure unpublished data) reveal that this taxon belongs to *Clathrorthalicus* Strebel, 1909. It may be closely allied to *Bulimus corydon* Crosse, 1869, *Bulimus magnificus* Pfeiffer, 1848, and *Bulimus phoebus* Pfeiffer, 1863; however, further anatomical and molecular studies should reveal the correct systematic position.

##### Current systematic position.

Orthalicidae, *Clathrorthalicus victor* (Pfeiffer, 1854) (**comb. n.**).

#### Simpulosis vincentina

Taxon classificationAnimaliaStylommatophoraSimpulopsidae

E.A. Smith, 1895

Figs 29i–ii , L19i

Simpulosis vincentina E.A. Smith 1895: 305, pl. 21 figs 4–5; Pilsbry 1899: 219, pl. 63 figs 65–66; Breure 1979: 134.

##### Type locality.

[West Indies, St. Vincent] “Damp forest, Upper Richmond valley, 2000 ft, on leaves of *Artanthe* (Piperacea) (H.H. Smith)”.

##### Label.

“Damp forest, Upper Richmond valley, 2000 ft, on leaves of Artanthe (Piperacea), St. Vincent, B.W.I.”.

##### Dimensions.

“Longit. 13, diam. maj. 10 mm”. Figured specimen H 11.4, D 10.2, W 2.8.

##### Type material.

NHMUK 1895.6.17.458, holotype, H.H. Smith leg.

##### Remarks.

E.A. Smith wrote “[o]nly a single specimen was collected.” Both body of the last whorl and the lip are partly broken in the holotype. The current systematic position follows Richardson (1995: 368).

##### Current systematic position.

Simpulopsidae, Simpulosis (Simpulopsis) vincentina E.A. Smith, 1895.

#### Bulimus yanamensis

Taxon classificationAnimaliaStylommatophoraBulimulidae

Morelet, 1863

Figs 8v–vi , L18ii

Bulimus yanamensis Morelet 1863: 171, pl. 8 fig. 3; Pilsbry 1868: 87; Breure 1979: 40.
Strophocheilus yanamensis ; Pilsbry 1895 [1895–1896]: 54, pl. 27 fig. 97.
Thaumastus (Kara) yanamensis ; Breure 1978: 34 (lectotype designation).

##### Type locality.

[Peru] “Yanama”.

##### Label.

“Yanama. Pérou”, taxon label in Morelet’s handwriting.

##### Dimensions.

“Longit. 58; diam. 25 (...) mill.”. Figured specimen H 48.6, D 26.9, W 5.4.

##### Type material.

NHMUK 1893.2.4.167–168, [two paralectotypes] (Morelet coll.).

##### Remarks.

Morelet did not state on how many specimens his description was based; the two specimens mentioned by Breure (1978) were absent, although the labels of the lot have been found and a picture has been taken. The lectotype is present in the MNHG collection. This taxon has been associated with the genus *Kara* Strebel, 1910. On the basis of molecular analyses of Breure and Romero (2012), this genus has been placed in the family Orthalicidae (Breure 2011).

##### Current systematic position.

Orthalicidae, *Kara yanamensis* (Morelet, 1863).

#### Bulimus yatesi

Taxon classificationAnimaliaStylommatophoraBulimulidae

Pfeiffer, 1855

Figs 20iii–iv , L19ii

Bulimus yatesi Pfeiffer 1855c: 93, pl. 31 fig. 5; Pfeiffer 1856 [1854–1860]: 63, pl. 18 figs 1–2; Pfeiffer 1859: 371.
Orthalicus yatesi ; Pilsbry 1899: 202, pl. 43 fig. 17.
Sultana (Metorthalicus) yatesi ; Breure and Schouten 1985: 28 (lectotype designation).

##### Type locality.

“Meobamba, Eastern Peru (*Mr. Yates*)”.

##### Label.

“Meobamba, East Peru”. M.C. label style III.

##### Dimensions.

“Long. 82, diam. 32 mill.”. Figured specimen H 84.3, D 39.7, W 7.2.

##### Type material.

NHMUK 1975239/1, lectotype; 1975239/2–3, two paralectotypes (Cuming coll.).

##### Remarks.

Pfeiffer did not state on how many specimens his description was based; Pfeiffer (1856 [1854–1860]) mentioned “Aus H. Cumings’s und meiner Sammlung”. The specimen figured by Pfeiffer (1855c) was selected lectotype by Breure and Schouten (1985). The current systematic position follows Richardson (1993: 127) at the species level.

##### Current systematic position.

Orthalicidae, Sultana (Metorthalicus) yatesi (Pfeiffer, 1855).

### Addenda et corrigenda

#### I. Nomen inquirendum

The systematic position of the following taxon cannot be ascertained at present, and it is herein considered a *nomen inquirendum*.

##### Bulimulus (Protoglyptus) dejectus

Taxon classificationAnimaliaStylommatophoraOrthalicidae

Fulton, 1907

Figs 9iii , L6i

Bulimulus (Protoglyptus) dejectus Fulton 1907: 153, pl. 10 fig. 1; Breure 2011: 22, fig. 15B, 15iii.
Protoglyptus dejectus ; Simone 2006: 148, fig. 500A.

###### Type locality.

“Santa Catarina (*fide* Linnaea Institute label)”.

###### Label.

“St. Catharina”.

###### Dimensions.

“Maj. diam. 10, alt. 29 mm”. Figured specimen H 29.2, D 10.0, W 7.8.

###### Type material.

NHMUK 1907.5.3.163, lectotype (ex Sowerby and Fulton).

###### Remarks.

Fulton did not state on how many specimens his description was based. The specimen found agrees with Fulton’s measurements and is now designated lectotype (**design. n.**). The sculpture of the protoconch is not with axial wrinkles as usual in *Protoglyptus* Pilsbry, 1897, but with axial wrinkles, partly broken into granules. It may be noted that all species currently classified with this genus occur in the West Indies (Breure and Romero 2012; Breure and Ablett 2014). Breure (2011) retained this taxon with this genus, but expressed doubts and suggested further research. The surface of the teleoconch has spiral series of small granules, denoting an epidermis covered with hairs when fresh; this has both been observed in some species of *Rhinus* Albers, 1860, and *Naesiotus* Albers, 1850. Although the shape of the shell cannot be conclusive evidence for generic classification, it may be noted that Fulton compared this species to *Helix crepundia* d’Orbigny, 1835, which has been classified with *Naesiotus* *sensu lato* (Breure and Ablett 2014). Only further anatomical and molecular work can shed more light on the correct systematic position of this taxon.

###### Current systematic position.

?Bulimulidae, ?*Naesiotus dejectus* (Fulton, 1907). *Nomen inquirendum*.

#### II. Types not located.

Type material of the following taxa, previously known to be extant in the NHMUK, has not been found during our study.

##### Bulimus dennisoni

Taxon classificationAnimaliaStylommatophoraBulimulidae

Reeve, 1848

Bulimus dennisoni Reeve 1848 [1848–1850]: pl. 26 fig. 166; Pfeiffer 1853b: 380; Pfeiffer 1855 in Küster and Pfeiffer 1840–1865: 245, pl. 66 figs 1–2.
Hemibulimus (Myiorthalicus) dennisoni ; Breure and Schouten 1985: 46.

###### Type locality.

“—?”.

###### Dimensions.

Not given.

###### Remarks.

The two syntypes mentioned by Breure and Schouten (1985) could not be located during our research. The size of these specimens falls within the variation mentioned by Pfeiffer (1853d: “71–83 mill.”) for material from Cuming’s and Dennison’s collection.

##### Helix miliola

Taxon classificationAnimaliaStylommatophoraHelicidae

d’Orbigny, 1835

Fig. L14i

Helix miliola d’Orbigny 1835: 17.
Pupa miliola d’Orbigny 1838 [1834–1847]: 323; Gray 1854: 24.

###### Type locality.

“imperio Brasiliano”.

###### Dimensions.

“Latit. 2 millim., longit. 1 millim.”.

###### Type material.

NHMUK 1854.12.4.239, [seven syntypes] (d’Orbigny coll.).

###### Remarks.

Seven specimens were known to be present (cf. the registration book, which has an undated note in pencil “6 missing”), but none could not be found. This taxon has been mentioned under two different species by Richardson (1993: 36, as synonym of *Bulinus janeirensis* Sowerby I, 1833; 1993: 47, as synonym of *Odontostomus juvencus* Mörch, 1852). The former is an erroneously reference to d’Orbigny 1837 [1834–1847]: pl. 39 figs 1–2, who corrected the legend to his figure to *Bulimus fuscagula* d’Orbigny “(figuré sous le faux nom de *Bulimus Miliola*)”; d’Orbigny 1846 [1834–1847]: 696.

##### Helix progastor

Taxon classificationAnimaliaStylommatophoraHelicidae

d’Orbigny, 1835

Fig. L15v

Helix progastor d’Orbigny 1835: 2; d’Orbigny 1836 [1834–1847]: 255, pl. 22 figs 12–15 [text 30 March 1838]; Gray 1854: 12.
Simpulopsis progastor ; Pilsbry 1899: 223, pl. 64 figs 1–3.
Eudioptus progastor ; Simone 2006: 180, fig. 655.

###### Type locality.

“Brasilianis oris”.

###### Dimensions.

“Longit. 7 millim.”.

###### Type material.

NHMUK 1854.12.4.72, [one syntype] (d’Orbigny coll.).

###### Remarks.

d’Orbigny (1838 [1834–1847]: 255) specified the type locality as “la province des Mines” [Minas Gerais]. This taxon was marked in Gray (1854) with “B.M.” [NHMUK], but the type material has not been located during our research.

##### Bulimus vitrinoides

Taxon classificationAnimaliaStylommatophoraBulimulidae

Reeve, 1848

Bulimus vitrinoides Reeve 1848 [1848–1850]: pl. 46 fig. 290; Breure 79: 136.

###### Type locality.

“—?” “Mus. Cuming”.

###### Dimensions.

Not given.

###### Remarks.

The syntype material mentioned by Breure (1979) has not been located during our study. It is possible that this material has not been registered. However, the NHMUK copy of Reeve (1848–1850) for *Bulimus vitrinoides* has the species name crossed out and ‘citrino-vitreus Moricand’ penciled in. In the general collection one lot was found (registered NHMUK 1841.4.28.110); one specimen matches the illustration but is smaller. These specimens are not considered type material as they are not from the Cuming collection but were ‘purchased of M. M. Parreys d’Vienna’.

#### III. Types not found in NHMUK, but expected to be present.

The following taxa were expected to be represented with type material; however, no material could be found matching the data in the original publication.

*Bulinus adamsonii*J.E. Gray 1834: 123.—Described from “the collection of Mr. Adamson in Newcastle”, of which the fate is unknown.

*Bulini guadaloupensis alba* Sowerby I in J.E. Gray and Sowerby I 1839: 144, pl. 38 fig. 13.

*Achatina atramentaria*Pfeiffer 1855d: 116.—Described from “the collection of H. Cuming”, but not found.

*Bulimus aulacostylus*Pfeiffer 1853b: 316.—Described from “Mus. Cuming”, but not found.

*Bulinus bilabiatus*Broderip and Sowerby I 1829: 49, suppl. pl. 40 figs 1–2.—The specimens figured by Reeve 1849 [1848–1850]: pl. 83 figs 201a–b are present in the General Collection (NHMUK 20110080).

*Bulinus bilineatus*Sowerby I 1833: 37.—Described from “shells collected by Mr. Cuming”, but not found.

*Bulinus bivittatus*Sowerby I 1833 [Sowerby I and II 1832–1841]: 7, fig. 46.

*Bulimus blainvilleanus*Pfeiffer 1848a: 230.—Described from “the collection of H. Cuming”, but not found.

*Bulimus bolivianus*Reeve 1848 [1848–1850]: pl. 44 fig. 281.—Described from “Mus. Denisson”.

**Figure 2.**
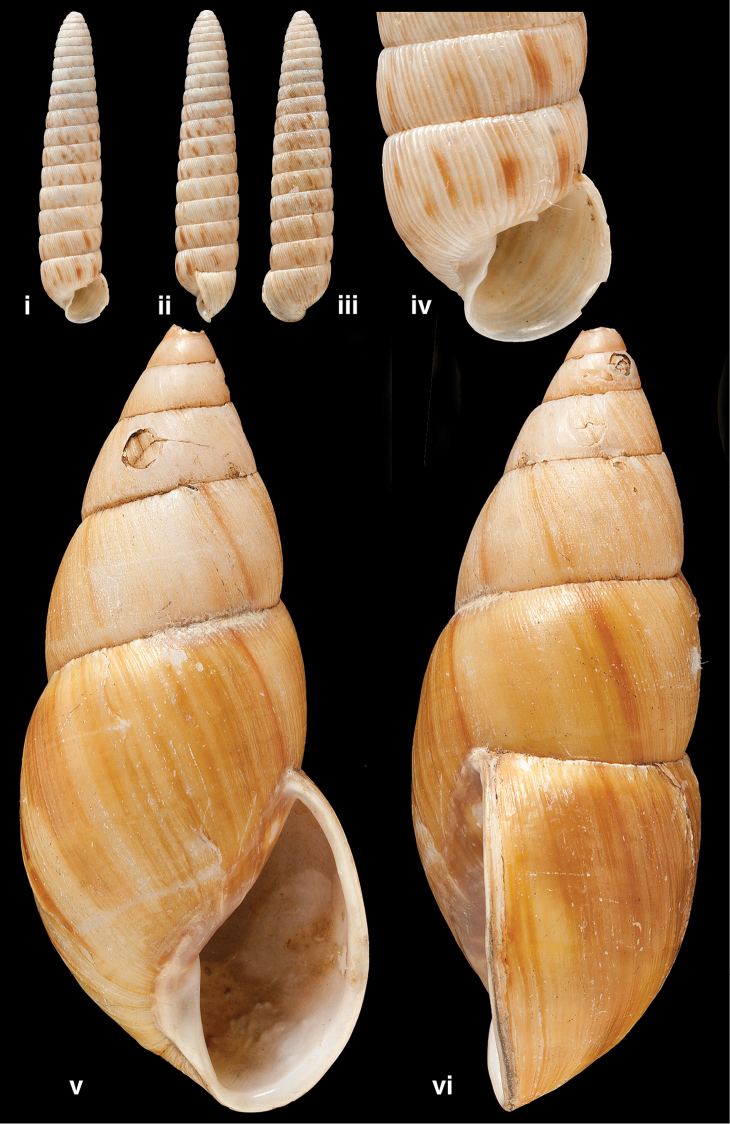
**i–iv** *Megaspira elata* (Gould, 1847), paratype NHMUK 1987060 (H = 37.2). *Thaumastus* species. **v–vi** *Thaumastus ascendens* (Pfeiffer, 1853), lectotype NHMUK 1975274 (H = 92.0).

**Figure 5.**
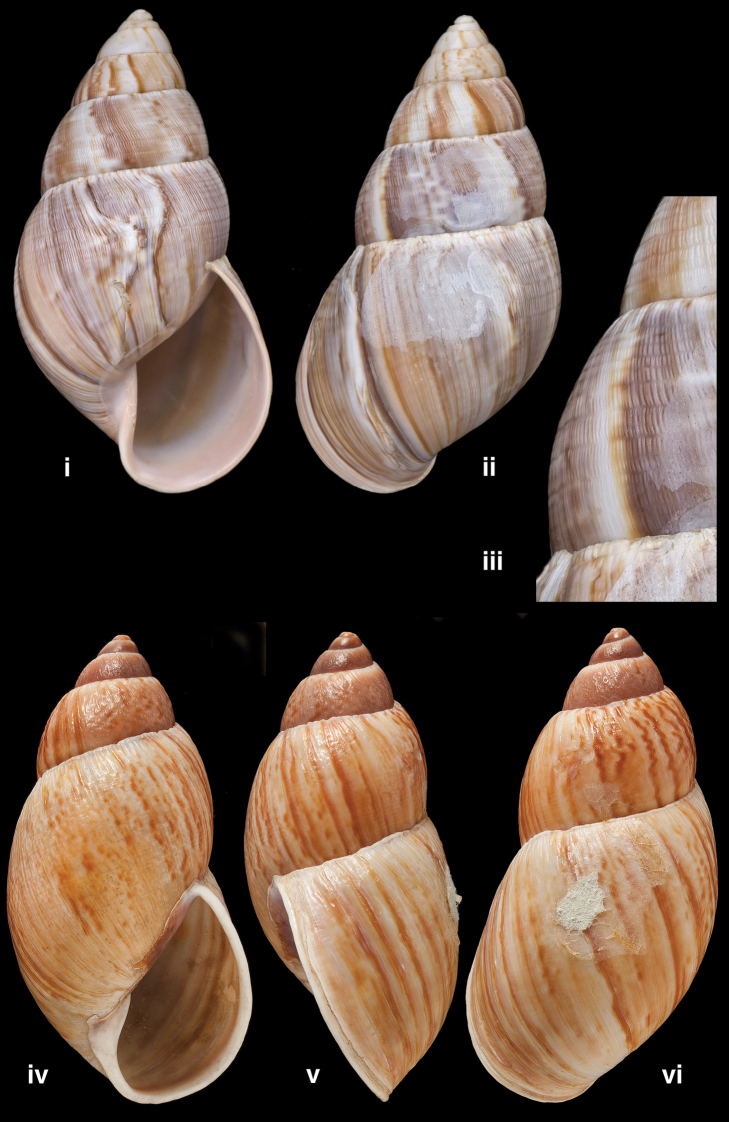
*Thaumastus* species. **i–iii** *Thaumastus integer* (Pfeiffer, 1855), lectotype NHMUK 1975244 (H = 81.5) **iv–vi** *Thaumastus loxostomus* (Pfeiffer, 1853), syntype NHMUK 1975125 (H = 71.3).

**Figure 7.**
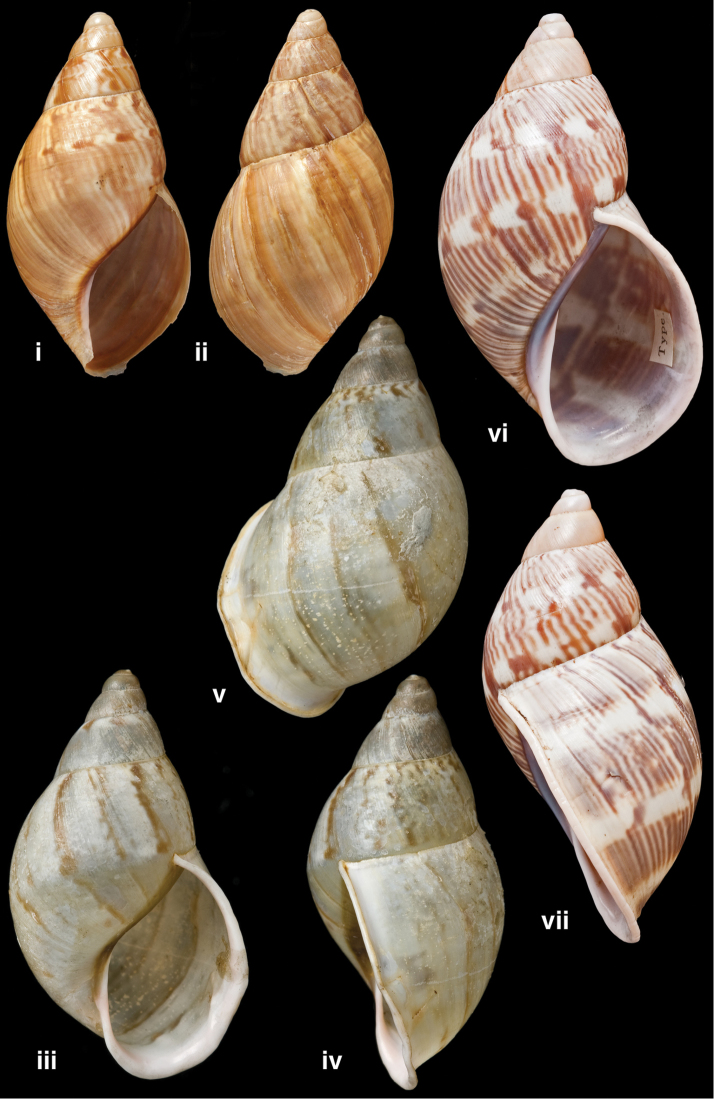
*Clathrorthalicus* species. **i–ii** *Clathrorthalicus magnificus* (Pfeiffer, 1848), syntype NHMUK 20100508 (H = 46.6) **iii–v** *Clathrorthalicus phoebus* (Pfeiffer, 1863), lectotype NHMUK 1975143 (H = 30.5) **vi–vii** *Clathrorthalicus victor* (Pfeiffer, 1854), lectotype NHMUK 1975242 (H = 64.0).

**Figure 11.**
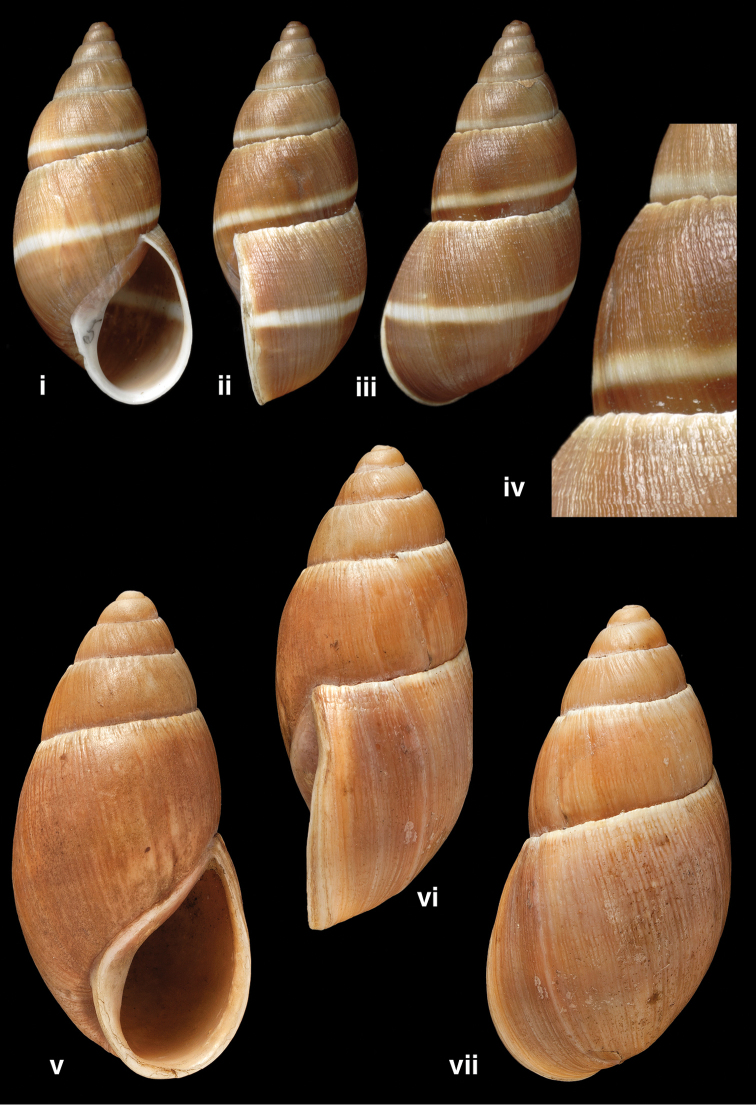
**i–iv** *Scholvienia porphyrius* (Pfeiffer, 1847), lectotype NHMUK 1975277 (H = 51.5) **v–vii** *Orthalicus bensoni* (Reeve, 1849), possible syntype NHMUK 1975582 (H = 66.6).

**Figure 13.**
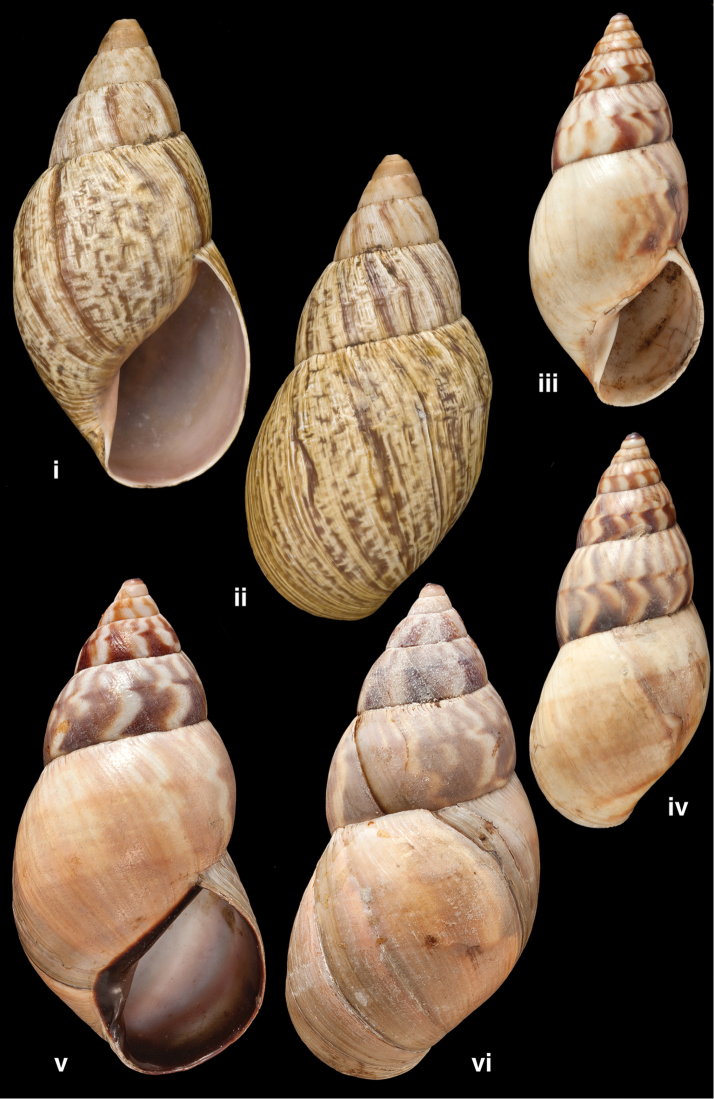
**i–ii** *Quechua salteri* (Sowerby III, 1890), lectotype NHMUK 1907.11.21.118 (H = 69.9) **iii–vi** *Orthalicus* species. **iii–iv** *Orthalicus phlogerus* (d’Orbigny, 1835), syntype NHMUK 1854.12.4.86 (H = 59.8) **v–vi** *Orthalicus mars* (Pfeiffer, 1861), syntype NHMUK 20100504 (H = 76.6).

**Figure 14.**
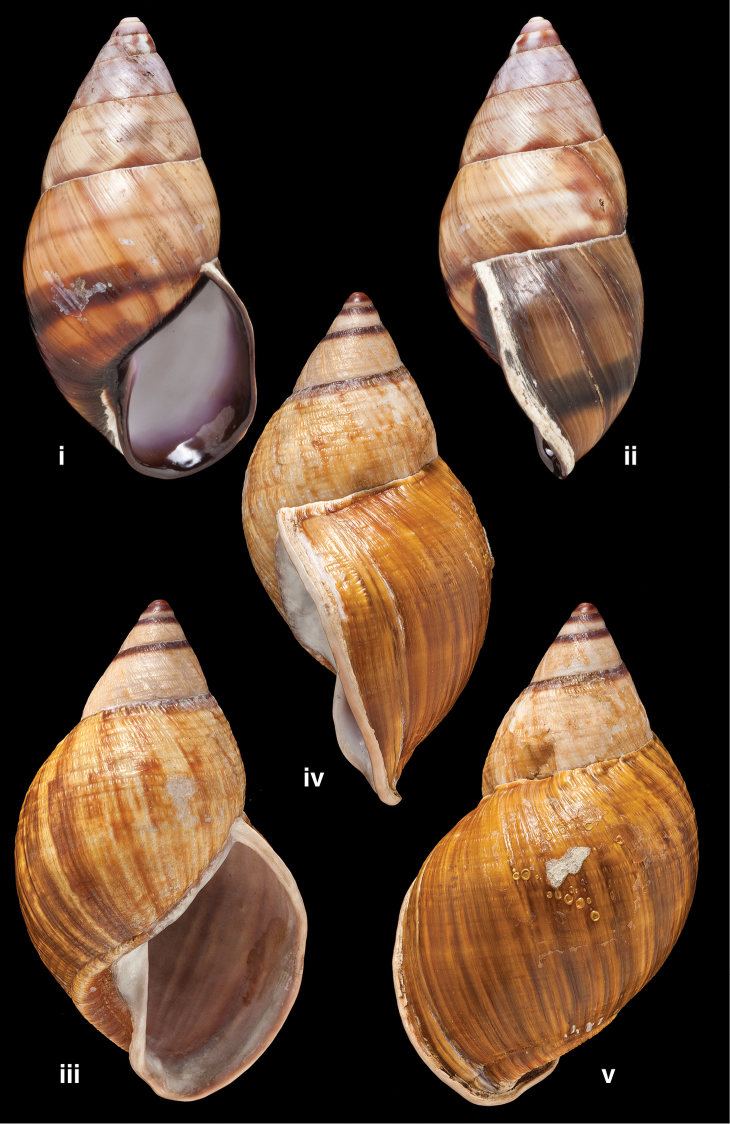
*Porphyrobaphe* species. **i–ii** Porphyrobaphe (Porphyrobaphe) approximata Fulton, 1896, syntype NHMUK 1895.12.19.44 (H = 65.7) **iii–v** Porphyrobaphe (Porphyrobaphe) iris (Pfeiffer, 1853), lectotype NHMUK 20100506 (H = 72.6).

**Figure 15.**
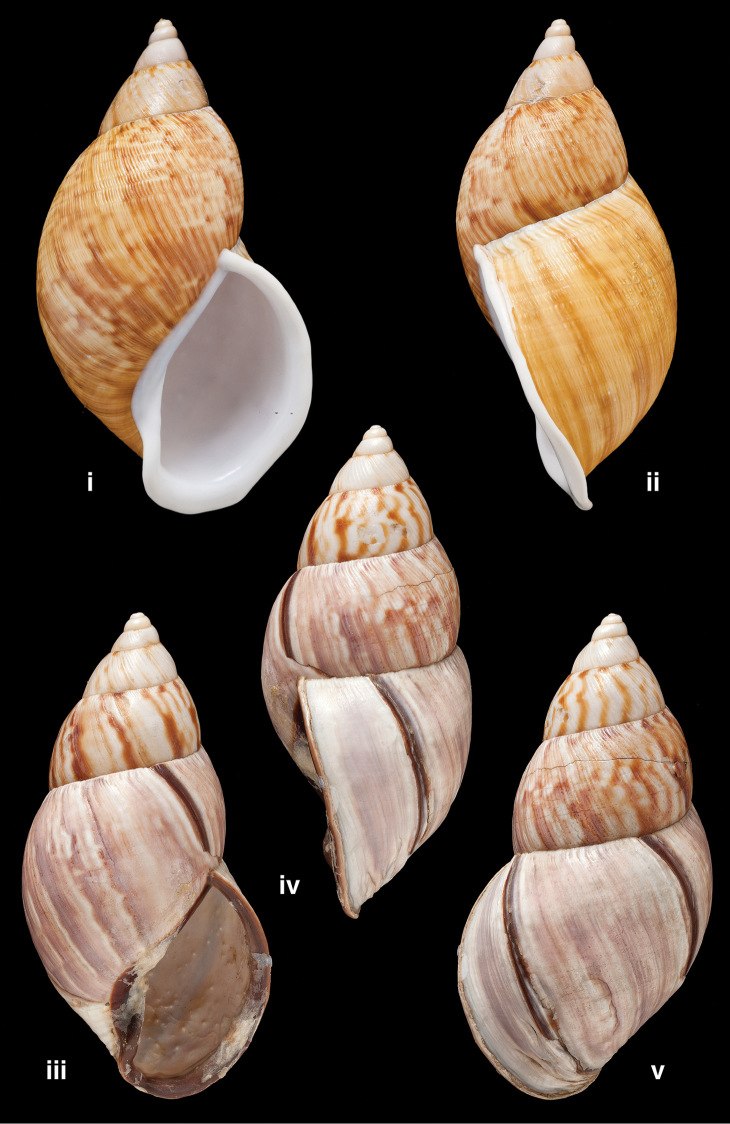
*Porphyrobaphe* species. **i–ii** Porphyrobaphe (Porphyrobaphe) irrorata (Reeve, 1849), syntype NHMUK 1975248 (H = 77.0) **iii–v** Porphyrobaphe (Porphyrobaphe) saturnus (Pfeiffer, 1860), syntype NHMUK 20140080 (H = 75.8).

**Figure 17.**
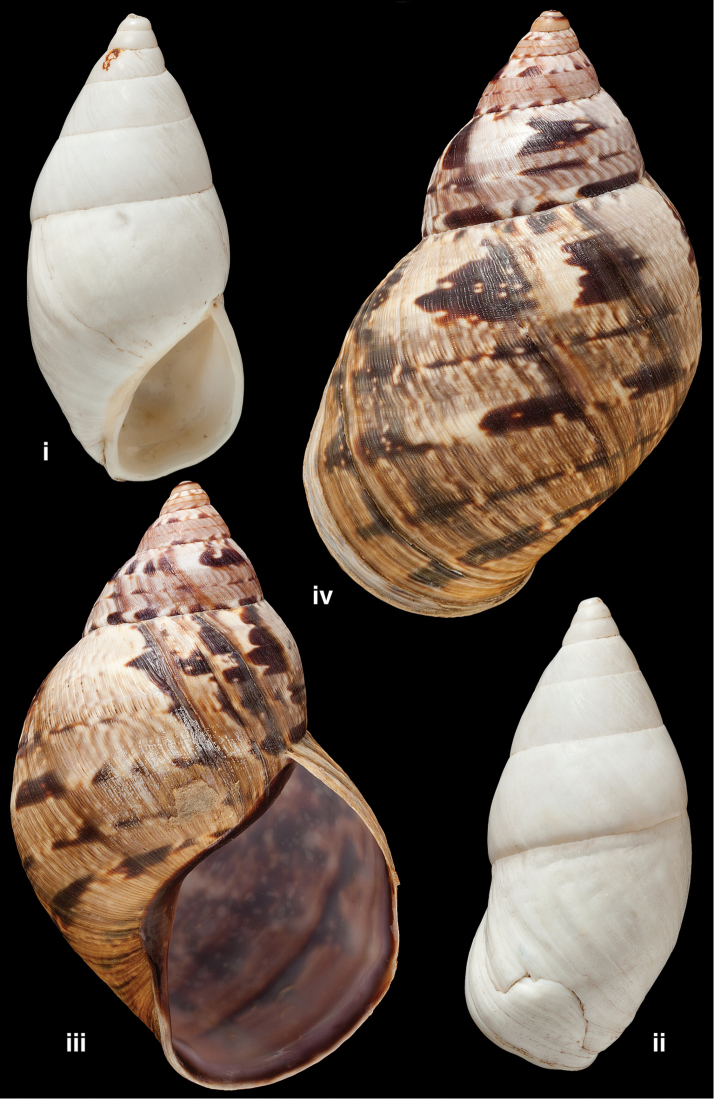
*Sultana* species. **i–ii** Sultana (Methorthalicus) powisiana (Petit de la Saussaye, 1843), holotype of Orthalicus powissianus var. niveus Preston, 1909 NHMUK 1909.8.18.85 (H = 65.2) **iii–iv** Sultana (Sultana) meobambensis (Pfeiffer, 1855), syntype NHMUK 20100505 (H = 84.9).

**Figure 18.**
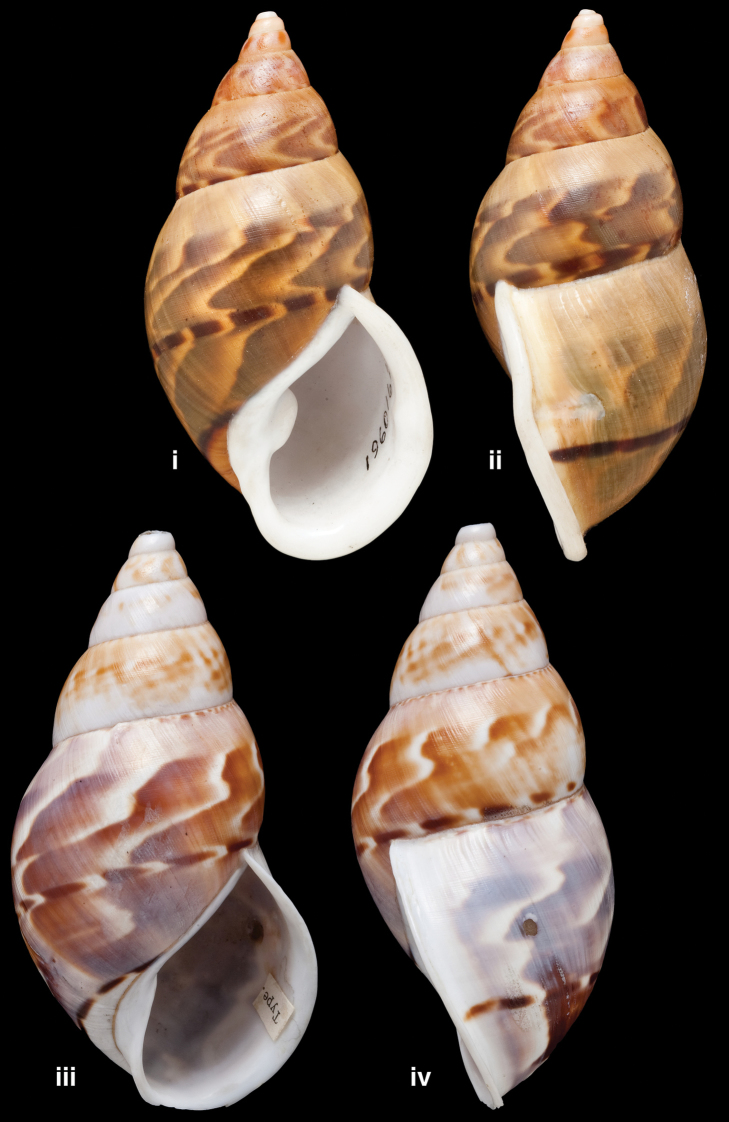
Sultana (Metorthalicus) deburghiae (Reeve, 1859). **i–ii** Lectotype NHMUK 19601622 (H = 64.7) **iii–iv** Lectotype of *Bulimus gloriosus* Pfeiffer, 1862 NHMUK 1975243 (H = 75.2).

**Figure 19.**
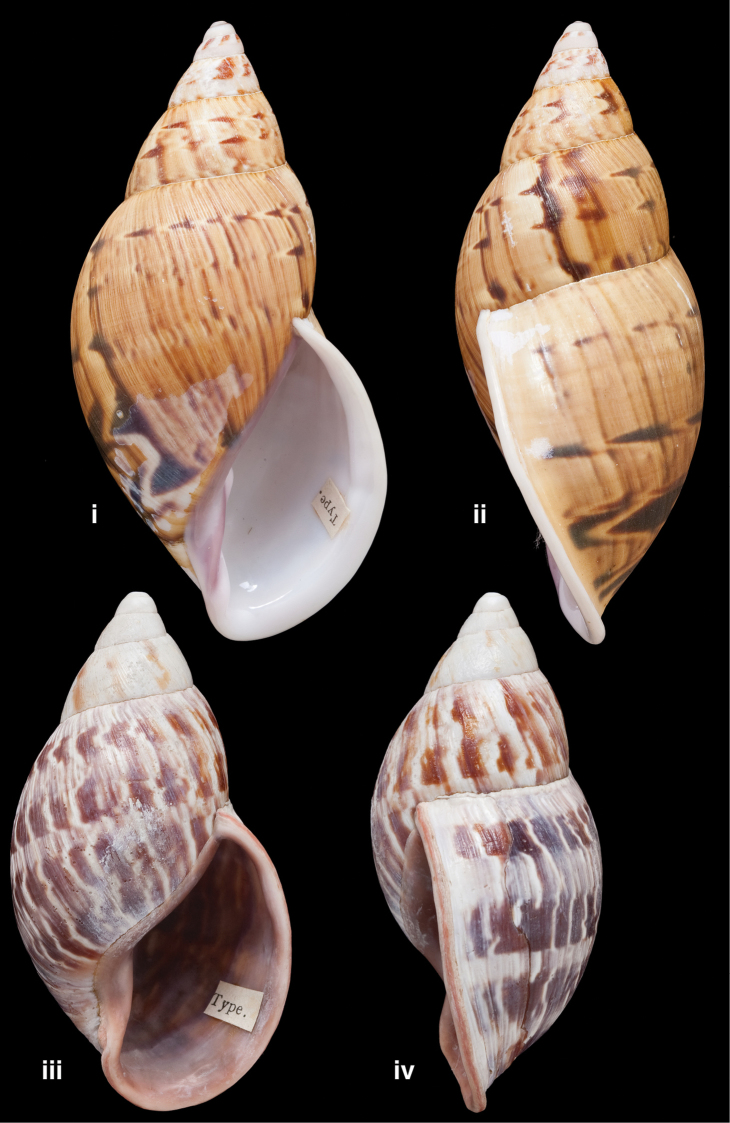
*Sultana* species. **i–ii** Sultana (Metorthalicus) fraseri (Pfeiffer, 1858), lectotype NHMUK 20140083 (H = 88.9) **iii–iv** Sultana (Metorthalicus) kellettii (Reeve, 1850), lectotype NHMUK 1975241 (H = 61.2).

**Figure 20.**
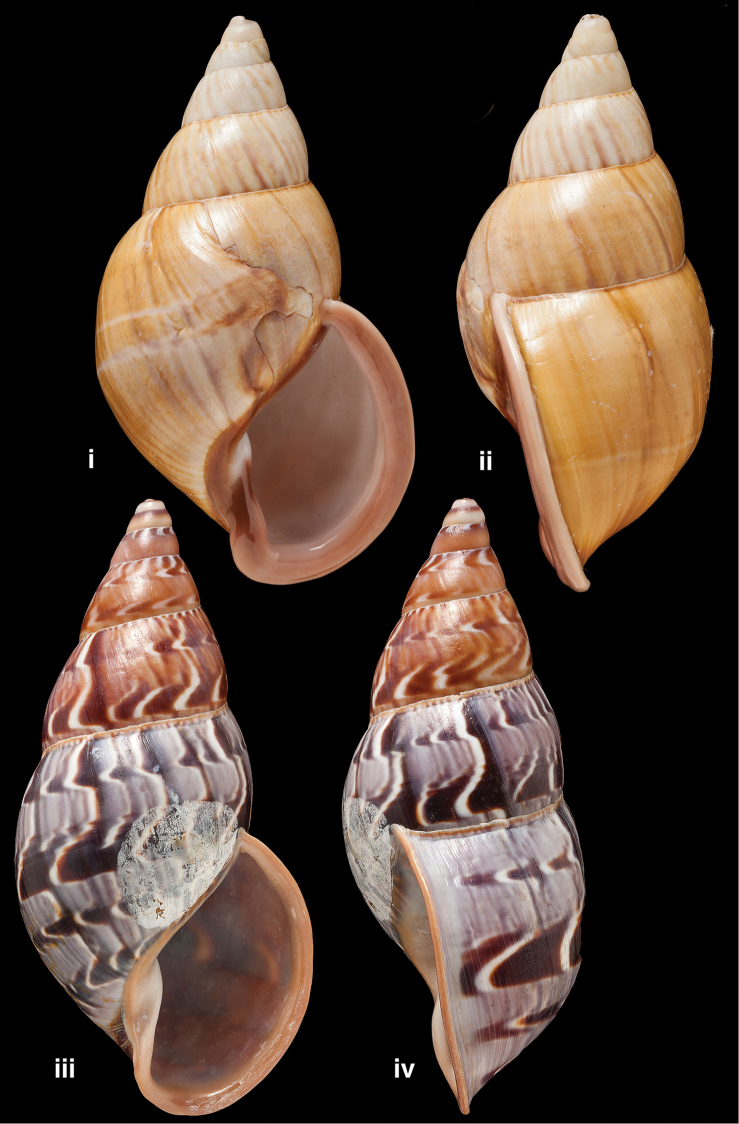
Sultana (Metorthalicus) yatesi (Pfeiffer, 1855) **i–ii** Holotype of *Porphyrobaphe vicaria* Fulton, 1896 NHMUK 20100507 (H = 82.2) **iii–iv** Lectotype NHMUK 1975239/1 (H 84.3).

**Figure 21.**
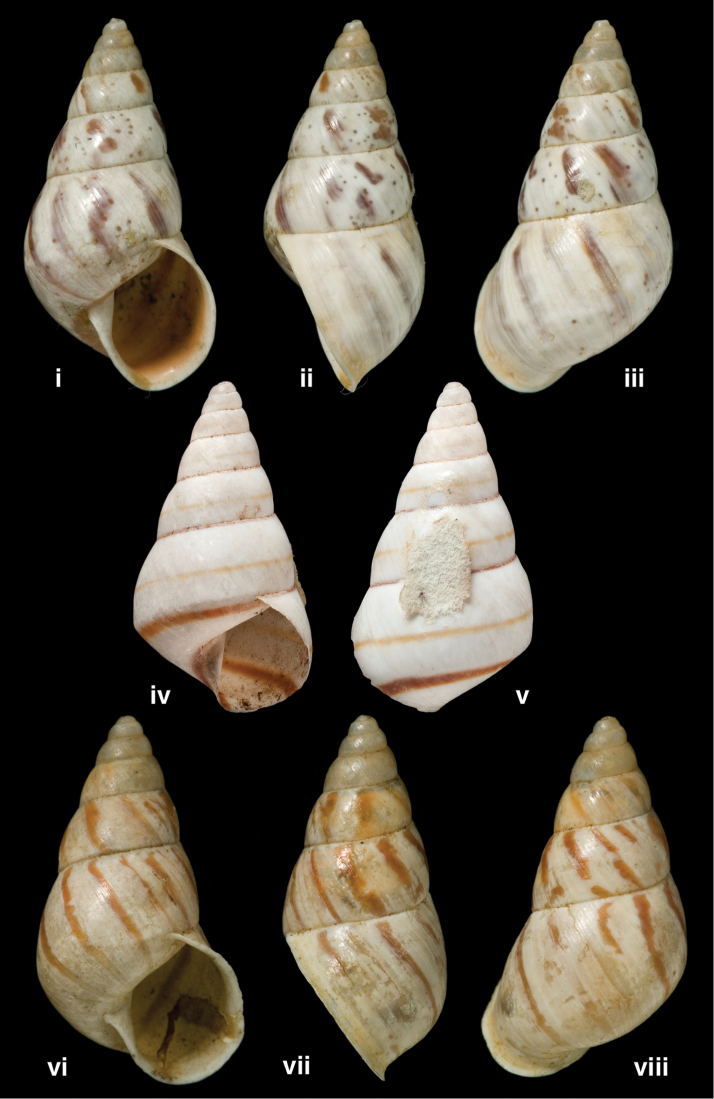
*Leiostracus* species. **i–iii** *Leiostracus clouei* (Pfeiffer, 1857), lectotype NHMUK 1975491 (H = 22.2) **iv–v** *Leiostracus obliquus* (Reeve, 1849), syntype of *Bulimus jeffreysi* Pfeiffer, 1852 NHMUK 20110083 (H = 20.4) **vi–viii** *Leiostracus demerarensis* (Pfeiffer, 1861), lectotype NHMUK 1975501 (H = 20.1).

*Bulinus cactivorus* Broderip in Broderip and Sowerby I 1832: 31—Five specimens were found in the General Collection, of which one may have been illustrated by Sowerby I 1833 [Sowerby I and II 1832–1841]: fig. 2. However, the label reads “Peru”, and thus does not correspond to the type locality given in the original publication (“Montechris in West Columbia”).

*Bulimus cantatus*Reeve 1848 [1848–1850]: pl. 56 fig. 375.—Described from “Mus. Denisson”.

*Bulimus cardinalis*Pfeiffer 1853b: 316.—Described from “Mus. Cuming”, but not found.

*Bulimus castelnaui*Pfeiffer 1857c: 332.— Described from “the collection of H. Cuming”, but not found.

*Bulimus castrensis*Pfeiffer 1847: 115.— Described from “the collection of H. Cuming”, but not found.

Bulimulus (Drymaeus) chacoensisPreston 1907: 491, fig. 5.

*Bulimus coerulescens*Pfeiffer 1858: 257.— Described from “the collection of H. Cuming”, but not found.

*Bulimus columellaris*Reeve 1849 [1848–1850]: pl. 73 fig. 528.—Described from “Mus. Cuming”, but not found.

*Bulimus confinus*Reeve 1850 [1848–1850]: pl. 86 fig. 643.—Described from “Mus. Cuming”, but not found.

*Bulimus coniformis*Pfeiffer 1847: 114.— Described from “the collection of H. Cuming”, with type locality [Venezuela] “Merida, Andes of Bolivia”; material found in the Cuming collection have lost their label with Pfeiffer’s handwriting and have “Venezuela” as locality, and is not considered type material.

*Bulimus constrictus*Reeve 1848 [1848–1850]: pl. 47 fig. 307.— Described from “Mus. Cuming”, but not found.

*Bulimus contortuplicatus*Reeve 1850 [1848–1850]: pl. 88 fig. 658.—Described from “Mus. Miers”.

*Bulinus coquimbensis* Broderip in Broderip and Sowerby I 1832: 30.— Described from “the collection of H. Cuming”, but not found.

*Bulinus corneus*Sowerby I 1833: 37.—Described from “shells collected by Mr. Cuming”, but not found.

*Bulinus corrugatus* King in King and Broderip 1831: 341.—“A specimen is deposited in the British Museum”, but has not been found.

*Bulimus curianianus*Reeve 1849 [1848–1850]: pl. 58 fig. 390.—Described from “Mus. Dyson”.

*Bulinus decoloratus*Sowerby I 1833: 73.—Described from “shells collected by Mr. Cuming”, but not found.

*Bulimus draparnaudi*Pfeiffer 1847: 113.— Described from “the collection of H. Cuming”, but not found.

*Bulimus droueti*Pfeiffer 1857b: 319, pl. 35 fig. 12.—The material found is from the Cuming collection, but lacks evidence that is was collected by Sallé.

*Bulimus eganus* Pfeiffer 1853 in Küster and Pfeiffer 1840–1865: 85, pl. 30 figs 11–12.—Described “Aus H. Cuming’s Sammlung”, but not found.

*Bulinus erythrostoma*Sowerby I 1833: 37.—Described from “shells collected by Mr. Cuming”, but not found.

*Partula flavescens* King in King and Broderip 1831: 342.—“*Mus. Brit., nost.* [King coll.], *Brod.*”; not found.

*Bulinus granulosus* Broderip in Broderip and Sowerby I 1832: 31.—Described from “the collection made by Mr. Cuming”, but not found.

*Bulinus gravesii* King in King and Broderip 1831: 340.—“*Mus. nost.*” [King coll.].

*Plekocheilus glaber grenadensis*Guppy 1868: 436.—See also Dance 1966: 288.

*Bulimus guentheri*Sowerby III 1892: 296, pl. 23 figs 7–8.—Sowerby wrote “[t]he only specimen I have seen belongs to the National Collection at South Kensington [NHMUK]”, but has not been encountered.

*Bulimus guttula*Pfeiffer 1854c: 154.—Description based on material “collected by M. Bourcier”, and presumed to be in NHMUK but not found.

*Bulimus hegewischi*Pfeiffer 1842: 46.—Described from “[Mexico] Tenango” and a colour variety from “Michoacan, Pazquaro. (Hegewisch in litt.)”; specimens found in the Cuming collection are labeled “Rio Frio”, and not considered type material.

*Bulimus hennahi*J.E. Gray 1828: 5, pl. 5 fig. 5.

*Bulimus hyematus*Reeve 1848 [1848–1850]: pl. 49 fig. 324.—Described from “Mus. Cuming”, but not found.

Otostomus (Drymaeus) lilacinus ictericusMartens 1893 [1890–1901]: 202.

*Bulimus inaequalis*Pfeiffer 1857c: 330.—Described from “the collection of H. Cuming”; the material found has no locality label.

*Bulinus inflatus* Broderip, 1836: 45.—Described from a shell “brought home by Mr. Cuming”, but not encountered in the collection.

*Bulimus iostoma*Sowerby I 1824: 58, pl. 5 fig. 1.

*Bulinus janeirensis* Sowerby I 1838 [Sowerby I and II 1832–1841]: 8, fig. 97.

*Bulimus jucundus*Pfeiffer 1855b: 290.—Described “from Mr. Cuming’s collection”, but not found.

*Bulinus labeo*Broderip 1828: 222, suppl. pl. 31.—See also Pain 1959.

*Bulinus laurentii*Sowerby I 1833: 37.—Described from “shells collected by Mr. Cuming”, but not found.

*Bulimus lindeni*Reeve 1848 [1848–1850]: pl. 31 fig. 189.—Described from “Mus. Cuming”, but not found.

*Helix listeri*Wood 1828: 22, pl. 7 fig. 23.—“Br.M.” [NHMUK], not found.

*Orthalicus macandrewi*Sowerby III 1889: 398, pl. 25 fig. 18.—Based on a “single specimen”, which is, however, not present in the NHMUK collection.

*Bulinus mutabilis* Broderip in Broderip and Sowerby I 1832: 108—Several lots are present in the General Collection, however, none matching the original data.

*Bulimus navarrensis*Angas 1878: 73, pl. 5 figs 15–16.—“(Mus. Boucard)”.

*Otostomus chiapensis nebulosus*Martens 1893 [1890–1901]: 205, pl. 12 fig. 15.—Based on Strebel and Pfeffer (1882).

Bulimulus (Drymaeus) nigroumbilicatusPreston 1907: 491, fig. 6.

*Helix orobaena*d’Orbigny 1835: 17.— This taxon is marked by Gray (1854: 18) as being absent, thus the material in MNHN is the sole extant.

*Bulinus pallidior*Sowerby I 1833: 72.—Sowerby wrote “Mr. Cuming obtained two specimens of this species in South America, but without being able to ascertain its locality”. The material found is from the Cuming collection, and has a label “Central America”; since it comprises four specimens it is not considered as type material.

*Bulimus pardalis*Reeve 1848 [1848–1850]: pl. 24 fig. 157.—“Mus. Dennison”.

*Bulimus peelii*Reeve 1859: 123.—This species has been mentioned by Richardson both as *Porphyrobaphe* (Richardson 1993: 120) and *Drymaeus* (Richardson 1995: 161); we consider only the latter classification to be correct.

*Bulimus pentlandi*Reeve 1849 [1848–1850]: pl. 83 fig. 614.—“Mus. Hamilton”.

*Otostomus attenuatus pittieri*Martens 1893 [1890–1901]: 216, pl. 16 fig. 1.—Based on material collected by Pittier (see Angas 1879: 478).

*Bulimus primularis*Reeve 1849 [1848–1850]: pl. 73 fig. 527.—Based on material from “Mus. Cuming”, but not located.

*Bulinus princeps* Broderip in Sowerby I 1833 [Sowerby I and II 1832–1841]: 6, fig. 18.

*Tomigerus principalis*Sowerby II 1849: 14, pl. 2 figs 6–7.—“In Mr. Cuming’s collection”, but not located.

*Bulinus pulchellus* Broderip in Broderip and Sowerby I 1832: 106.—Described from “shells collected by Mr. Cuming”, but not found.

*Bulinus pulchellus* Sowerby I 1838 [Sowerby I and II 1832–1841]: 8, figs 91–92 (not Broderip 1832).

*Bulimus rhodacme*Pfeiffer 1842: 50.—“(Bridges, Cuming)”, but material not located.

*Bulinus rubellus*Broderip 1832: 124.— Described from “shells collected by Mr. Cuming”, but not found.

*Bulimus rubescens*Reeve 1848 [1848–1850]: pl. 23 fig. 148.—“(Mus. Cuming)”, not found.

*Bulimus rupicolus*Reeve 1848 [1848–1850]: pl. 16 fig. 93.—“(Mus. Cuming)”, not found.

*Bulimus sarcodes*Pfeiffer 1846: 30.—Described from “the collection of H. Cuming”; the material was not found.

*Bulimus sayi*Pfeiffer 1847: 114.—Based on material “in the collection of Hugh Cuming”, but not located.

*Bulimus scytodes*Pfeiffer 1853b: 256.—Described from “the collection of Hugh Cuming”; the material was not found.

*Bulimus sporadicus*Reeve 1848 [1848–1850]: pl. 49 fig. 325.—“(Mus. Cuming)”, not found.

*Bulinus striatulus*Sowerby I 1833: 73.—Described from “shells collected by Mr. Cuming”, but not found.

*Bulinus striatus* ‘King’ Sowerby I 1833 [Sowerby I and II 1832–1841]: 7, fig. 56.

Clausilia? (Balea?) tayloriPfeiffer 1861b: 27, pl. 2 fig. 7.—Described from “the collection of H. Cuming”, but the material has not been found.

*Bulinus tigris* Broderip in Broderip and Sowerby I 1832: 107.—Described from “shells collected by Mr. Cuming”, but not found.

Otostomus (Drymaeus) lilacinus undulosusMartens 1893 [1890–1901]: 201.—Based on material collected by Champion, but not found.

*Bulinus varians* Broderip in Broderip and Sowerby I 1832: 107.—Described from “shells collected by Mr. Cuming”, but not found.

*Tomigerus venezuelensis*Pfeiffer 1856: 36.—Described from “the collection of H. Cuming”, but the material has not been found.

*Bulimus venosus*Reeve 1848 [1848–1850]: pl. 45 fig. 285.—“(Mus. Cuming)”, not found.

Bulimulus (Drymaeus) ventricosusPreston 1907: 495, fig. 10.

*Bulinus vexillum* Broderip in Broderip and Sowerby I 1832: 105.—Described from “shells collected by Mr. Cuming”, but not found.

*Helix vexillum*Wood 1828: 24, pl. 8 fig. 78a.—“M.Cab.” [Mrs. Mawe’s coll.].

Bulimulus (Drymaeus) vicinusPreston 1907: 495, fig. 11.

*Bilinus vittatus* Broderip in Broderip and Sowerby I 1832: 31.—Described from “shells collected by Mr. Cuming”, but not found.

*Bulimus ziegleri*Reeve 1849 [1848–1850]: pl. 58 fig. 389.—“(Mus. Cuming)”.

#### IV. Addendum to part 2, Bothriembryontidae and Odontostomidae (Breure and Ablett 2012)

##### Bulimus senilis

Taxon classificationAnimaliaStylommatophoraBulimulidae

Gassies, 1869

Bulimus senilis Gassies 1869: 71.
Placostylus senilis ; Neubeurt et al. 2009: 110, fig. 18 (lectotype designation).

###### Type locality.

[New Caledonia] “Baie du Sud (Nov. Cal.)” (see remarks).

###### Label.

“New Caledonia”.

###### Dimensions.

“Long. 129 mill., diam. maj. 65”. Figured specimen H 126.1, D 56.3, W 7.0.

###### Type material.

NHMUK 1883.11.10.1179, one paralectotype ex Sowerby ex Gassies.

###### Remarks.

Neubert et al. (2009) suggested that the type locality may be erroneous as this taxon is only known from Ile des Pins and Koutoumo. See also their discussion on the variation within this species and consider their general remark that this species is “either recently extinct and/or represent morphological variations of extant taxa”. The lectotype selected by Neubert et al. (2009) is MNHN 21367.

###### Current systematic position.

Bothriembryontidae, *Placostylus senilis* (Gassies, 1869).

#### V. Corrigenda to part 2, Bothriembryontidae and Odontostomidae (Breure and Ablett 2012)

**Figure 22.**
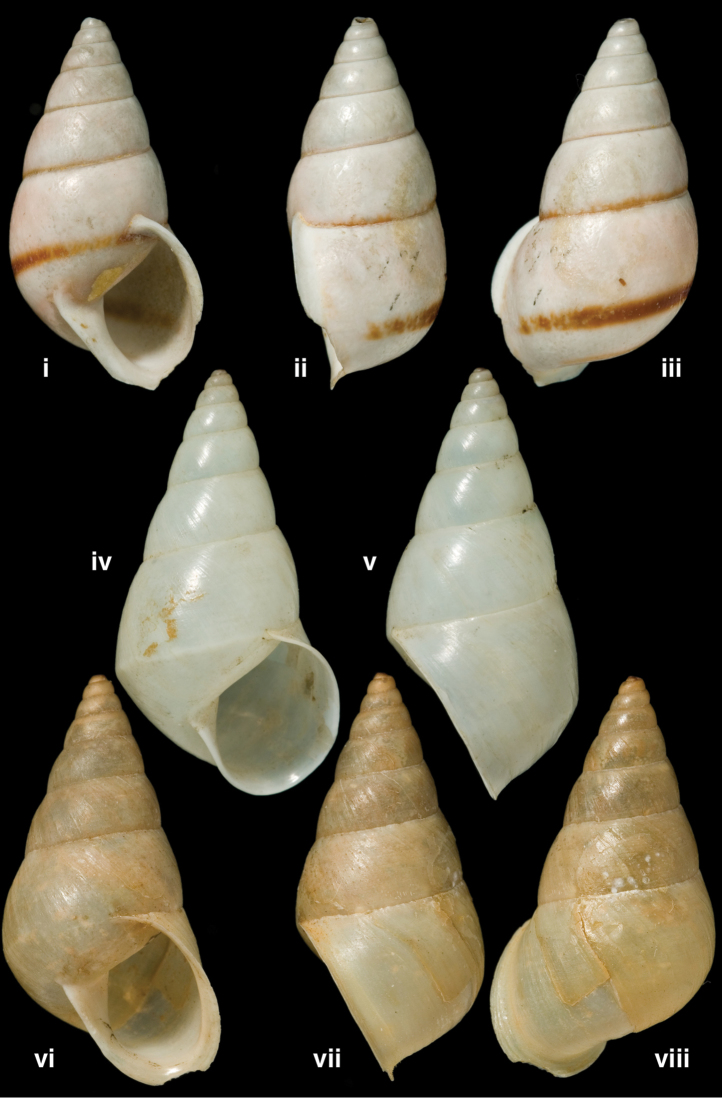
*Leiostracus* species. **i–iii** *Leiostracus obliquus* (Reeve, 1849), lectotype NHMUK 1975493 (H = 22.7) **iv–v** *Leiostracus perlucidus* (Spix, 1827), probable syntype of *Bulinus opalinus* Sowerby I, 1833 NHMUK 1975442 (H 27.8) **vi–viii** *Leiostracus sarcochilus* (Pfeiffer, 1857), lectotype NHMUK 1975398 (H = 24.7).

**Figure 23.**
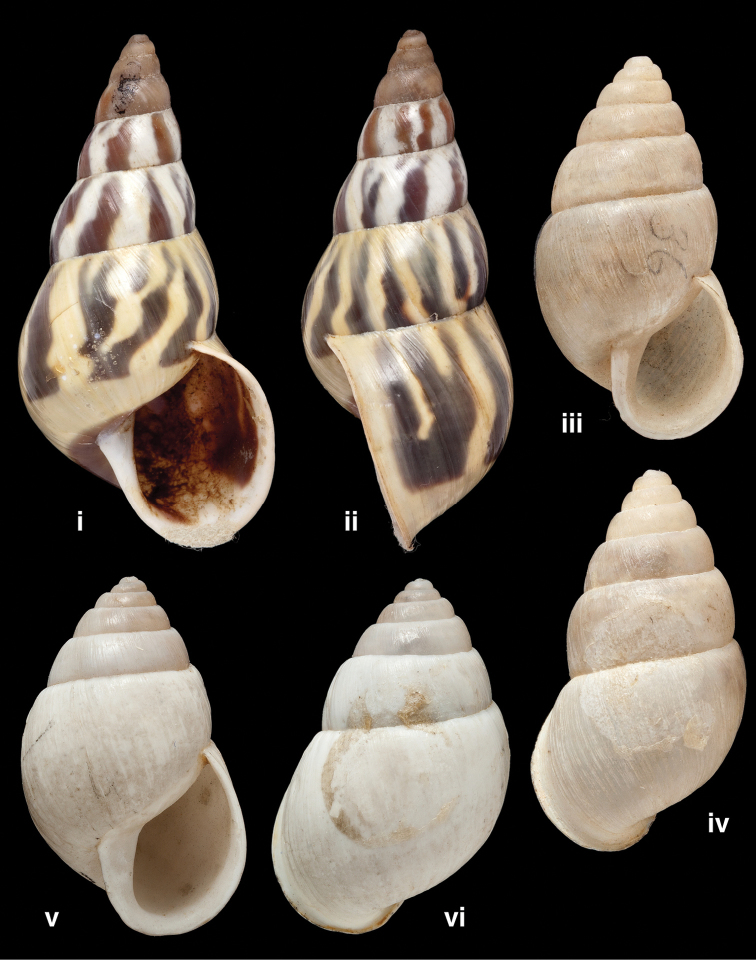
**i–ii** *Leiostracus subtuszonatus* (Pilsbry, 1899), probable paralectotype of *Bulimus onager* Reeve, 1848 NHMUK 20130094 (H = 29.0) **iii–vi** *Rhinus* species. **iii–iv** *Rhinus constrictus* (Pfeiffer, 1841), lectotype of *Bulimus hyaloideus* Pfeiffer, 1855 NHMUK 1975412 (H = 20.6) **v–vi** *Rhinus ovulum* (Reeve, 1849), lectotype NHMUK 1975416 (H = 20.1).

**Figure 24.**
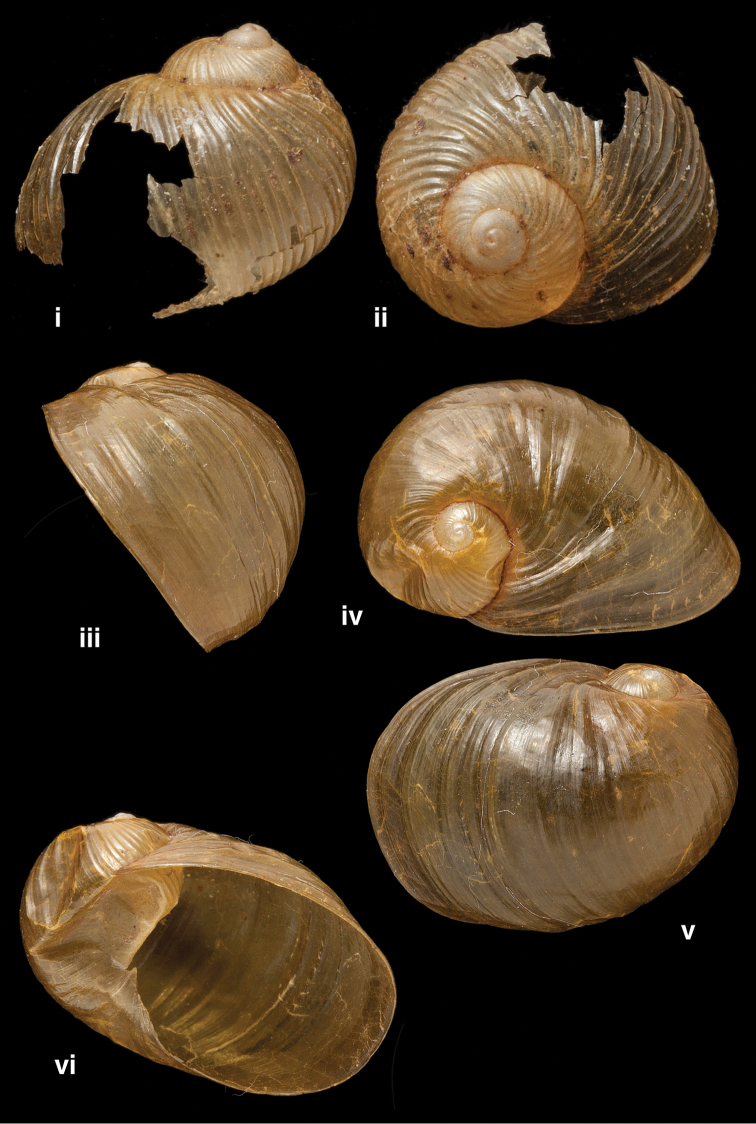
*Simpulopsis* species. **i–ii** Simpulopsis (Simpulopsis) corrugata Guppy, 1866, syntype NHMUK 1866.1.3.7 (H = 9.27) **iii–vi** Simpulopsis (Simpulopsis) cumingi Pfeiffer, 1861, lectotype NHMUK 1975486 (H = 14.1).

**Figure 25.**
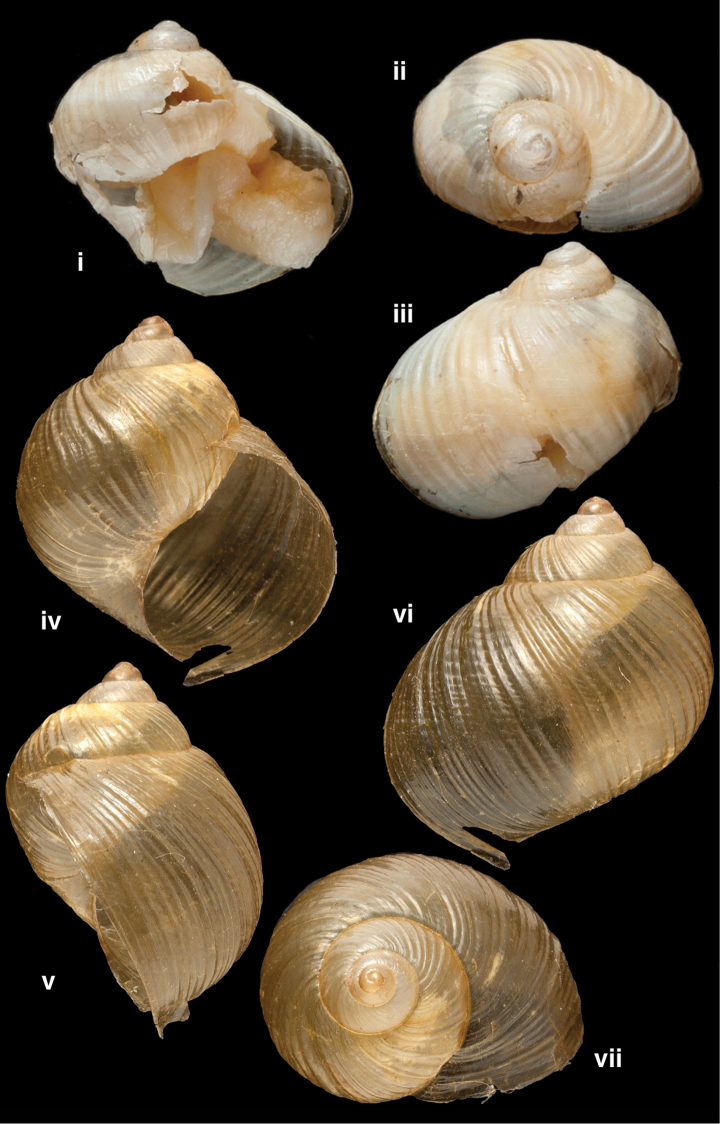
*Simpulopsis* species. **i–iii** Simpulopsis (Simpulopsis) gomesae da Silva & Thomé, 2006, paratype (H = 6.46) **iv–vii** Simpulopsis (Simpulopsis) decussata Pfeiffer, 1857, lectotype NHMUK 1975488 (H = 14.3).

**Figure 26.**
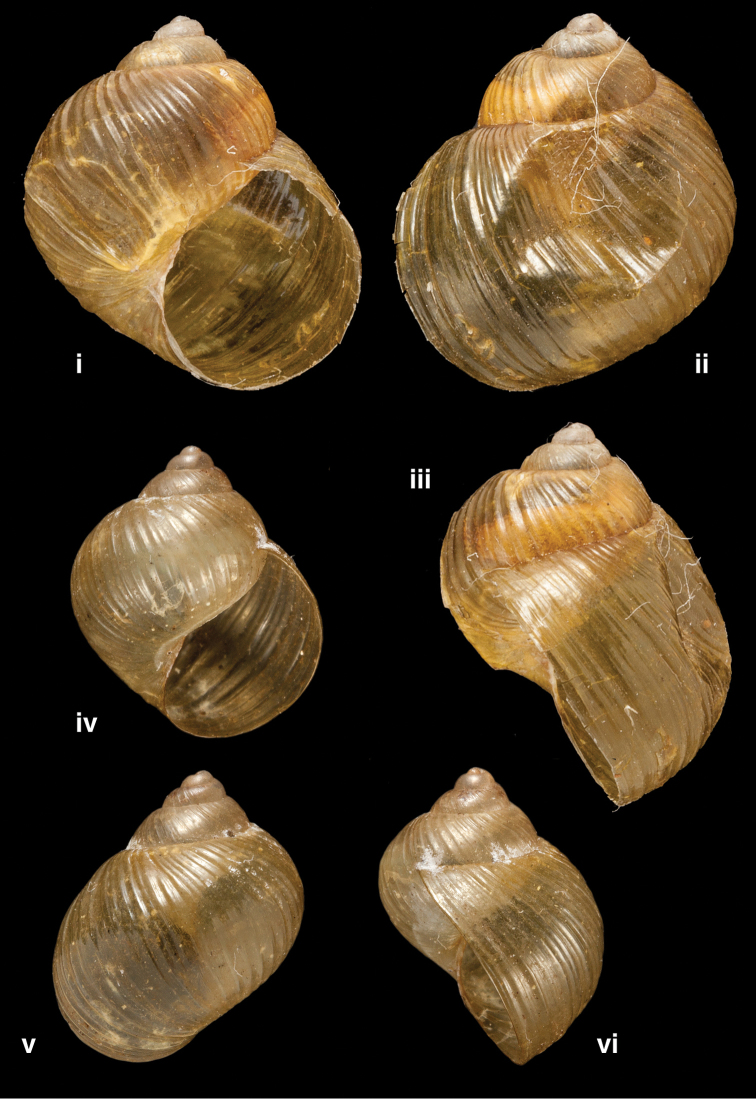
*Simpulopsis* species. **i–iii** Simpulopsis (Simpulopsis) rufovirens (Moricand, 1846), lectotype of *Vitrina salomonia* Pfeiffer, 1853 NHMUK 1975485 (H = 11.1) **iv–vi** Simpulopsis (Simpulopsis) simula (Morelet, 1851), syntype NHMUK 1893.2.4.1128 (H = 8.26).

**Figure 27.**
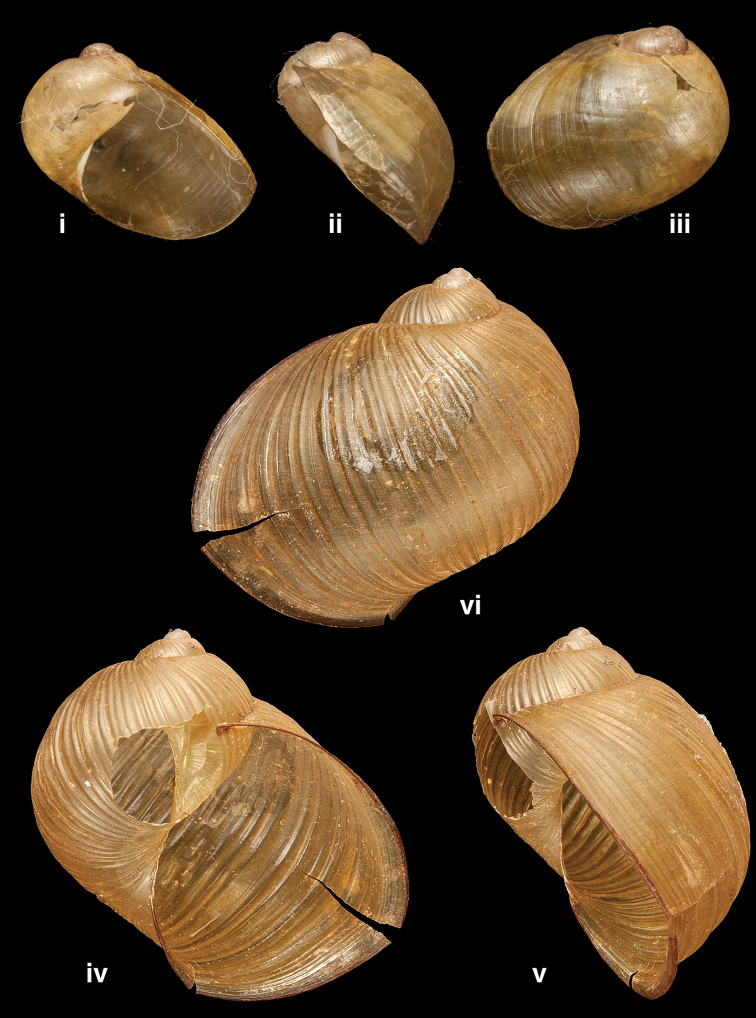
*Simpulopsis* species. **i–iii** Simpulopsis (Simpulopsis) aenea Pfeiffer, 1861, syntype NHMUK 1975229 (H = 8.98) **iv–vi** Simpulopsis (Simpulopsis) miersi Pfeiffer, 1857, lectotype NHMUK 1975489 (H = 20.6).

**Figure 28.**
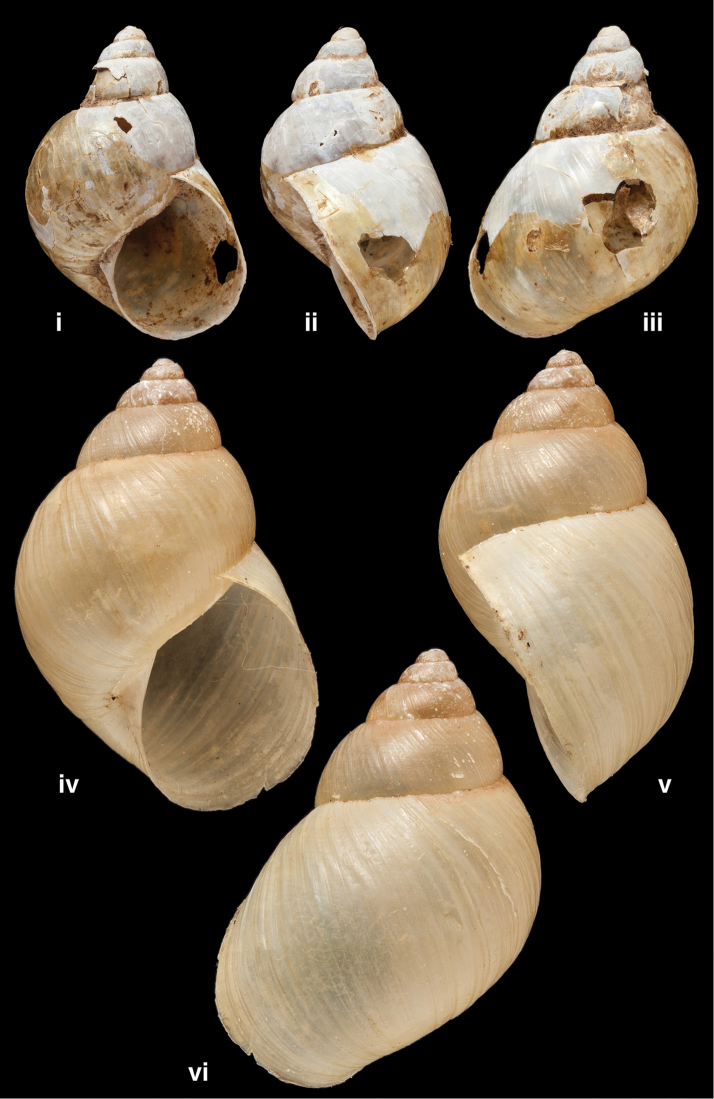
*Simpulopsis* species. **i–iii** Simpulopsis (Eudioptus) citrinovitrea (Moricand, 1836), syntype of *Bulimus marmatensis* Pfeiffer, 1855 NHMUK 1975330 (H = 15.0) **iv–vi** Simpulopsis (Eudioptus) ephippium Ancey (1904), paralectotype NHMUK 1905.12.30.12 (H = 20.5).

**Figure 29.**
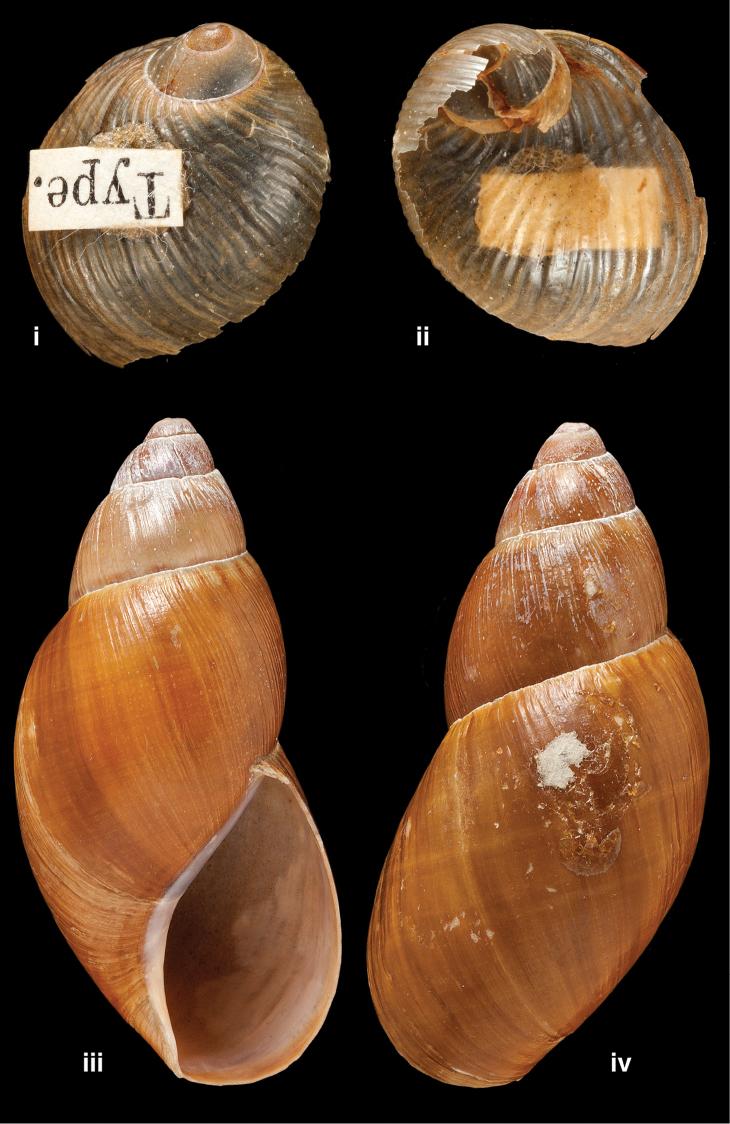
**i–ii** Simpulopsis (Simpulopsis) vincentina E.A. Smith, 1895, holotype NHMUK 1895.6.17.458 (H = 11.4) **iii–iv** *Thaumastus requieni* (Pfeiffer, 1853), lectotype NHMUK 1975301 (H = 62.0).

**Figure 30.**
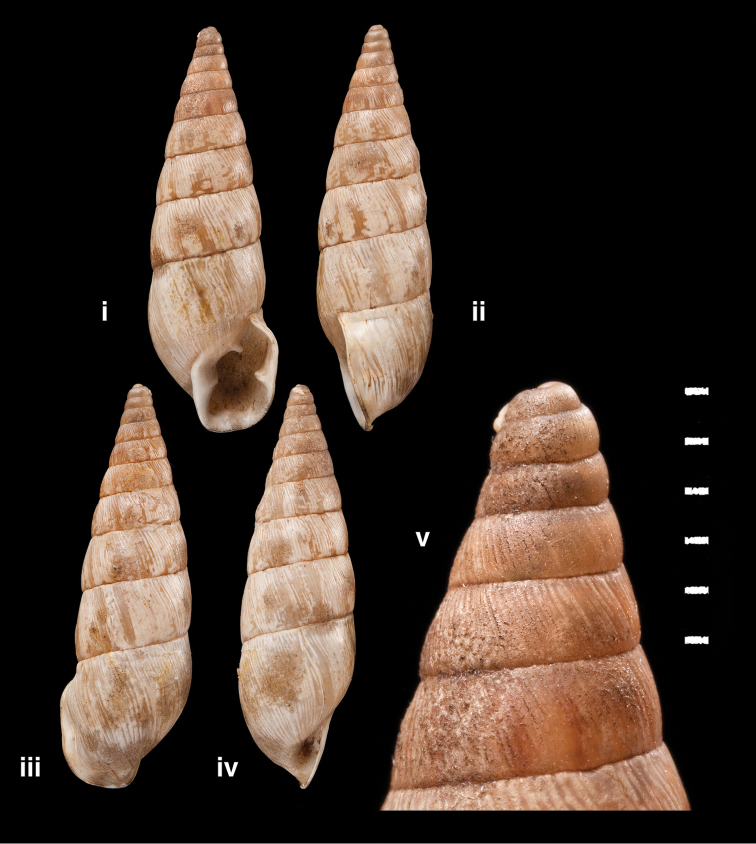
**i–v** *Spixia striata* (Spix, 1827), lectotype of *Pupa spixii major* d’Orbigny, 1837 NHMUK 1854.12.4.230 (H = 34.8) **v** apical whorls (scale line = 5 mm).

p. 3: ***salomonis*** (Pfeiffer, 1853) shoud be removed under *Placostylus* Beck, 1837, and inserted under *Santacharis* Iredale, 1927.

p. 25: ***Pupa spixii major*** d’Orbigny, 1837 under Type material: the lectotype has registration number 1854.12.4.230 instead of 1885.12.4.232. The latter lot is from “Corrientes, Argentina”, while lot 1854.12.4.230 is from “Guarayos, Bolivia”. The specimen figured in Figs 22A–E is actually a paralectotype from this locality; for the lectotype see Figure 30 in this paper.

p. 30: ***Bulimus ouensis*** Gassies, 1870 under Type material: the holotype has registration number 1883.11.10.1176 where it should read 1883.11.10.1167.

#### VI. Addendum to part 3, Bulimulidae (Breure and Ablett 2014)

##### Bulimus diaphanus

Taxon classificationAnimaliaStylommatophoraBulimulidae

Pfeiffer, 1855

Bulimus diaphanus Pfeiffer 1855a: 125; Pfeiffer 1859: 505; Breure 1979: 62.
Bulimulus diaphanus ; Pilsbry 1897 [1897–1898]: 47.
Bulimulus (Bulimulus) diaphanus diaphanus ; Breure 1974: 30, pl. III fis 11–12.

###### Type locality.

“S. Thomas, West Indies (*Bland*)”.

###### Dimensions.

“Long. 15, diam. 7 mill.”.

###### Remarks.

The two specimens mentioned and figured by Breure (1974) have to be considered as lost, as—despite repetitive searches—they could not be re-found during our research.

##### Bulimulus (Drymaeus) interruptus pallidus

Taxon classificationAnimaliaStylommatophoraOrthalicidae

Preston, 1909

Bulimulus (Drymaeus) interruptus var. pallidus Preston 1909: 511, fig. 2.

###### Type locality.

“Merida, Venezuela”.

###### Label.

“Merida, Venezuela”.

###### Dimensions.

Not given. Figured specimen H 23.5, D 11.9, W 4.3+.

###### Type material.

NHMUK 1914.4.3.41, lectotype (ex Preston).

###### Remarks.

This varietal name has been treated as unavailable under Art. 45.6 ICZN by Breure and Ablett (2014: 96). Paul Callomon (pers. commun.) has suggested that this should be reconsidered and doubted if a lectotype of the nominal name already existed.

**Our opinion is as follows:**

- Preston undoubtedly had a series of specimens at hand when describing Bulimulus (Drymaeus) interruptus; both the wording “to be greatly variable” and “its principle forms” are indicative of this.
- Breure (1979: 120) mentioned this taxon in his listing under Drymaeus (Mesembrinus) Albers, 1850, and stated “HT BMNH 1914.4.3.38” [referring to a single specimen, thus qualifying Art. 74.6.1.2 jo. 74.3]; Art. 74.6 ICZN rules this statement as a lectotype designation. It should be noted, however, that Breure did not list the “var. *pallidus*” of Preston in his paper (cf. point d below).
- Köhler (1997) also concluded that this taxon was described from several specimens but said “A holotype has not been designated. Therefore, the present specimen [ZMB 59597] is a syntype. Consequently, the specimen in the BMNH referred to as holotype by Breure (1979) is a syntype”. Overlooking, as explained in the previous item, the lectotype designation under Art. 74.6 (see previous point).
- Reconsidering the (un)availability under Art. 45.6, it is important to note that var. *pallidus* was proposed before 1961 and has to be treated as subspecific (see the contributions of Steve Lingafelter and Doug Yanega on the Taxacom listing, http://to.ly/zFZO). It may be noted that the only reference to this taxon after Preston’s publication is in Baker (1926: 44), who regarded it as a synonym of *Bulimus granadensis* Pfeiffer, 1848 (see also Richardson 1995: 133).
- While Bulimulus (Drymaeus) interruptus var. pallidus is an available name, we concur with Baker (1926) and Richardson (1995) to consider this taxon as a synonym of the nominal form.

The specimen of var. *pallidus* in the NHMUK is now designated lectotype (**design. n.**) to fixate this synonymisation. The text in Breure and Ablett (2014: 96), under ’Type material’, should be corrected as follows: “NHMUK 1914.3.38, lectotype; 1914.4.3.39–40, 42–43, four paralectotypes”.

###### Current systematic position.

Bulimulidae, Drymaeus (Mesembrinus) interruptus (Preston, 1909).

#### VII. Corrigenda to part 3, Bulimulidae (Breure and Ablett 2014)

The ’Systematic list of taxa arranged in generic order’ on page 3–7 should be replaced by the following text:

##### Family Bulimulidae Tryon, 1867

###### Auris

Taxon classificationAnimaliaStylommatophoraBulimulidae

Spix, 1827

swainsoni Pfeiffer, 1845.

###### Bostryx

Taxon classificationAnimaliaStylommatophoraBulimulidae

Troschel, 1847 sensu Breure 1979 (see also Breure 2012b)

acalles Pfeiffer, 1853; *affinis* Broderip in Broderip and Sowerby I 1832; *agueroi* Weyrauch, 1960; *aileenae* Breure, 1978; *albicans* Broderip in Broderip and Sowerby I 1832; *albicolor* Morelet, 1863; *albus* Sowerby I, 1833; *andoicus* Morelet, 1863; *apodemeta* d’Orbigny, 1835; *atacamensis* Pfeiffer, 1856; *balsanus* Morelet, 1863; *cactorum* d’Orbigny, 1835; *ceratacme* Pfeiffer, 1855; *cercicola*Morelet 1863; *compactus* Fulton, 1902; *conspersus* Sowerby I, 1833; *coriaceus* Pfeiffer, 1857; *costatus* Weyrauch, 1960; *costifer* Weyrauch, 1960; *delumbis* Reeve, 1849; *denickei* J.E. Gray, 1852; *depstus* Reeve, 1849; *derelictus* Broderip in Broderip and Sowerby I 1832; *devians* Dohrn, 1863; *emaciatus* Morelet, 1863; *erosus* Broderip in Broderip and Sowerby I 1832; *ferrugineus* Reeve, 1849; *glomeratus* Weyrauch, 1960; *guttatus* Broderip in Broderip and Sowerby I 1832; *hamiltoni* Reeve, 1849; *holostoma* Pfeiffer, 1846; *huascensis* Reeve, 1848; *infundibulum* Pfeiffer, 1853; *kathiae* Breure, 1978; *lactifluus* Pfeiffer, 1857; *lesueureanus* Morelet, 1860; *lichnorum* d’Orbigny, 1835; *limensis* Reeve, 1849; *limonoica* d’Orbigny, 1835; *longinquus* Morelet, 1863; *luridus* Pfeiffer, 1863; *mejillonensis* Pfeiffer, 1857; *metagyra* Pilsbry & Olsson, 1949; *minor* Weyrauch, 1960; *modestus* Broderip in Broderip and Sowerby I 1832; *moniezi* Dautzenberg, 1896; *montagnei* d’Orbigny, 1837; *mordani* Breure, 1978; *multispira* da Costa, 1904; *nanus* Reeve, 1849; *nigropileatus* Reeve, 1849; *obliquistriatus* da Costa, 1901; *orophilus* Morelet, 1860; *papillatus* Morelet, 1860; *paposensis* Pfeiffer, 1856; *paucicostatus* Breure, 1978; *philippii* Pfeiffer, 1842; *pictus* Pfeiffer, 1855; *pruinosus* Sowerby I, 1833; *pupiformis* Broderip in Broderip and Sowerby I 1832; *pustulosus* Broderip in Broderip and Sowerby I 1832; *radiatus* Morelet, 1863; *reconditus* Reeve, 1849; *rehderi* Weyrauch, 1960; *rhodolarynx* Reeve, 1849; *rodriguezae* Weyrauch, 1967; *rusticellus* Morelet, 1860; *scabiosus* Sowerby I, 1833; *scalaricosta* Morelet, 1860; *scalariformis* Broderip in Broderip and Sowerby I 1832; *serotinus* Morelet, 1860; *simpliculus* Pfeiffer, 1855; *spiculatus* Morelet, 1860; *stenacme* Pfeiffer, 1857; *terebralis* Pfeiffer, 1842; *torallyi* d’Orbigny, 1835; *tricinctus* Reeve, 1848; *tumidulus* Pfeiffer, 1842; *turritus* Broderip in Broderip and Sowerby I 1832; *umbilicaris* Souleyet, 1842; *veruculum* Morelet, 1860; *vilchezi* Weyrauch, 1960; *virgultorum* Morelet, 1863; *voithianus* Pfeiffer, 1847; *woodwardi* Pfeiffer, 1857.

###### Bulimulus

Taxon classificationAnimaliaStylommatophoraBulimulidae

Leach, 1814

barbadensis Pfeiffer, 1853; *cacticolus* Reeve, 1849; *dysoni* Pfeiffer, 1846; *effeminatus* Reeve, 1848; *erectus* Reeve 1849; *haplochrous* Pfeiffer, 1855; *heloica* d’Orbigny, 1835; *ignavus* Reeve, 1849; *inutilis* Reeve, 1850; *istapensis* Crosse and Fischer, 1873; *juvenilis* Pfeiffer, 1855; *marcidus* Pfeiffer, 1853; *mollicellus* Reeve, 1849; *monachus* Pfeiffer, 1857; *montevidensis* Pfeiffer, 1846; *nubeculatus* Pfeiffer, 1853; *pervius* Pfeiffer, 1853; *pessulatus* Reeve, 1848; *petenensis* Morelet, 1851; *pliculatus* Pfeiffer, 1857; *rubrifasciatus* Reeve, 1848; *sporadica* d’Orbigny, 1835; *transparens* Reeve, 1849; *turritella* d’Orbigny, 1835; *vesicalis* Pfeiffer, 1853.

###### Drymaeus (Drymaeus)

Taxon classificationAnimaliaStylommatophoraBulimulidae

Albers, 1850

abruptus Rolle, 1904; *abscissus* Pfeiffer, 1855; *abyssorum* d’Orbigny, 1835; *aequatorianus* E.A. Smith, 1877; *acervatus* Pfeiffer, 1857; *acuminatus* da Costa, 1906; *alabastrinus* da Costa, 1906; *albolabiatus* E.A. Smith, 1877; *ambustus* Reeve, 1849; *angustus* da Costa, 1906; *antioquiensis* Pfeiffer, 1855; *arcuatostriatus* Pfeiffer, 1855; *auris* Pfeiffer, 1866; *baranguillanus* Pfeiffer, 1853; *bartletti* H. Adams, 1867; *bellus* da Costa, 1906; *bogotensis* Pfeiffer, 1855; *bolivarii* d’Orbigny, 1835; *bolivianus* Pfeiffer, 1846; *boucardi* da Costa, 1907; *bourcieri* Pfeiffer, 1853; *brachysoma* d’Orbigny, 1835; *buckleyi* Sowerby III, 1895; *canaliculatus* Pfeiffer, 1845; *castaneostrigatus* da Costa, 1906; *caucaensis* da Costa, 1898; *chamaeleon* Pfeiffer, 1855; *chimborasensis* Reeve, 1848; *chiriquensis* da Costa, 1901; *clathratus* Pfeiffer, 1858; *coarctatus* Pfeiffer, 1845; *confluens* Pfeiffer, 1855; *convexus* Pfeiffer, 1855; *cuticula* Pfeiffer, 1855; *cuzcoensis* Reeve, 1849; *dacostae* Sowerby III, 1892; *dombeyanus* Pfeiffer, 1846; *dunkeri* Pfeiffer in Philippi, 1846; *elsteri* da Costa, 1901; *exoticus* da Costa, 1901; *expatriatus* Preston, 1909; *fabrefactus* Reeve, 1848; *fallax* Pfeiffer, 1853; *farrisi* Pfeiffer, 1858; *felix* Pfeiffer, 1862; *fenestratus* Pfeiffer, 1846; *flexilabris* Pfeiffer, 1853; *flexuosus* Pfeiffer, 1853; *fucatus* Reeve, 1849; *fusoides* d’Orbigny, 1835; *gealei* H. Adams, 1867; *geometricus* Pfeiffer, 1846; *gueinzii* Pfeiffer, 1857; *hidalgoi* da Costa, 1898; *humboldtii* Reeve, 1849; *hygrohylea* d’Orbigny, 1835; *inclinatus* Pfeiffer, 1862; *incognitus* da Costa, 1907; *jansoni* Martens, 1893; *josephus* Angas, 1878; *knorri* Pfeiffer in Philippi, 1846; *lamas* Higgins, 1868; *lattrei* Pfeiffer in Philippi, 1846; *laxostylus* Rolle, 1904; *lilacinus* Reeve, 1849; *linostoma* d’Orbigny, 1835; *lophoica* d’Orbigny, 1835; *lucidus* da Costa, 1898; *malleatus* da Costa, 1898; *marmarina* d’Orbigny, 1835; *murrinus* Reeve, 1848; *musivus* Pfeiffer, 1855; *napo* Angas, 1878; *notabilis* da Costa, 1906; *notatus* da Costa, 1906; *nystianus* Pfeiffer, 1853; *ochrocheilus* E.A. Smith, 1877; *orthostoma* E.A. Smith, 1877; *patricius* Reeve, 1849; *perenensis* da Costa, 1901; *pergracilis* Rolle, 1904; *phryne* Pfeiffer, 1863; *plicatoliratus* da Costa, 1898; *poecila* d’Orbigny, 1835; *ponsonbyi* da Costa, 1907; *praetextus* Reeve, 1849; *protractus* Pfeiffer, 1855; *pseudofusoides* da Costa, 1906; *pulcherrimus* H. Adams, 1867; *punctatus* da Costa, 1907; *quadrifasciatus* Angas, 1878; *recedens* Pfeiffer, 1864; *regularis* Fulton, 1905; *rosenbergi* da Costa, 1900; *rubrovariegatus* Higgins, 1868; *saccatus* Pfeiffer, 1855; *schmidti* Pfeiffer, 1854; *scitulus* Reeve, 1849; *scitus* H. Adams, 1867; *selli* Preston, 1909; *serratus* Pfeiffer, 1855; *smithii* da Costa, 1898; *solidus* Preston, 1907; *spadiceus* da Costa, 1906; *spectatus* Reeve, 1849; *strigatus* Sowerby I, 1833; *subhybridus* da Costa, 1906; *subinterruptus* Pfeiffer, 1853; *subventricosus* da Costa, 1901; *sykesi* da Costa, 1906; *tigrinus* da Costa, 1898; *vespertinus* Pfeiffer, 1858; *volsus* Fulton, 1907; *xanthostoma* d’Orbigny, 1835; *yungasensis* d’Orbigny, 1837; *zhorquinensis* Angas, 1879; *ziczac* da Costa, 1898; *zoographica* d’Orbigny, 1835.

###### Drymaeus (Mesembrinus)

Taxon classificationAnimaliaStylommatophoraBulimulidae

Albers, 1850

aestivus Pfeiffer, 1857; *amandus* Pfeiffer, 1855; *andicola* Pfeiffer, 1847; *apicepunctata* Preston, 1914; *apiculata* J.E. Gray, 1834; *attenuatus* Pfeiffer, 1853; *aureolus* Guppy, 1866; *aurifluus* Pfeiffer, 1857; *broadwayi* E.A. Smith, 1896; *bugabensis* Martens, 1893; *californicus* Reeve, 1848; *cancellata* da Costa, 1906; *castus* Pfeiffer, 1847; *championi* Martens, 1893; *citronellus* Angas, 1879; *columbiensis* Pfeiffer, 1856; *conicus* da Costa, 1907; *demotus* Reeve, 1850; *depictus* Reeve, 1849; *deshayesi* Pfeiffer, 1845; *discrepans* Sowerby I, 1833; *dubius* Pfeiffer, 1853; *dutaillyi* Pfeiffer, 1857; *electrum* Reeve, 1848; *erubescens* Pfeiffer, 1847; *feriatus* Reeve, 1850; *fidustus* Reeve, 1849; *flavidulus* E.A. Smith, 1877; *floridanus* Pfeiffer, 1857; *fuscobasis* E.A. Smith, 1877; *gabbi* Angas, 1879; *gruneri* Pfeiffer, 1846; *hachensis* Reeve, 1850; *hepatostomus* Pfeiffer, 1861; *hoffmanni* Martens, 1893; *hondurasanus* Pfeiffer, 1846; *hypozonus* Martens, 1893; *immaculatus* C.B. Adams in Reeve, 1850; *incarnatus* Pfeiffer, 1855; *inglorius* Reeve, 1848; *interruptus* Preston, 1909; *inusitatus* Fulton, 1900; *iodostylus* Pfeiffer, 1861; *jonasi* Pferiffer in Philippi, 1846; *keppelli* Pfeiffer, 1853; *koppeli* Sowerby III, 1892; *laetus* Reeve, 1849; *lascellianus* E.A. Smith, 1895; *lirinus* Morelet, 1851; *lividus* Reeve, 1850; *loxanus* Higgins, 1872; *loxensis* Pfeiffer, 1846; *lucidus* Reeve, 1848; *lusorius* Pfeiffer, 1855; *manupictus* Reeve, 1848; *meridanus* Pfeiffer, 1846; *monilifer* Reeve, 1848; *moricandi* Pfeiffer, 1847; *moritinctus* Martens, 1893; *mossi* E.A. Smith, 1896; *moussoni* Pfeiffer, 1853; *muliebris* Reeve, 1849; *nigrofasciatus* Pfeiffer in Philippi, 1846; *nitelinus* Reeve, 1849; *nitidus* Broderip in Broderip and Sowerby I 1832; *nubilus* Preston, 1903; *pallidus* Preston, 1909; *panamensis* Broderip in Broderip and Sowerby I 1832; *pervariabilis* Pfeiffer, 1853; *prestoni* da Costa, 1906; *primula* Reeve, 1848; *puellaris* Reeve, 1850; *rawsonis* H. Adams, 1873; *rectilinearis* Pfeiffer, 1855; *roseatus* Reeve, 1848; *signifer* Pfeiffer, 1855; *sisalensis* Morelet, 1849; *sowerbyi* Pfeiffer, 1847; *studeri* Pfeiffer, 1847; *subpellucidus* E.A. Smith, 1877; *sulcosus* Pfeiffer, 1841; *sulphureus* Pfeiffer, 1857; *tenuilabris* Pfeiffer, 1866; *translucens* Broderip in Broderip and Sowerby I 1832; *trimarianus* Martens, 1893; *trinitarius* E.A. Smith, 1986; *tristis* Pfeiffer, 1855; *tropicalis* Morelet, 1849; *umbraticus* Reeve, 1850; *varicosus* Pfeiffer, 1853; *vincentinus* Pfeiffer, 1846; *virginalis* Pfeiffer, 1856; *wintlei* Finch, 1929.

###### Kuschelenia (Kuschelenia)

Taxon classificationAnimaliaStylommatophoraBulimulidae

Hylton Scott, 1951

confusus Reeve, 1848; *culminea* d’Orbigny, 1835; *edwardsi* Morelt, 1863; *gayi* Pfeiffer, 1857; *jussieui* Pfeiffer, 1846; *lithoica* d’Orbigny, 1835; *thamnoica* d’Orbigny, 1835; *tupacii* d’Orbigny, 1835.

###### Kuschelenia (Bocourtia)

Taxon classificationAnimaliaStylommatophoraBulimulidae

Rochebrune, 1882 (comb. n.)

####### Remarks.

David Campbell (pers. commun.) kindly made us aware that the name *Vermiculatus* Breure, 1978 is preceded by *Bocourtia* Rochebrune, 1882. Rochebrune (1882: 117) described this genus from Thailand as a member of Lymnaeidae; the type species *Bocourtia lymnaeformis* Rochebrune, 1882 was subsequently designated by Hubendick (1951: 114), but Ancey (1906: 317) and Germain (1910: C.32) already recognised that this species was identical to *Bulimus anthisanensis* Pfeiffer, 1853. The name *Vermiculatu* s Breure, 1978 (type species *Bulinus bicolor* Sowerby I, 1835) is thus a subjective junior synonym of *Bocourtia* Rochebrune, 1882 (**syn. n.**). The following taxa are affected by this new classification (**comb. n.**):

*aequatorius* Pfeiffer, 1853; *anthisanensis* Pfeiffer, 1853; *aquilus* Reeve, 1848; *badius* Sowerby I, 1835; *bicolor* Sowerby I, 1835; *caliginosus* Reeve, 1849; *coagulatus* Reeve, 1849; *cotopaxiensis* Pfeiffer, 1853; *filaris* Pfeiffer, 1853; *nucinus* Reeve, 1850; *ochraceus* Morelet, 1863; *peaki* Breure, 1978; *petiti* Pfeiffer, 1846; *polymorpha* d’Orbigny, 1835; *purpuratus* Reeve, 1849; *quechuarum* Crawford, 1939; *subfasciatus* Pfeiffer, 1853.

###### Naesiotus

Taxon classificationAnimaliaStylommatophoraBulimulidae

Albers, 1850 sensu Breure 1979

achatellinus Forbes, 1850; *albemarlensis* Dall, 1917; *apertus* Pfeiffer, 1855; *catlowiae* Pfeiffer, 1853; *chamayensis* Weyrauch, 1967; *chemnitzioides* Forbes, 1850; *cinereus* Reeve, 1849; *crepundia* d’Orbigny, 1835; *curtus* Reibisch, 1892; *darwini* Pfeiffer, 1846; *dentritis* Morelet, 1863; *durangoanus* Martens 1893; *eschariferus* Sowerby I, 1838; *exornatus* Reeve, 1849; *fernandezae* Weyrauch, 1958; *fontainii* d’Orbigny, 1838; *fourmiersi* d’Orbigny, 1837; *galapaganus* Pfeiffer, 1855; *irregularis* Pfeiffer, 1848; *jacobi* Sowerby I, 1833; *lycodus* Dall, 1917; *montivaga* d’Orbigny, 1835; *munsterii* d’Orbigny, 1837; *nucula* Pfeiffer, 1853; *nux* Broderip, 1832; *orbignyi* Pfeiffer, 1846; *pallidus* Reibisch, 1892; *paziana* d’Orbigny, 1835; *perspectivus* Pfeiffer, 1846; *phlegonis* Dall and Ochsner, 1928; *quitensis* Pfeiffer, 1848; *rimatus* Pfeiffer, 1847; *rivasii* d’Orbigny, 1837; *rocayana* d’Orbigny, 1835; *rugiferus* Sowerby I, 1833; *rugulosus* Sowerby I, 1833; *sculpturatus* Pfeiffer, 1846; *sugillatus* Pfeiffer, 1857; *terebra* Reibisch, 1892; *trichoda* d’Orbigny, 1835; *unifasciatus* Sowerby I, 1833; *ustulatus* Sowerby I, 1833; *ventrosus* Reibisch, 1892; *verrucosus* Pfeiffer, 1855; *wolfi* Reibisch, 1892.

###### Neopetraeus

Taxon classificationAnimaliaStylommatophoraBulimulidae

Martens, 1885

altoperuvianus Reeve, 1849; *atahualpa* Dohrn, 1863; *binneyanus* Pfeiffer, 1857; *cora* d’Orbigny, 1835; *decussatus* Reeve, 1849; *excoriatus* Pfeiffer, 1855; *lobbii* Reeve, 1849; *myristicus* Reeve, 1849; *patasensis* Pfeiffer, 1858; *platystomus* Pfeiffer, 1858; *ptychostylus* Pfeiffer, 1858.

###### Newboldius

Taxon classificationAnimaliaStylommatophoraBulimulidae

Pilsbry, 1932

crichtoni Broderip, 1836; *illustris* Rolle, 1905.

###### Protoglyptus

Taxon classificationAnimaliaStylommatophoraBulimulidae

Pilsbry, 1897

martinicensis Pfeiffer, 1846; *pilosus* Guppy, 1871; *sanctaeluciae* E.A. Smith, 1889.

###### Rabdotus

Taxon classificationAnimaliaStylommatophoraBulimulidae

Albers, 1850

juarezi Pfeiffer, 1866; *liquabilis* Reeve, 1848; *ragsdalei* Pilsbry, 1890; *schiedeanus* Pfeiffer, 1841.

###### Scutalus

Taxon classificationAnimaliaStylommatophoraBulimulidae

Albers, 1850

baroni (*Helix*) Fulton, 1896; *baroni* (*Bulimulus*) Fulton, 1897; *chiletensis* Weyrauch, 1967; *cretaceus* Pfeiffer, 1855; *grandiventris* Weyrauch, 1960; *latecolumellaris* Preston, 1909; *proteus* Broderip in Broderip and Sowerby I 1832; *versicolor* Broderip in Broderip and Sowerby I 1832.

###### Stenostylus

Taxon classificationAnimaliaStylommatophoraBulimulidae

Pilsbry, 1898

meleagris Pfeiffer, 1853; *nigrolimbatus* Pfeiffer, 1853.

###### Nomina inquirenda

*clarus* Pfeiffer, 1857; *dukinfieldi* Melvill, 1900; *gelidus* Reeve, 1849; *nivalis* d’Orbigny, 1835; *pallens* Reeve, 1849; *sowerbyi* Pfeiffer, 1847.

p. 17: ***Bulimus amandus* Pfeiffer, 1855:** registration number should read 1975457.

p. 49: ***Drymaeus conicus* da Costa, 1907:** registration number for paralectotype should read 1907.11.21.32.

p. 69: ***Bulinus eschariferus* Sowerby I, 1838:** Type material should read NHMUK 1975173, five possible syntypes (Cuming coll.).

p. 101: ***Bulimus jussieui* Pfeiffer, 1846:** Type material should read NHMUK 1975170, lectotype and one paralectotype (Cuming coll.).

p. 180: ***Bulimus sisalensis* Morelet, 1849:** Remarks should read Breure (1979: 123) erroneously mentioned “LT BMNH 1893.2.4.1655”; as the lectotype was already selected in Breure 1975b: 1152, this specimen is now one of the paralectotypes. The current systematic position follows Thompson (2011: 120).

p. 238: ***Bulimus gruneri* Pfeiffer, 1846:** figured specimen is not the lectotype but of paralectotype NHMUK 20100563/1.

p. 259, Fig. 45D–F: the figured specimen is not the lectotype but one of the pralectotypes.

#### VIII. Taxa excluded from the Orthalicoidea.

This is additional to the taxa excluded in the previous papers (Breure and Ablett 2011, 2012, 2014).

*Bulimus cucullus*Morelet 1849: 9.—Now placed in the family Succineidae.

*Bulimulus glandiniformis*Sowerby III 1892: 297, pl. 23 figs 13–14.— Now placed in the family Subulinidae.

## Figures and Tables

**Figure 1.**
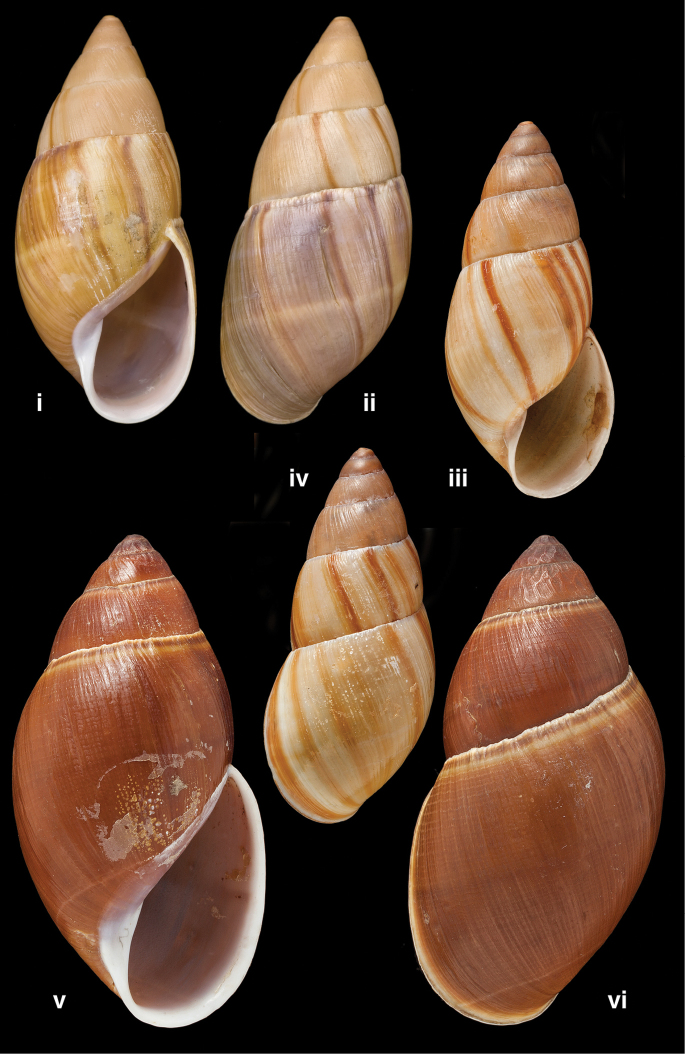
*Thaumastus* species. **i–ii** *Thaumastus taunaisii* (Férussac, 1822), lectotype of *Bulimus achilles* Pfeiffer, 1853 NHMUK 1975268 (H = 58.0) **iii–iv** *Thaumastus largillierti* (Philippi, 1842), holotype *Bulimus consimilis* Reeve, 1848 NHMUK 20030189 (H = 52.9) **v–vi** *Thaumastus foveolatus* (Reeve, 1849), lectotype NHMUK 1975275 (H = 71.5).

**Figure 3.**
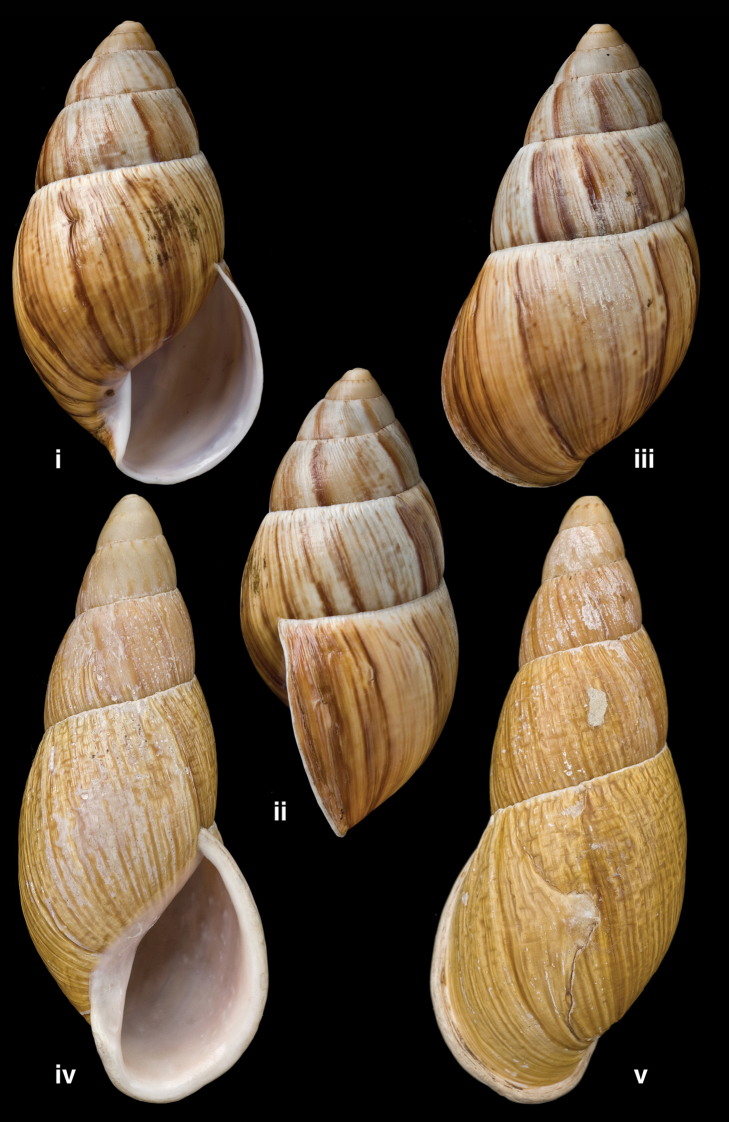
*Thaumastus* species. **i–iii** *Thaumastus hartwegi* (Pfeiffer in Philippi, 1846), syntype NHMUK 1975126 (H = 57.0) **iv–v** *Thaumastus buckleyi* (Higgins, 1872), syntype NHMUK 1872.5.22.6 (H = 92.0).

**Figure 4.**
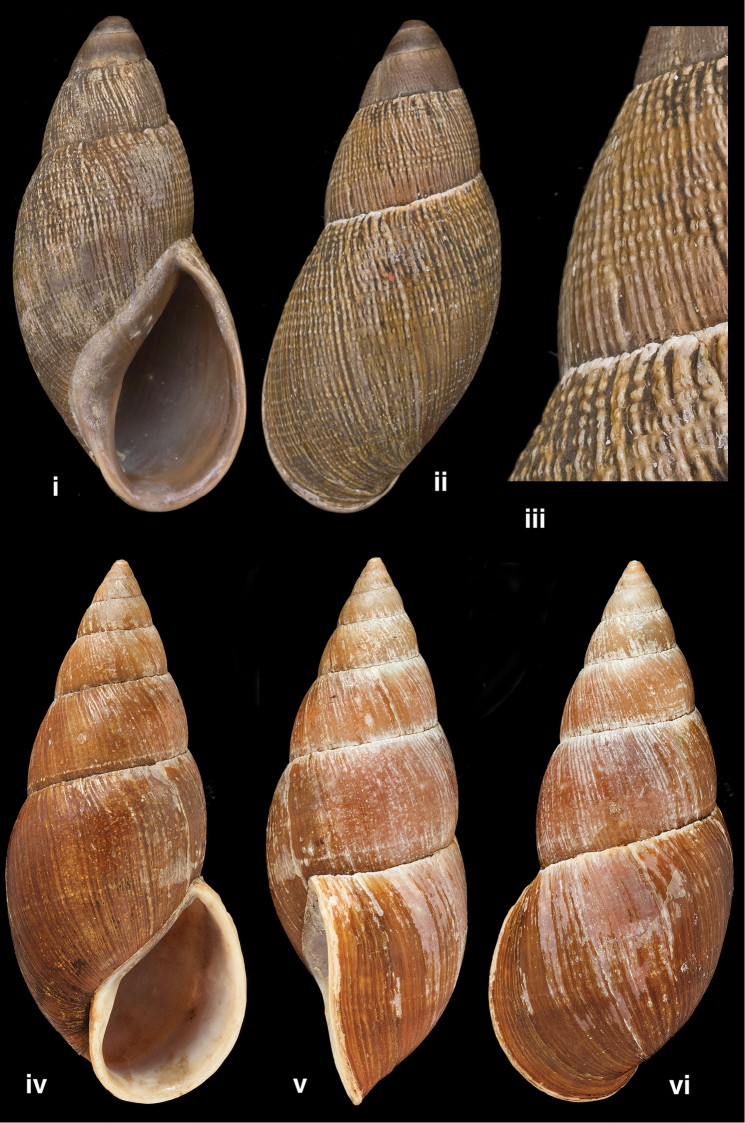
*Thaumastus* species. **i–iii** *Thaumastus insolitus* (Preston, 1909), holotype NHMUK 1947.3.11.1 (H = 70.4) **iv–vi** *Thaumastus inca* (d’Orbigny, 1835), lectotype NHMUK 1854.12.4.116 (H = 75.4).

**Figure 6.**
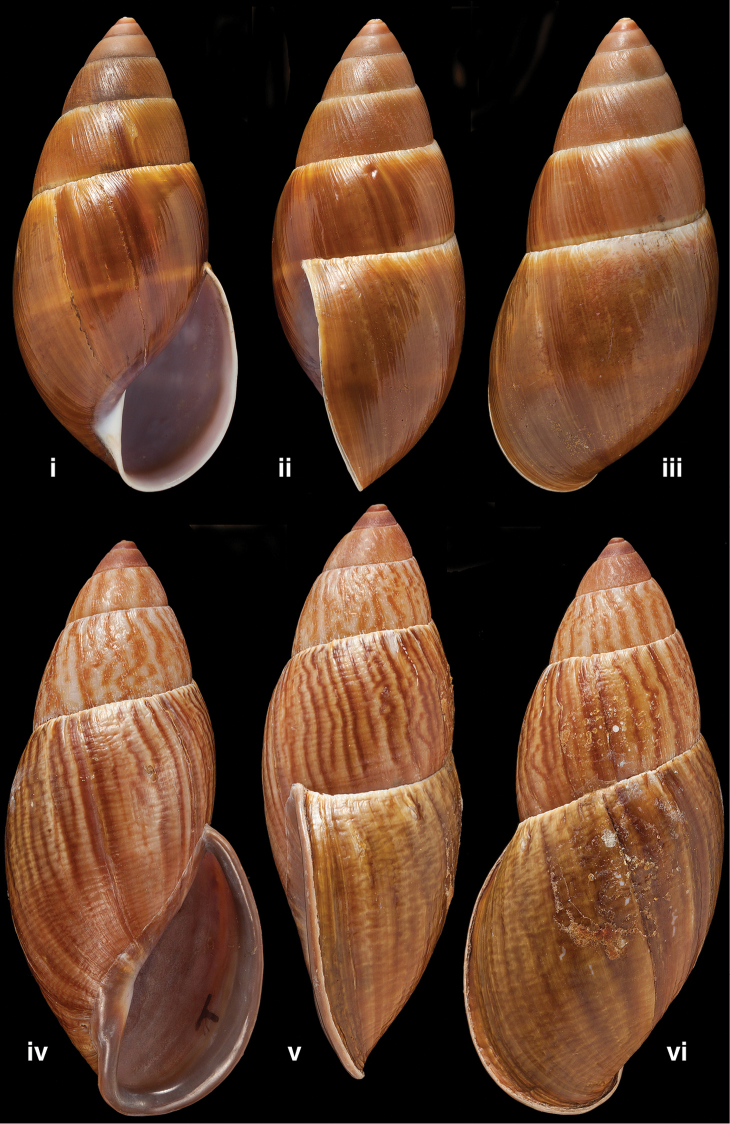
*Thaumastus* species. **i–iii** *Thaumastus magnificus* (Grateloup, 1839), lectotype NHMUK 1907.11.22.24 (H = 78.0) **iv–vi** *Thaumastus plumbeus* (Pfeiffer, 1855), lectotype NHMUK 1975130 (H = 93.0).

**Figure 8.**
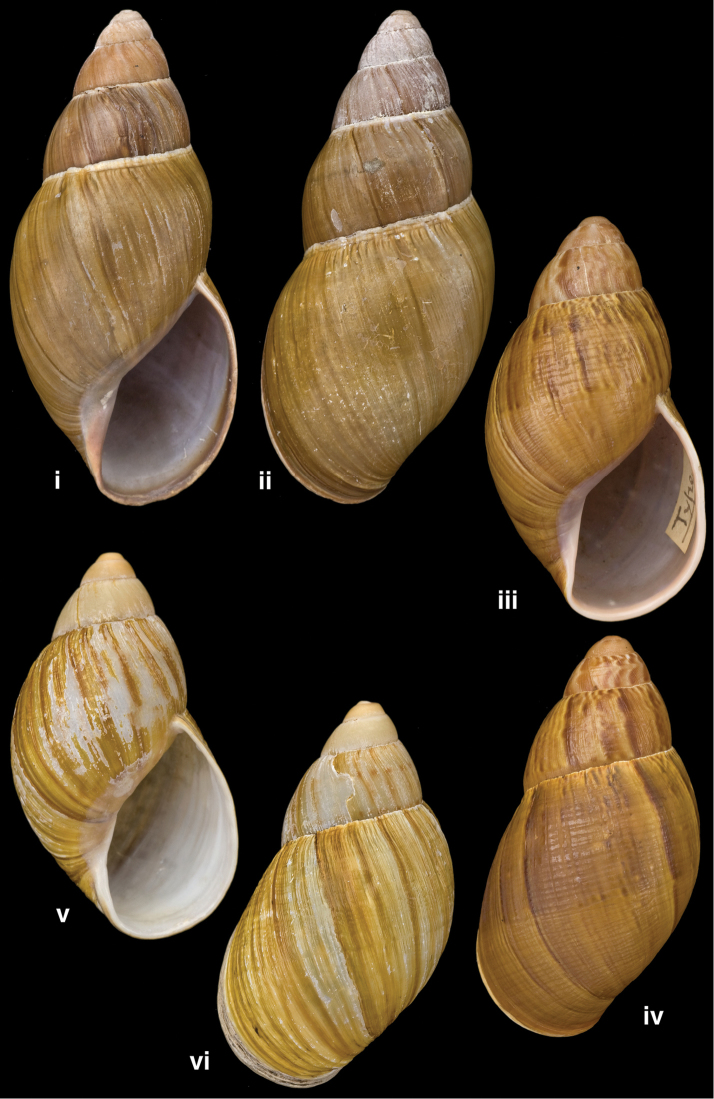
*Kara* species. **i–ii** *Kara thompsonii* (Pfeiffer, 1848), lectotype NHMUK 1975464 (H = 71.0) **iii–iv** *Kara indentatus* (da Costa, 1901), lectotype NHMUK 1907.11.21.115 (H = 44.0) **v–vi** *Kara yanamensis* (Morelet, 1863), paralectotype NHMUK 1893.2.4.167 (H = 48.6).

**Figure 9.**
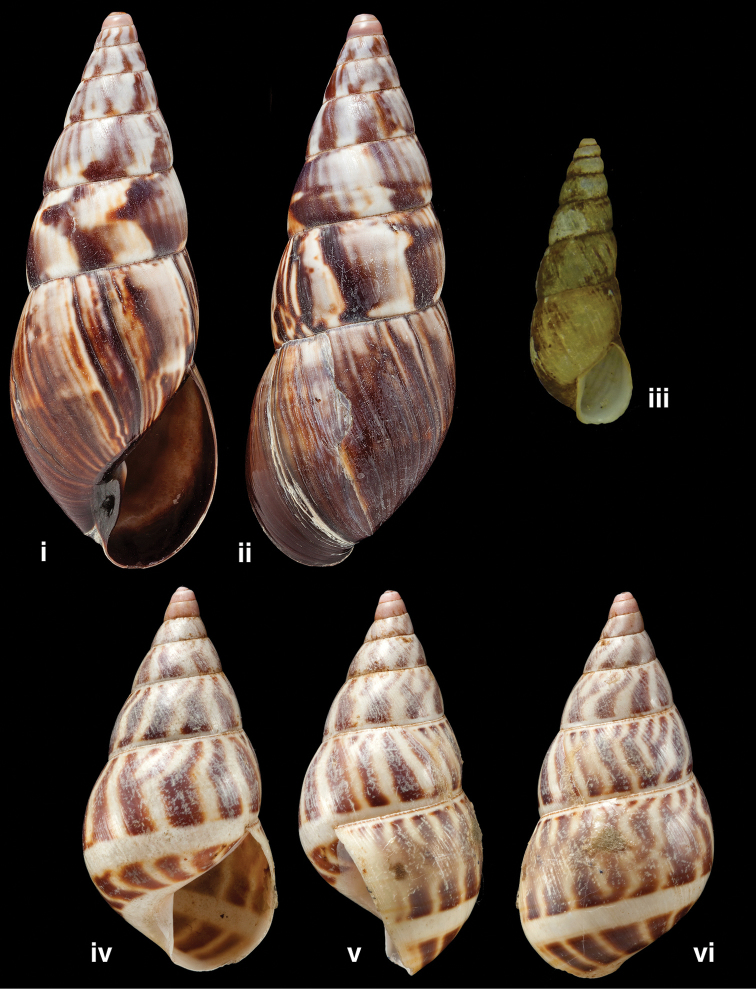
**i–ii** *Corona pfeifferi gracilis* E.A. Smith, 1902, holotype NHMUK 1902.5.27.4 (H = 67.3) **iii** ?*Naesiotus dejectus* (Fulton, 1907), lectotype NHMUK 1907.5.3.163 (H = 29.2) **iv–vi** *Liguus murreus* (Reeve, 1849), syntype NHMUK 1975482 (H = 38.6).

**Figure 10.**
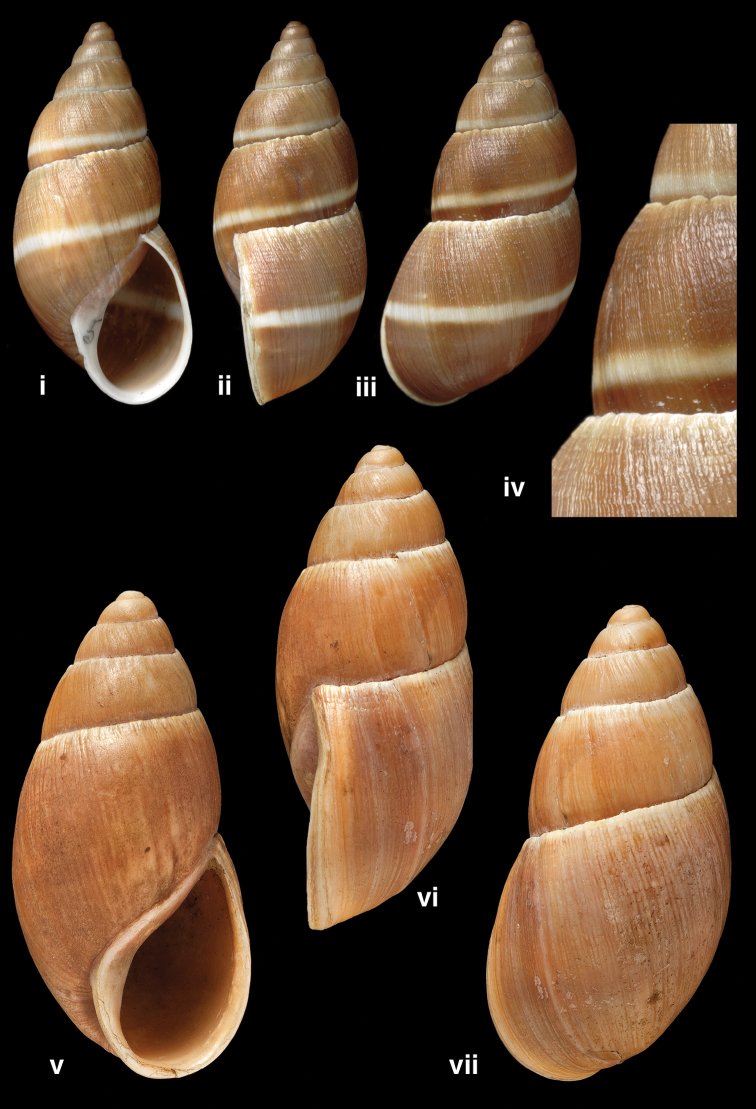
*Scholvienia* species. **i–iv** *Scholvienia alutaceus* (Reeve, 1849), lectotype NHMUK 1975148 (H = 35.5) **v–vii** *Scholvienia brephoides* (d’Orbigny, 1835), lectotype NHMUK 1854.12.4.117 (H = 51.9).

**Figure 12.**
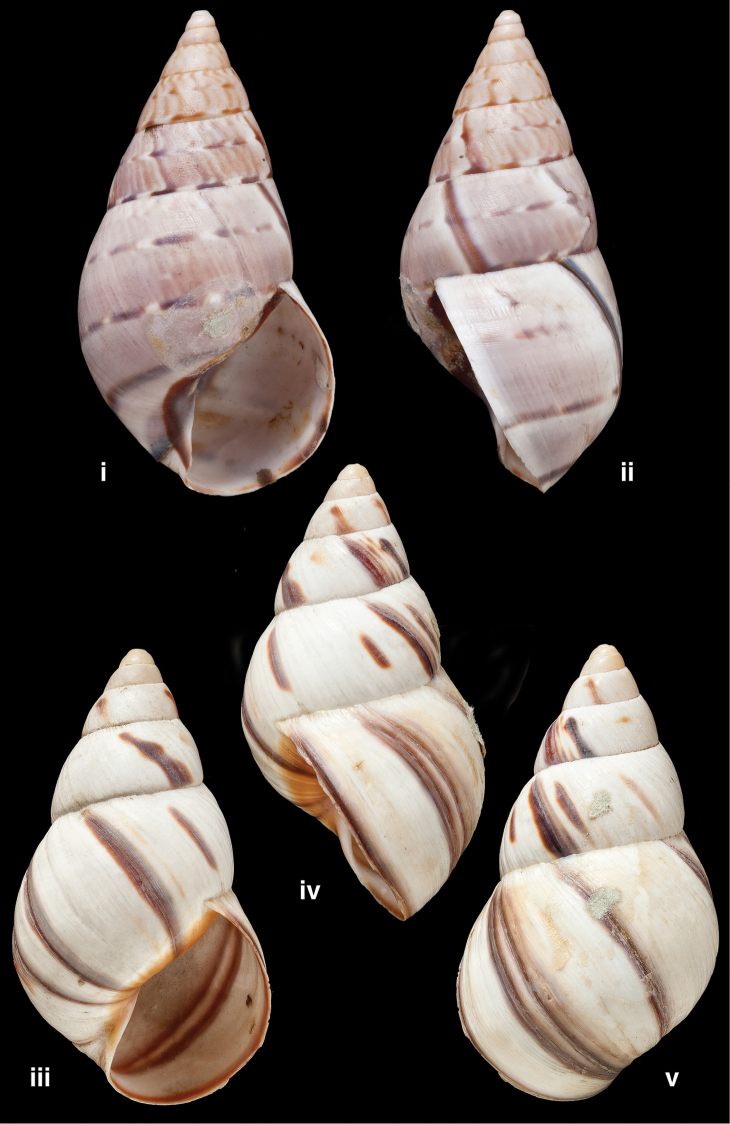
*Orthalicus* species. **i–ii** *Orthalicus bifulguratus* (Reeve, 1849), lectotype NHMUK 20140082 (H = 56.9) **iii–v** *Orthalicus boucardi* (Pfeiffer, 1860), syntype NHMUK 20140081 (H = 54.8).

**Figure 16.**
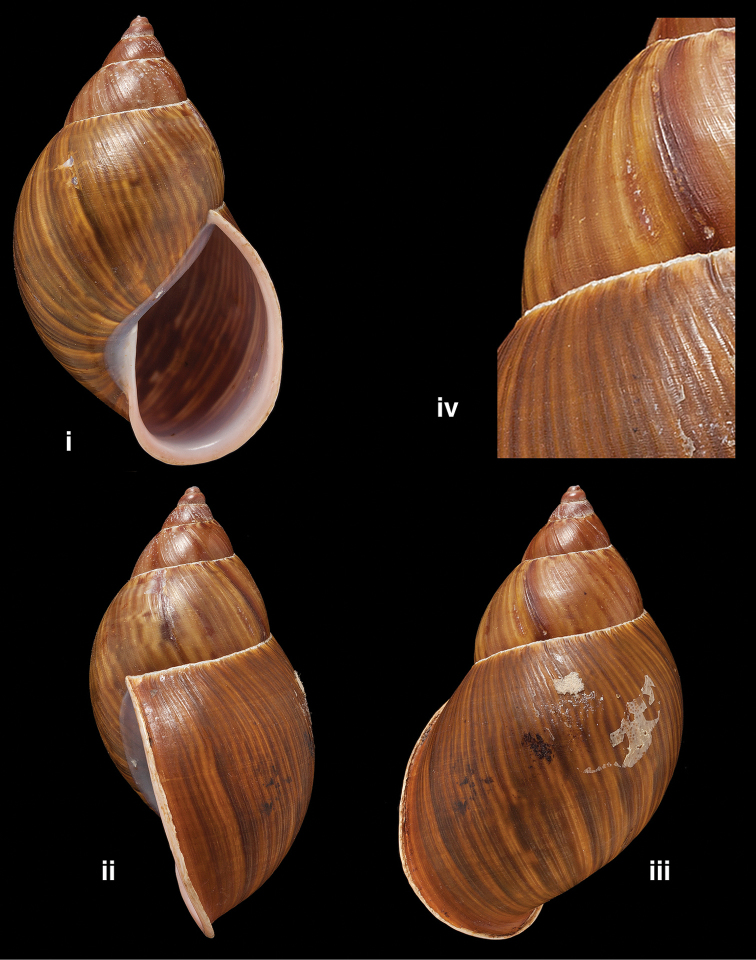
**i–iv** Porphyrobaphe (Oxyorthalicus) subirroratus (da Costa, 1898), lectotype NHMUK 1907.11.21.114 (H = 62.6).

**Figure L1.**
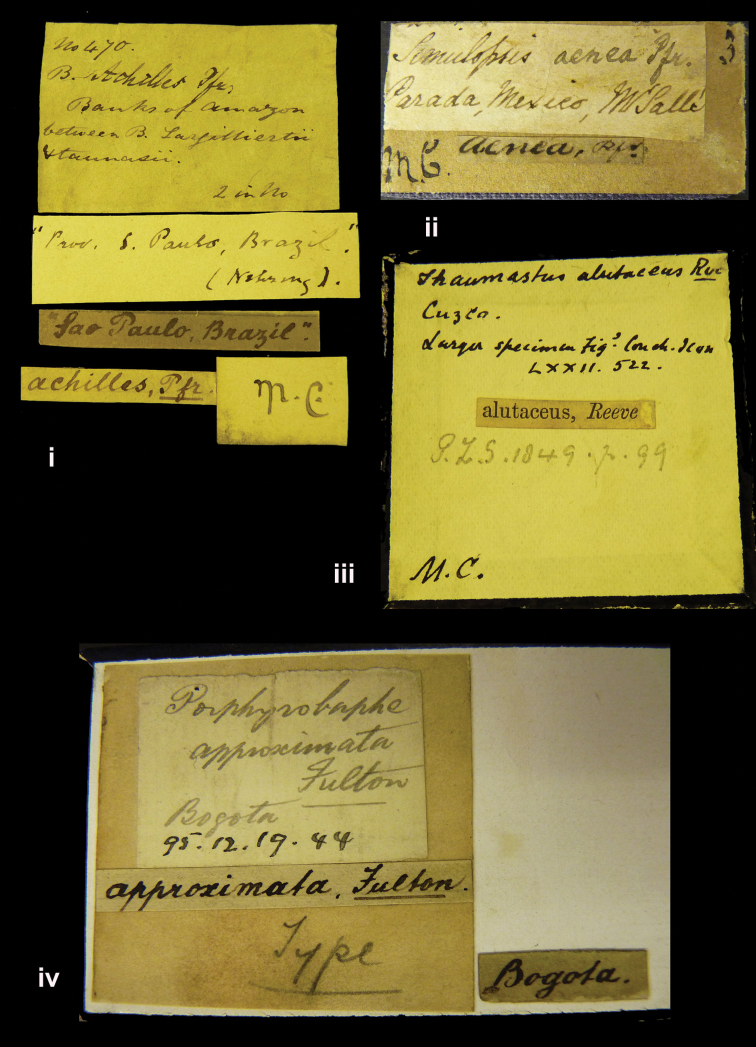
**i** *Bulimus achilles* Pfeiffer, 1853 **ii** *Simpulopsis aenea* Pfeiffer, 1861 **iii** *Bulimus alutaceus* Reeve, 1849 **iv** *Porphyrobaphe approximata* Fulton, 1896.

**Figure L2.**
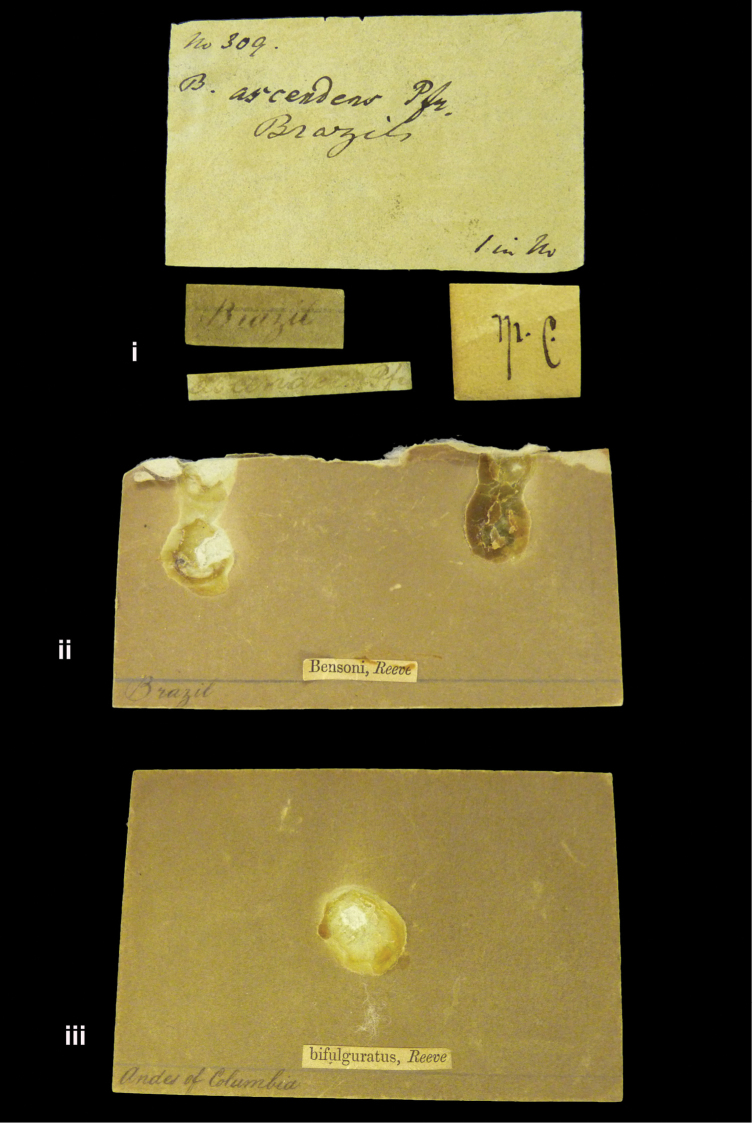
**i** *Bulimus ascendens* Pfeiffer, 1853 **ii** *Bulimus bensoni* Reeve, 1849 **iii** *Bulimus bifulguratus* Reeve, 1849.

**Figure L3.**
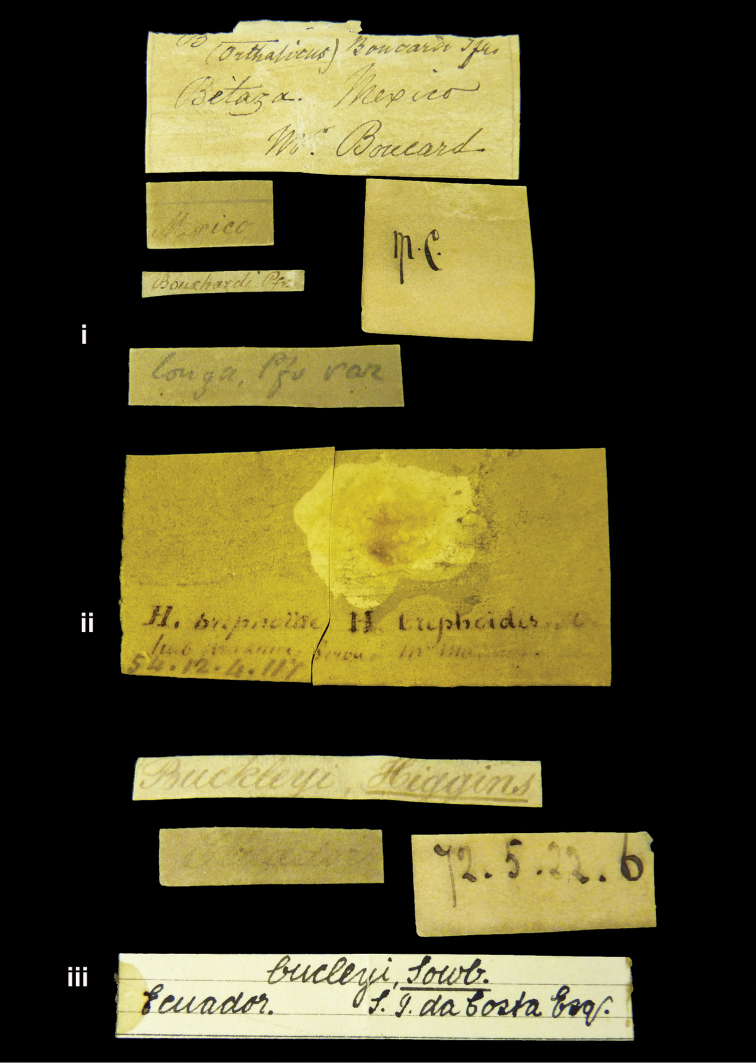
**i** *Orthalicus boucardi* Pfeiffer, 1860 **ii** *Helix brephoides* d’Orbigny, 1835 **iii** Orthalicus (Porphyrobaphe) buckleyi Higgins, 1872.

**Figure L4.**
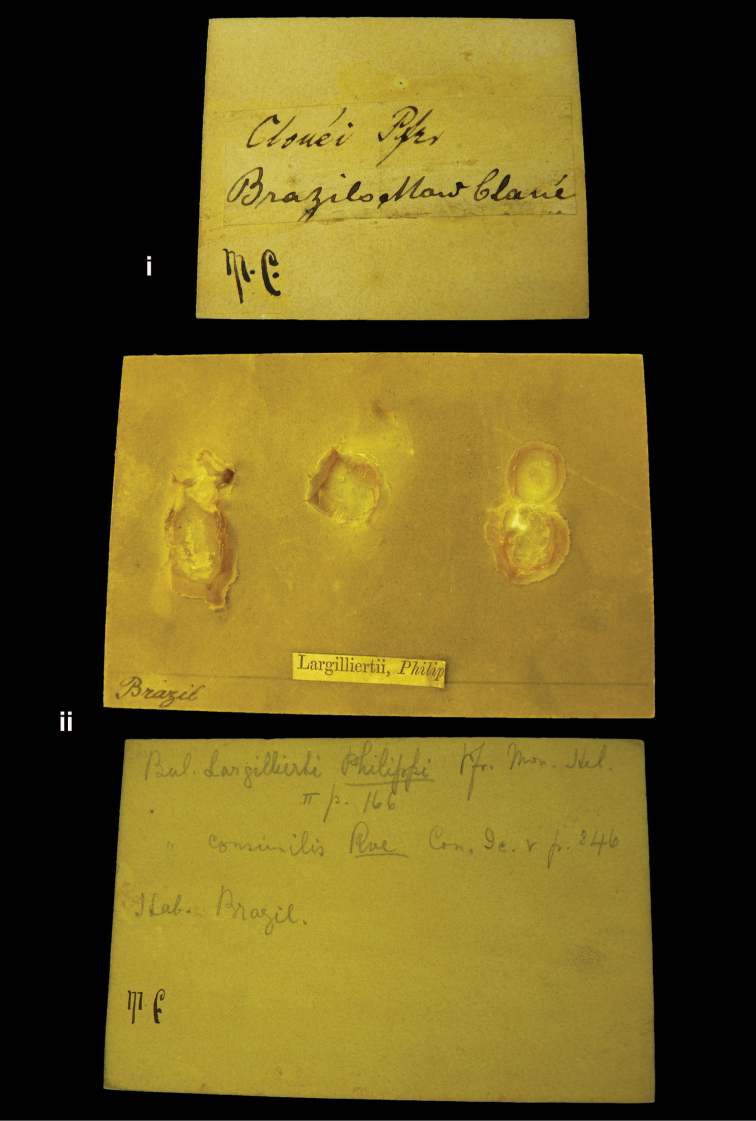
**i** *Bulimus clouei* Pfeiffer, 1857 **ii** *Bulimus consimilis* Reeve, 1848.

**Figure L5.**
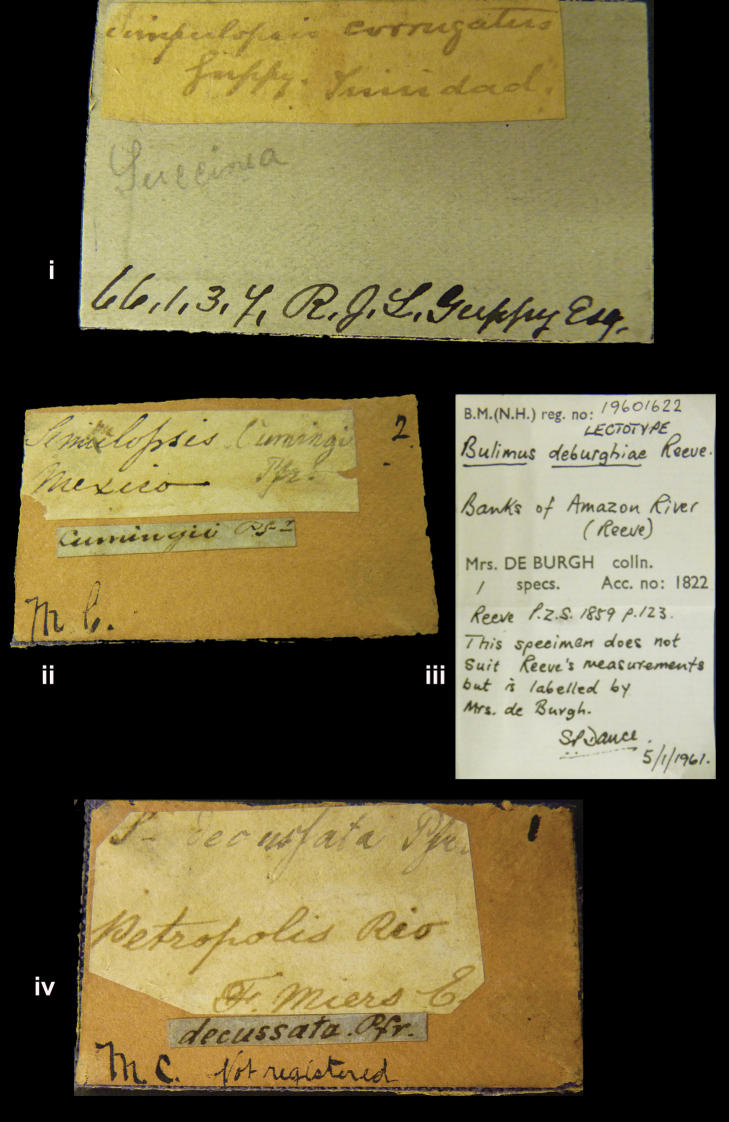
**i** *Simpulopsis corrugatus* Guppy, 1866 **ii** *Simpulopsis cumingi* Pfeiffer, 1861 **iii** *Bulimus deburghiae* Reeve, 1859 **iv** *Simpulopsis decussata* Pfeiffer, 1857.

**Figure L6.**
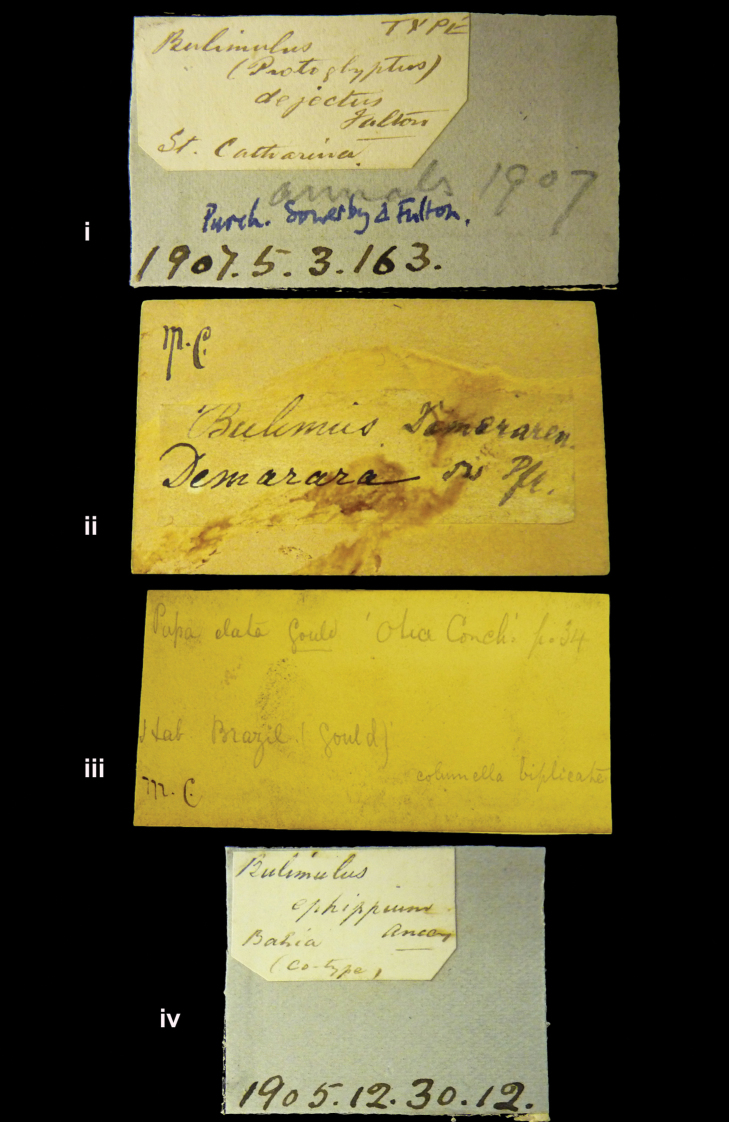
**i** Bulimulus (Protoglyptus) dejectus Fulton, 1907 **ii** *Bulimus demerarensis* Pfeiffer, 1861 **iii** Pupa (Megaspira) elata Gould, 1847 **iv** *Bulimulus ephippium* Ancey, 1904.

**Figure L7.**
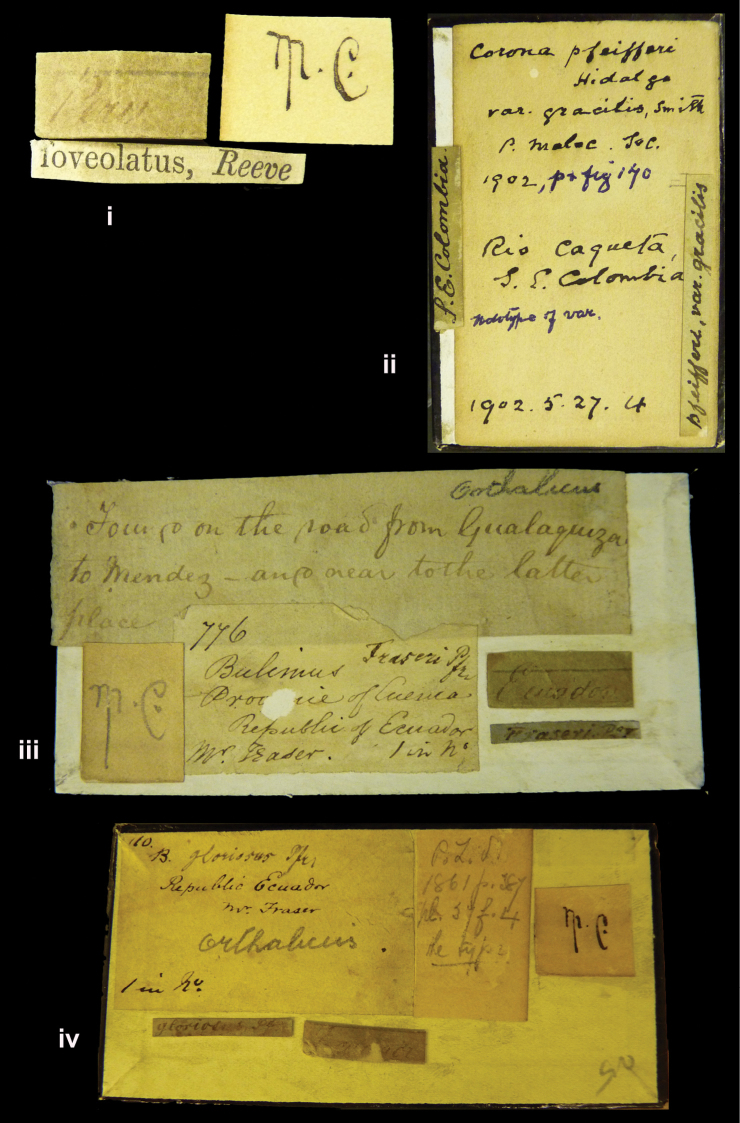
**i** *Bulimus foveolatus* Reeve, 1849 **ii** *Corona pfeifferi gracilis* E.A. Smith, 1902 **iii** *Bulimus fraseri* Pfeiffer, 1858 **iv** *Bulimus gloriosus* Pfeiffer, 1862.

**Figure L8.**
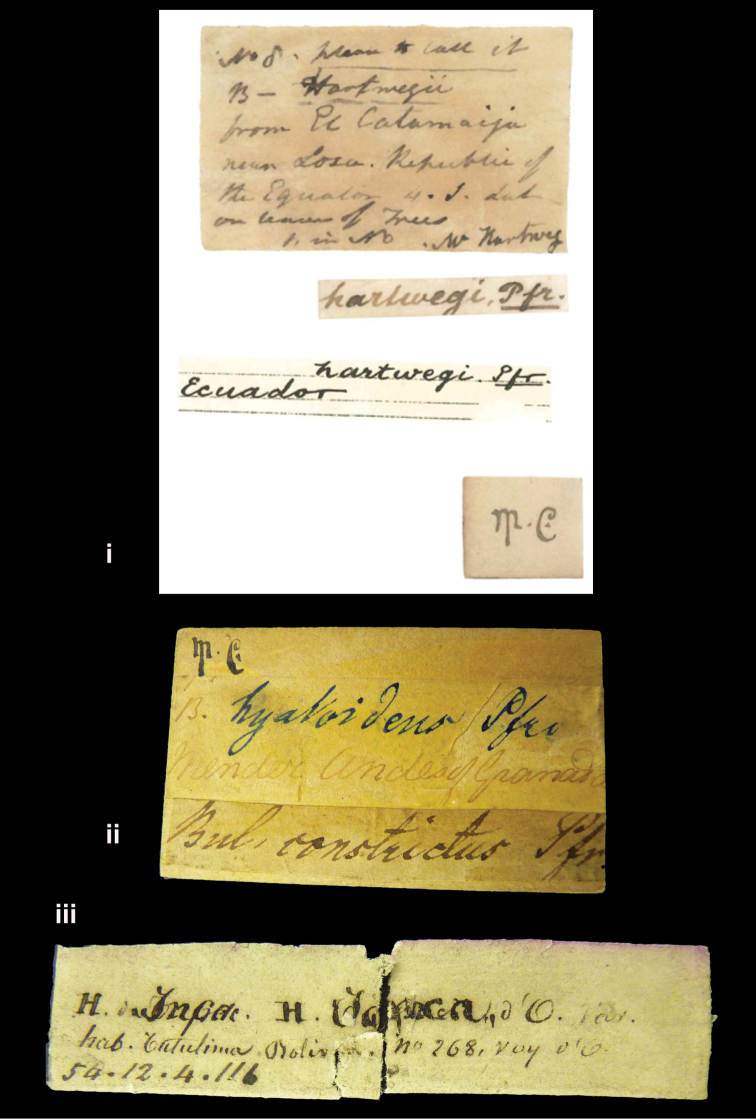
**i** *Bulimus hartwegi* Pfeiffer in Philippi, 1846 **ii** *Bulimus hyaloideus* Pfeiffer, 1855 **iii** *Helix inca* d’Orbigny, 1835.

**Figure L9.**
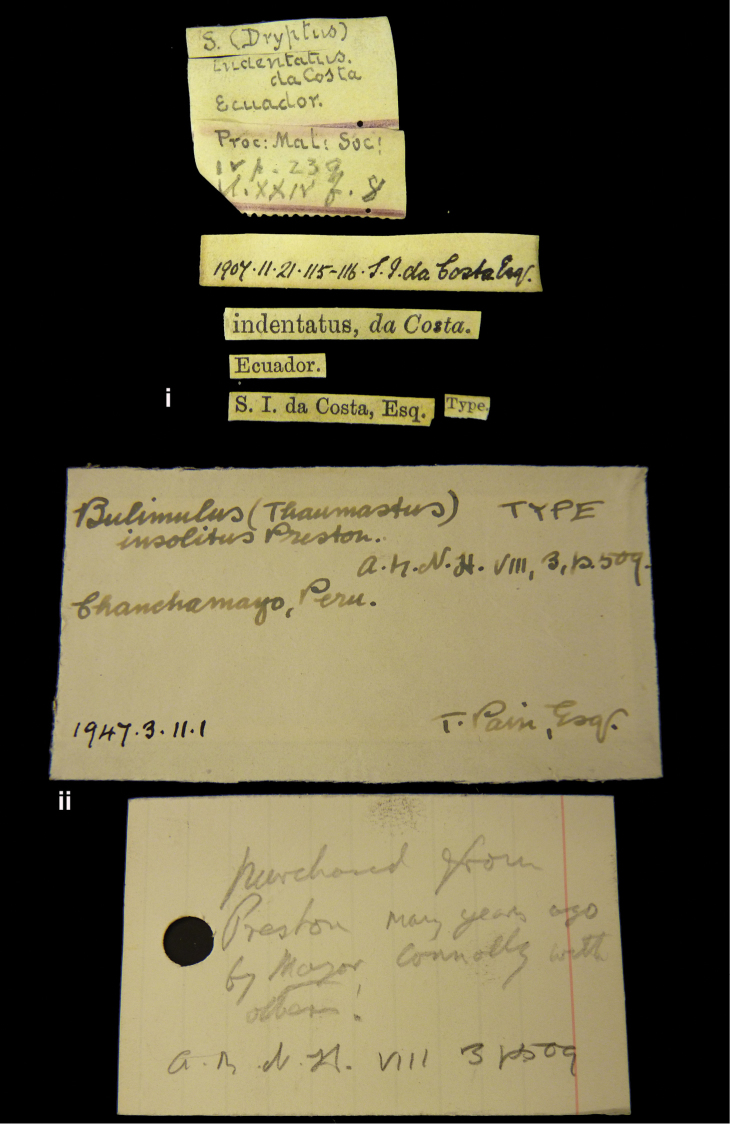
**i** Strophocheilus (Dryptus) indentatus da Costa, 1901 **ii** Bulimus (Thaumastus) insolitus Preston, 1909.

**Figure L10.**
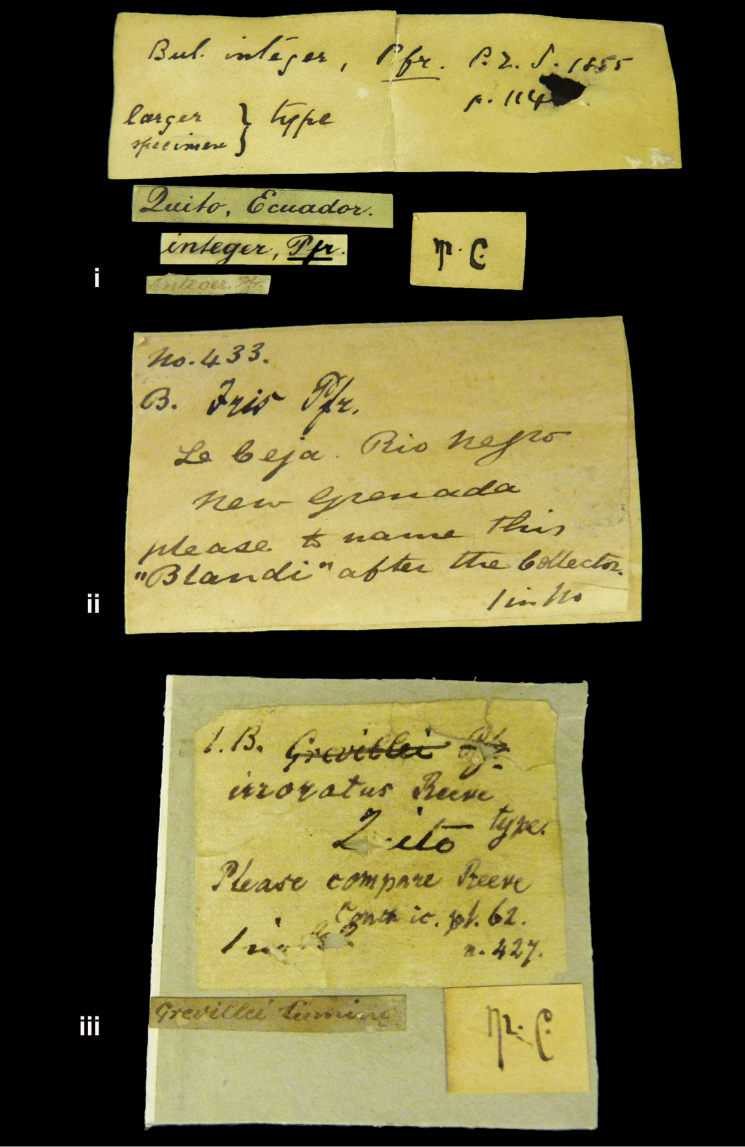
**i** *Bulimus integer* Pfeiffer, 185**5 ii** *Bulimus iris* Pfeiffer, 1853 **iii** *Bulimus irroratus* Reeve, 1849.

**Figure L11.**
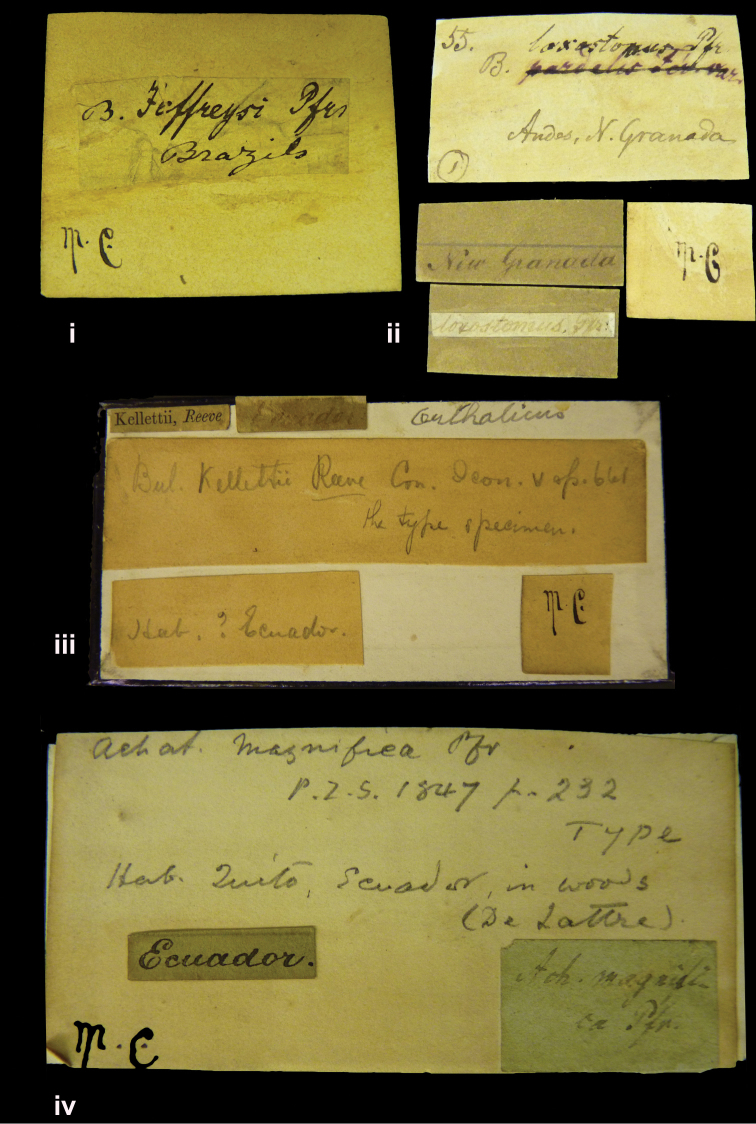
**i** *Bulimus jeffreysi* Pfeiffer, 1852 **ii** *Bulimus kelletti* Reeve, 1850 **iii** *Bulimus loxostomus* Pfeiffer, 1853 **iv** *Achatina magnifica* Pfeiffer, 1848.

**Figure L12.**
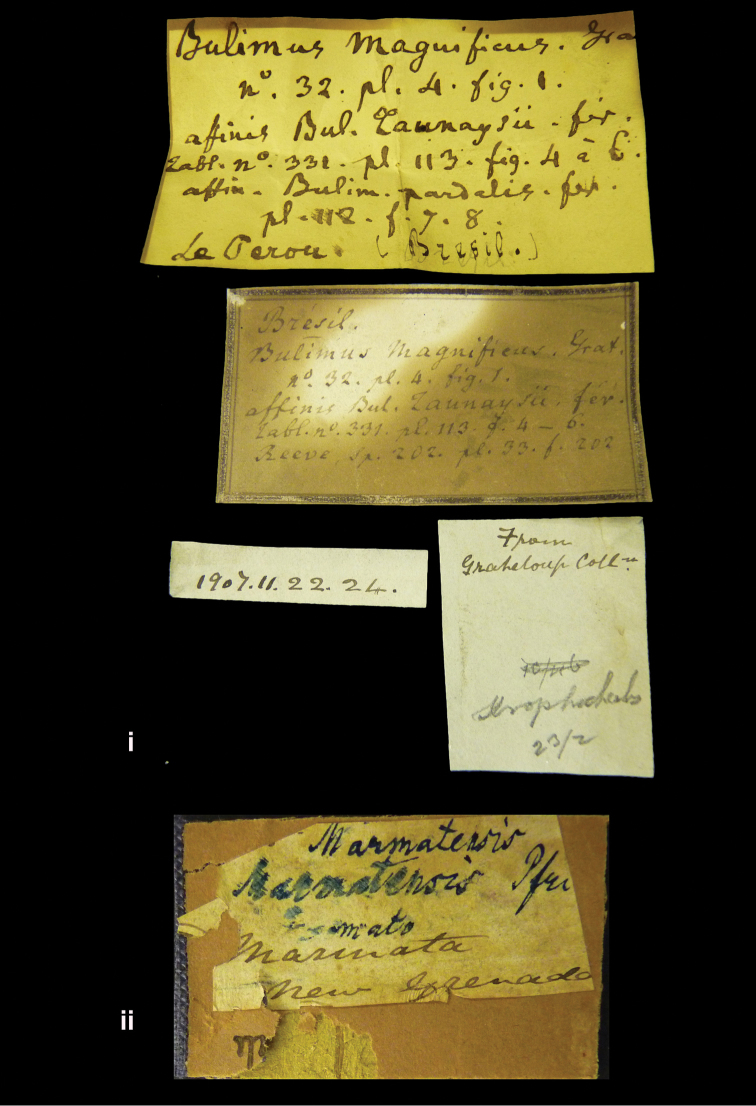
**i** *Bulimus magnificus* Grateloup, 1839 **ii** *Bulimus marmatensis* Pfeiffer, 1855.

**Figure L13.**
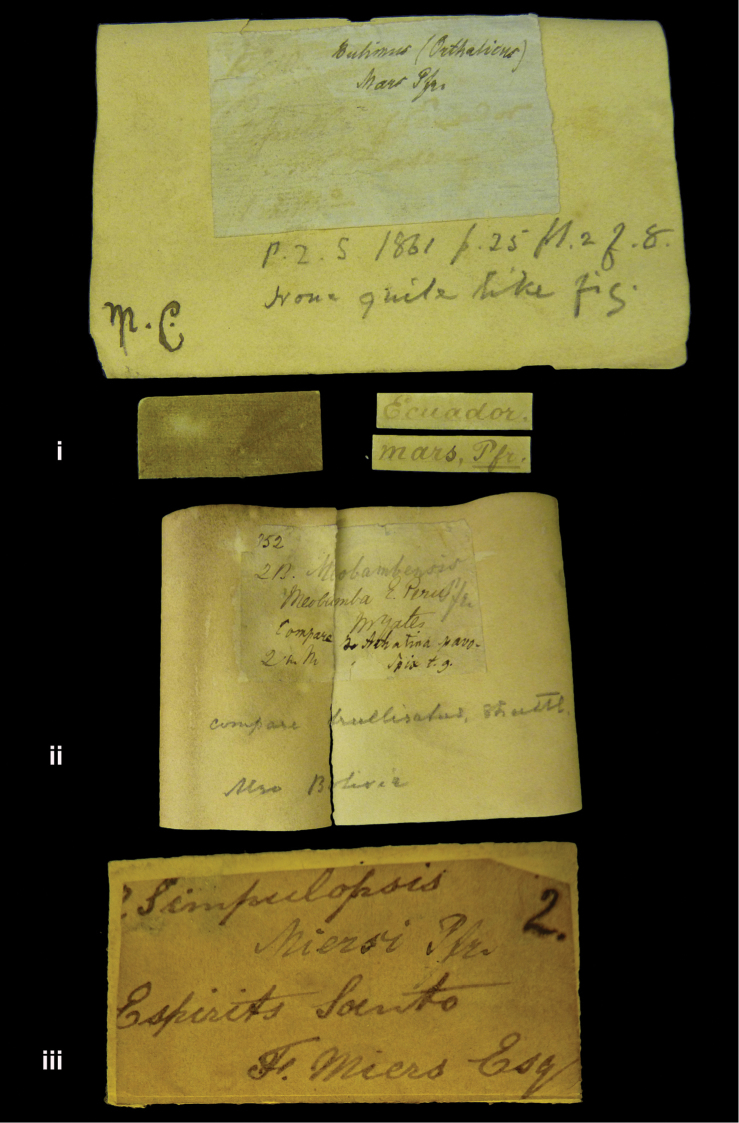
**i** *Orthalicus mars* Pfeiffer, 1861 **ii** *Bulimus meobambensis* Pfeiffer, 1855 **iii** *Simpulopsis miersi* Pfeiffer, 1857.

**Figure L14.**
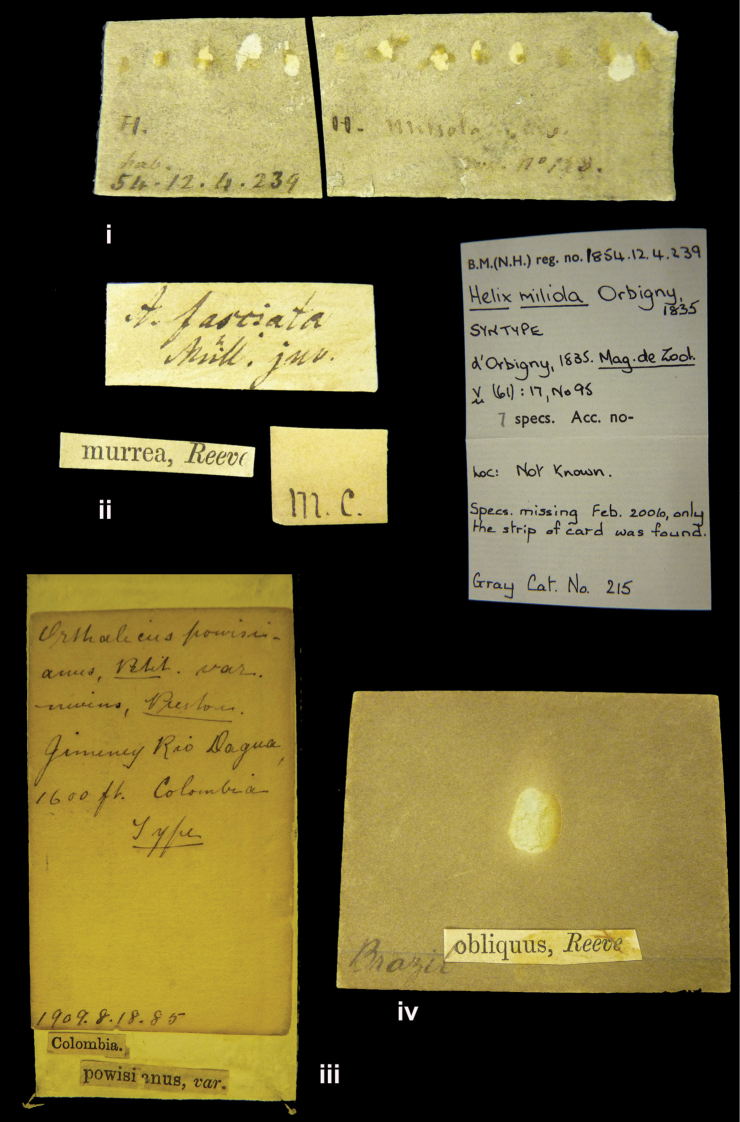
**i** *Helix miliola* d’Orbigny, 1835 **ii** *Achatina murrea* Reeve, 1849 **iii** Orthalicus powissianus var. niveus Preston, 1909 **iv** *Bulimus obliquus* Reeve, 1849.

**Figure L15.**
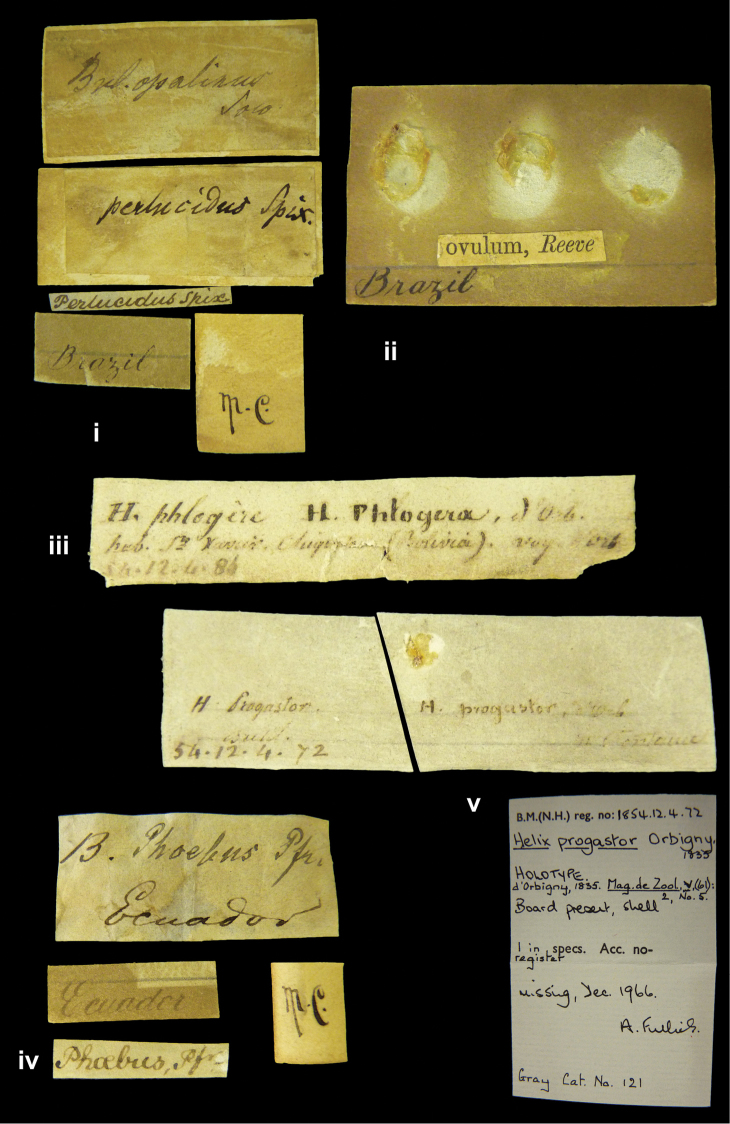
**i** *Bulinus opalinus* Sowerby I, 1833 **ii** *Bulimus ovulum* Reeve, 1849 **iii** *Helix phlogera* d’Orbigny, 1835 **iv** *Bulimus phoebus* Pfeiffer, 1863 **v** *Helix progastor* d’Orbigny, 1835.

**Figure L16.**
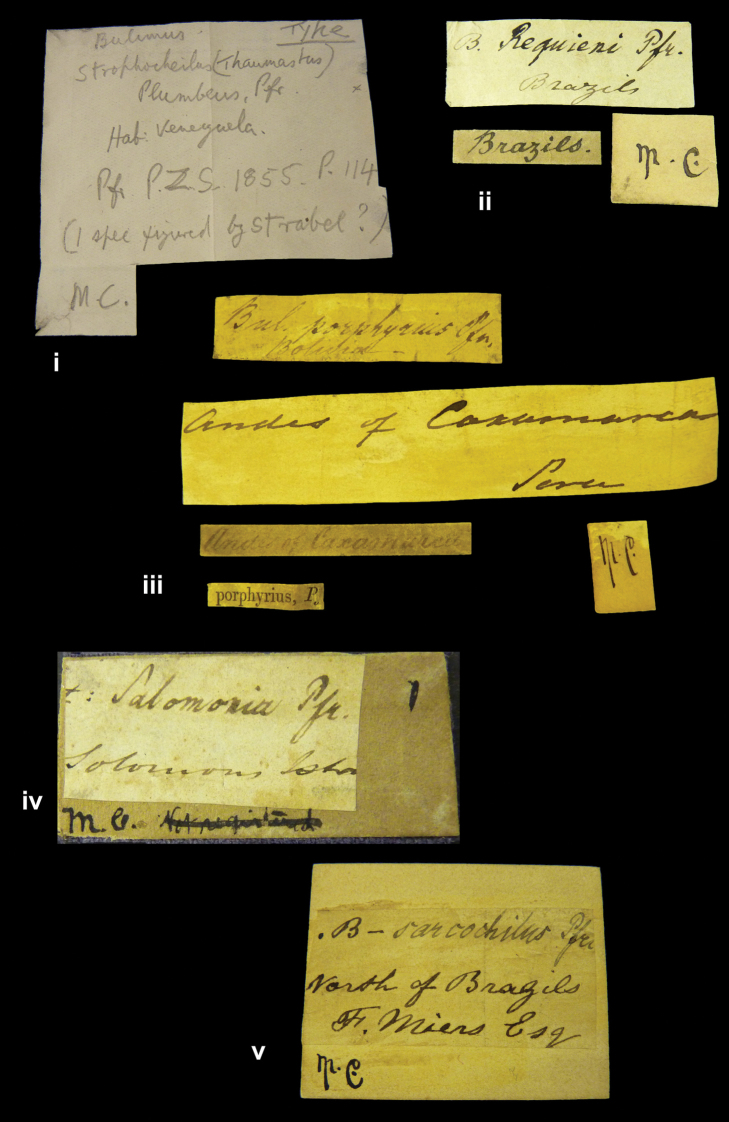
**i** *Bulimus plumbeus* Pfeiffer, 1855 **ii** *Bulimus requieni* Pfeiffer, 1853 **iii** *Bulimus porphyrius* Pfeiffer, 1847 **iv** *Vitrina salomonia* Pfeiffer, 1853 **v** *Bulimus sarcochilus* Pfeiffer, 1857.

**Figure L17.**
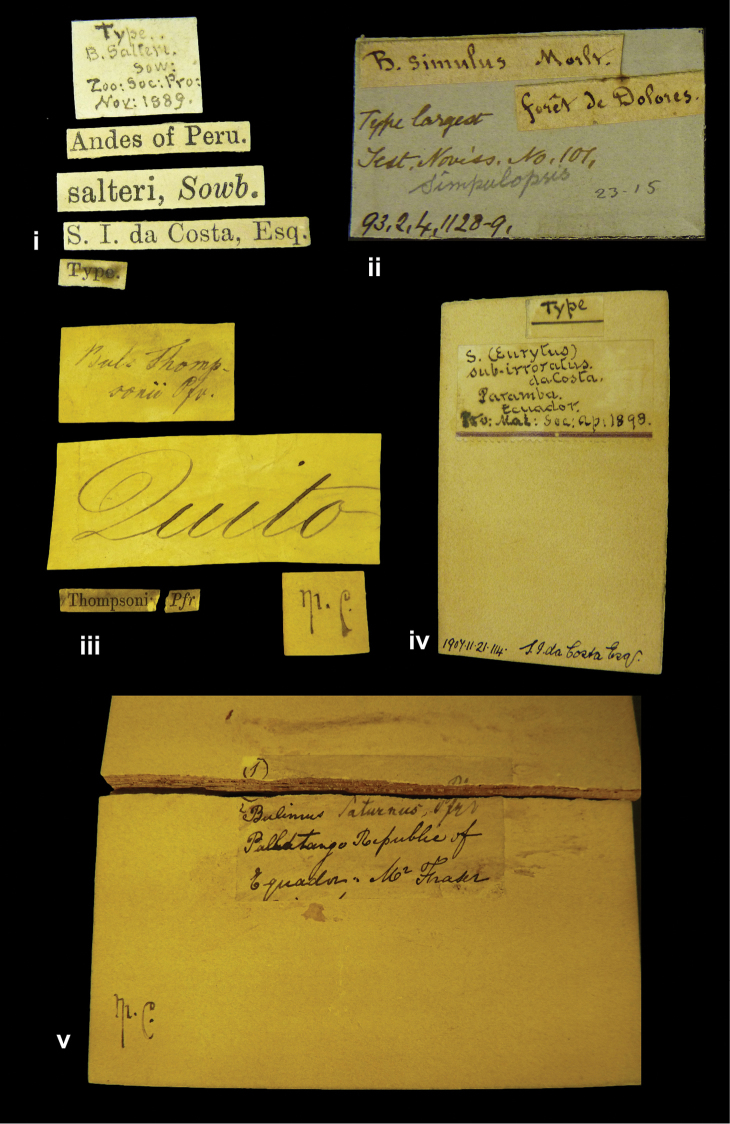
**i** *Bulimus salteri* Sowerby III, 1890 **ii** *Bulimus simulus* Morelet, 1851 **iii** *Bulimus thompsonii* Pfeiffer, 1845 **iv** Strophocheilus (Eurytus) subirroratus da Costa, 1898 **v** *Bulimus saturnus* Pfeiffer, 1860.

**Figure L18.**
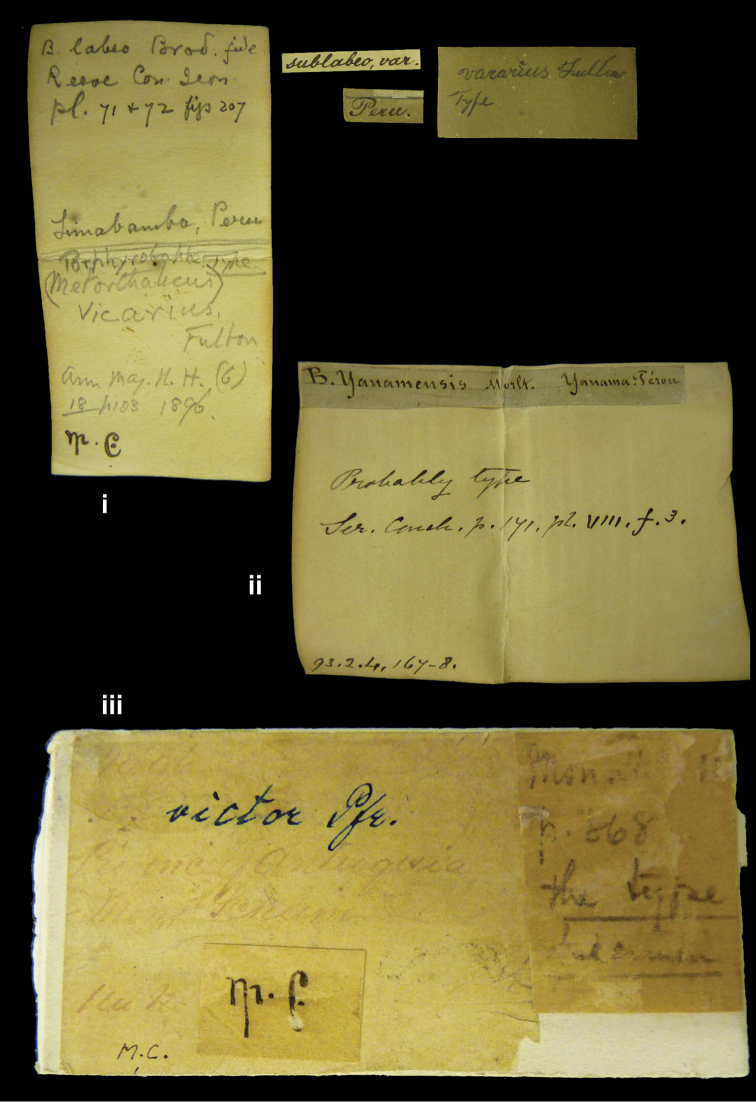
**i** *Porphyrobaphe vicaria* Fulton, 1896 **ii** *Bulimus yanamensis* Morelet, 1863 **iii** *Bulimus victor* Pfeiffer, 1854.

**Figure L19.**
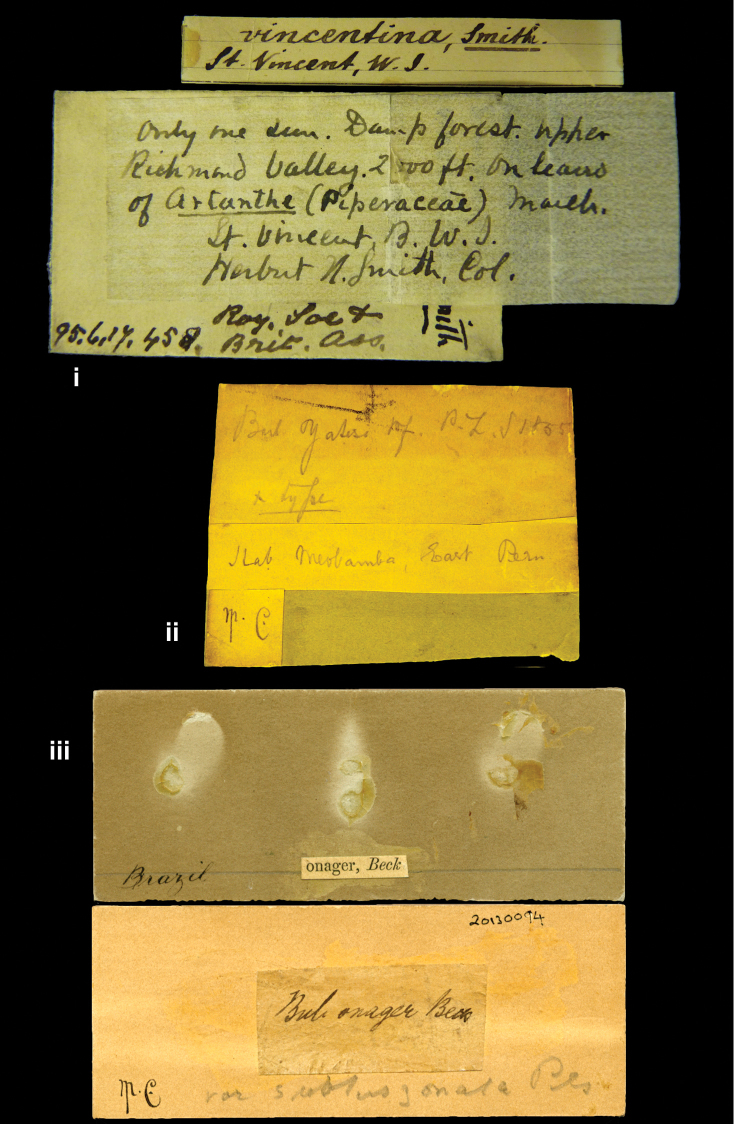
**i** *Simpulosis vincentina* E.A. Smith, 1895 **ii** *Bulimus yatesi* Pfeiffer, 1855 **iii** Drymaeus (Leiostracus) onager var. subtuszonata Pilsbry, 1899.

## Appendix

List of taxa for which Orthalicoidea types are extant, or discussed, in the NHMUK collection

**Remarks.** This list has been compiled from I: Amphibulimidae (Breure and Ablett 2011); II: Bothriembryontidae and Odontostomidae (Breure and Ablett 2012); III: Bulimulidae (Breure and Ablett, 2014); IV: Megaspiridae, Orthalicidae, and Simpulopsidae (this paper). A black star (♦) indicates a *nomen inquirendum*, an asterisk (*) denotes taxa now excluded from the Orthalicoidea, a curved stem sign (¶) is type material not located but previously known to be present; with a dagger (†) taxa are indicated for which type material was expected but not found, and a pilcrow sign (¶) is used for taxa of which material is not (or no longer) considered to be type specimens. Finally, a reference mark (※) indicate the taxa treated otherwise in the text, e.g. as junior or senior synonym.

*abruptus* Rolle, 1904—III, 7

*abscissus* Pfeiffer, 1855—III, 8

*abyssorum* d’Orbigny, 1835—III, 9; ※III, 91

*acalles* Pfeiffer, 1853—III, 9

*acervatus* Pfeiffer, 1857—III, 10

*achatellinus* Forbes, 1850—III, 10

*achilles* Pfeiffer, 1853—IV, 5

*acuminatus* da Costa, 1906—III, 11

*adamsonii* J.E. Gray, 1834†—IV, 41

*adoptus* Reeve, 1849—I, 14

*aenea* Pfeiffer, 1861—IV, 5

*aequatorianus* E.A. Smith, 1877—III, 11; IV, 50

*aequatorius* Pfeiffer, 1853—III, 11

*aestivus* Pfeiffer, 1857—III, 12

*affinis* Broderip in Broderip and Sowerby I 1832—III, 12

*agueroi* Weyrauch, 1960—III, 13

*aileenae* Breure, 1978—III, 13

*alauda* Hupé, 1857—※III, 193

*alba* Crosse, 1874—II, 5

*alba* Sowerby I in J.E. Gray and Sowerby I 1839†—IV, 41

*alabastrinus* da Costa, 1906—III, 14

*albemarlensis* Dall, 1917—III, 14

*albicans* Broderip in Broderip and Sowerby I 1832—III, 15

*albicolor* Morelet, 1863—III, 15

*albolabiatus* E.A. Smith, 1877—III, 16

*albus* Sowerby I, 1833—III, 16

*alexander* Crosse, 1855—※II, 27

*altoperuvianus* Reeve, 1849—III, 17

*alutaceus* Reeve, 1849—IV, 6

*amandus* Pfeiffer, 1855—III, 17; IV, 51

*ambagiosus* Suter, 1906—※II, 32

*ambustus* Reeve, 1849—III, 18; ※III, 41

*andicola* Pfeiffer, 1847—III, 18

*andoicus* Morelet, 1863—III, 18

*angasianus* Pfeiffer, 1864—II, 5

*angrandianus* Pilsbry, 1897♦—※III, 164

*angustus* da Costa, 1906—III, 19

*anthisanensis* Pfeiffer, 1853—III, 19; ※III, 186; IV, 50

*antioquensis* Pfeiffer, 1855—III, 20

*apertus* Pfeiffer, 1855—III, 20

*apicepunctata* Preston, 1914—III, 21

*apiculatus* J.E. Gray, 1834—III, 21

*apodemeta* d’Orbigny, 1835—III, 22; ※III, 148

*appendiculata* Pfeiffer, 1847—I, 15

*approximata* Fulton, 1896—IV, 6

*aquilus* Reeve, 1848—III, 22; IV, 50

*arcuatostriatus* Pfeiffer, 1855—III, 23

*ascendens* Pfeiffer, 1853—IV, 7

*asopeus* Gassies, 1871—II, 6

*atacamensis* Pfeiffer, 1856—III, 23

*atahualpa* Dohrn, 1863—III, 24

*attenuatus* Pfeiffer, 1853—III, 24

*atramentaria* Pfeiffer, 1855†—IV, 41

*aulacostylus* Pfeiffer, 1853†—IV, 41

*aureolus* Guppy, 1866—III, 25

*aurifluus* Pfeiffer, 1857—III, 25

*auriformis* da Costa, 1904—I, 16

*auris* Pfeiffer, 1866—III, 26

*aurissciuri* Guppy, 1866—I, 16

*backhuysi* Delsaerdt, 2010—II, 6

*badius* Sowerby, 1835—III, 26; IV, 50

*bairdii* Reeve, 1848—II, 6

*balsanus* Morelet, 1863—III, 27

*baranguillanus* Pfeiffer, 1853—※III, 20; III, 27

*barbadensis* Pfeiffer, 1853—III, 28

*baroni* (*Helix*) Fulton, 1896—III, 28

*baroni* (*Bulimulus*) Fulton, 1897—III, 29

*bartletti* H. Adams, 1867—III, 29

*bellus* da Costa, 1906—III, 30

*bensoni* Reeve, 1849—IV, 7

*bicolor* Sowerby I, 1835—※III, 27; III, 30; ※III, 155; IV, 50

*bifulguratus* Reeve, 1849—IV, 8

*bilabiatus* Broderip and Sowerby I, 1829†—IV, 41

*bilineatus* Sowerby I, 1833†—IV, 41

*binneyanus* Pfeiffer, 1857—III, 31

*binominis* E.A. Smith, 1895—※III, 106

*bivittatus* Sowerby I, 1833†—※III, 77; IV, 41

*blainvilleanus* Pfeiffer, 1848†—IV, 41

*blandi* Pilsbry, 1897—※III, 30

*bogotensis* Pfeiffer, 1855—III, 31

*bolivarii* d’Orbigny, 1835—III, 32

*bolivianus* Pfeiffer, 1846—III, 32

*bolivianus* Reeve, 1848†—IV, 41

*bonariensis* Rafinesque, 1833—※III, 124

*boucardi* da Costa, 1907—III, 33

*boucardi* Pfeiffer, 1860—IV, 8

*bourcieri* Pfeiffer, 1853—III, 33

*bowkeri* Sowerby III, 1890—II, 7

*brachysoma* d’Orbigny, 1835—III, 34

*brazieri* Angas, 1871—II, 7

*brephoides* d’Orbigny, 1835—IV, 9

*broadwayi* E.A. Smith, 1896—III, 34

*broderipii* Sowerby I, 1832—II, 8; ※II, 16; ※II, 17; ※II, 35; ※II, 43

*bruggeni* Breure, 1978—I, 17

*brunneum* Verdcourt, 1991—II, 8

*buckleyi* Higgins, 1872—IV, 9

*buckleyi* Sowerby III, 1895—III, 35

*bugabensis* Martens, 1893—III, 35

*bulbulus* Gassies, 1871—II, 9

*cacticolus* Reeve, 1849—III, 36

*cactivorus* Broderip in Broderip and Sowerby I 1832†—※III, 133; IV, 41

*cactorum* d’Orbigny, 1835—III, 36

*caledonicus* Petit de la Saussaye, 1845—※II, 16

*californicus* Reeve, 1848—III, 37

*caliginosus* Reeve, 1849—III, 37; IV, 50

*calus* E.A. Smith, 1891—II, 9

*canaliculatus* Pfeiffer, 1845—III, 37

*cancellata* da Costa, 1906—III, 38

*cantatus* Reeve, 1848†—IV, 41

*cardinalis* Pfeiffer, 1853†—IV, 41

*carinatum* Pfeiffer, 1853—II, 10

*castaneostrigatus* da Costa, 1906—III, 38

*castaneus* Pfeiffer, 1845—I, 17

*castelnaui* Pfeiffer, 1857†—IV, 41

*castrensis* Pfeiffer, 1847†—IV, 41

*castus* Pfeiffer, 1847—III, 39

*catharinae* Pfeiffer, 1857—II, 10

*cathcartiae* Reeve, 1848—I, 17

*catlowiae* Pfeiffer, 1853—III, 39

*caucaensis* da Costa, 1898—III, 40

*ceratacme* Pfeiffer, 1855—III, 40

*cercicola* Morelet, 1863—III, 40

*chacoensis* Preston, 1907†—IV, 41

*chamaeleon* Pfeiffer, 1855—III, 41

*chamayensis* Weyrauch, 1967—III, 41

*championi* Martens, 1893—III, 42

*charpentieri* Pfeiffer, 1850—II, 10

*chemnitzioides* Forbes, 1850—III, 42

*chiletensis* Weyrauch, 1967—III, 43

*chimborasensis* Reeve, 1848—III, 43

*chiriquensis* da Costa, 1901—III, 43

*chrysostoma* Moricand, 1836—※III, 190

*cinereus* Reeve, 1849—III, 44

*citrinovitrea* Moricand, 1836—※IV, 24

*citronellus* Angas, 1879—III, 44

*clarus* Pfeiffer, 1857—III, 45

*clathratus* Pfeiffer, 1858—III, 45

*clouei* Pfeiffer, 1857—IV, 10

*coagulatus* Reeve, 1849—III, 46; IV, 50

*coarctatus* Pfeiffer, 1845—III, 46

*coerulescens* Pfeiffer, 1858†—IV, 41

*coloratus* Nyst, 1845※—I, 23

*columbianus* Lea, 1838—※III, 84; ※III, 86

*columbiensis* Pfeiffer, 1855—III, 47

*columellaris* Reeve, 1849†—IV, 41

*compactus* Fulton, 1902—III, 47

*confinus* Reeve, 1850†—IV, 41

*confluens* Pfeiffer, 1855—III, 48

*confusus* Reeve, 1848—III, 48

*conicus* da Costa, 1907—III, 49; IV, 51

*coniformis* Pfeiffer, 1847†—IV, 41

*consimilis* Reeve, 1848—IV, 10

*conspersus* Sowerby I, 1833—III, 49

*constrictus* Pfeiffer, 1841—※IV, 18

*constrictus* Reeve, 1848†—IV, #42

*contortuplicatus* Reeve, 1850†—IV, 42

*convexus* Pfeiffer, 1855—III, 50; ※III, 153

*coquimbensis* Broderip in Broderip and Sowerby I 1832†—IV, 42

*cora* d’Orbigny, 1835—III, 50

*corderoi* Klappenbach, 1958—II, 11

*coriaceus* Pfeiffer, 1857—III, 51

*corneus* Sowerby I, 1833†—※III, 135; IV, 42

*corpulentus* Gassies, 1871—II, 11

*corrugatus* Guppy, 1866—IV, 10

*corrugatus* King in King and Broderip 1831†—IV, 42

*corticosus* Sowerby III, 1895—I, 18

*costatus* Pfeiffer, 1848—II, 12

*costatus* Weyrauch, 1960—III, 51

*costifer* Weyrauch, 1960—III, 51

*cotopaxiensis* Pfeiffer, 1853—III, 52; IV, 50

*coturnix* Sowerby I, 1832—II, 12

*crassilabrum* Garrett, 1872—II, 12

*crepundia* d’Orbigny, 1835—III, 52

*cretaceus* Pfeiffer, 1855—※III, 29; III, 53

*crichtoni* Broderip, 1836—III, 54; ※III, 93

*cucullus* Morelet, 1849*—IV, 51

*culminea* d’Orbigny, 1835—※III, 49; III, 54; ※III, 101; ※III, 111

*cumingi* Pfeiffer, 1861—IV, 11

*cumingii* ‘Newcomb’ Pfeiffer, 1849—II, 13

*cuninculinsulae* Cox, 1872—II, 13

*curianianus* Reeve, 1849†—IV, 42

*curtus* Reibisch, 1892—III, 55

*cuticula* Pfeiffer, 1855—III, 55

*cuzcoensis* Reeve, 1849—III, 56

*cylindricus* Fulton, 1907—II, 14

*dacostae* Sowerby III, 1892—III, 56

*darwini* Pfeiffer, 1846—III, 56

*dealbatus* Say, 1821—※III, 110; ※III, 165

*deburghiae* Reeve, 1859—IV, 12; ※IV, 16

*decoloratus* Sowerby I, 1833†—IV, 42

*decussata* Pfeiffer, 1856—IV, 12

*decussatus* Reeve, 1849—III, 57; ※III, 130

*dejectus* Fulton, 1907♦—IV, 38

*delumbis* Reeve, 1849—III, 57

*demerarensis* Pfeiffer, 1861—IV, 13

*demotus* Reeve, 1850—III, 58

*denickei* J.E. Gray, 1852—III, 58

*dennisoni* Reeve, 1848¶—IV, 39

*dentata* Wood, 1828—II, 14

*dentritis* Morelet, 1863—III, 59

*depictus* Reeve, 1849—III, 59

*depstus* Reeve, 1849—III, 60

*derelictus* Broderip, 1832—III, 60

*deshayesi* Pfeiffer, 1845—III, 61

*devians* Dohrn, 1863—III, 61

*diaphanus* Pfeiffer, 1855¶—IV, 45

*dillwynianus* Pfeiffer, 1853—I, 18

*discrepans* Sowerby I, 1833—III, 62

*dissimulans* Preston, 1909—I, 19

*doliarius* da Costa, 1898—I, 19

*dombeyanus* ‘Férussac’ Pfeiffer, 1846—III, 62

*draparnaudi* Pfeiffer, 1847†—IV, 42

*droueti* Pfeiffer, 1857†—IV, 42

*dubius* Pfeiffer, 1853—III, 62

*dukinfieldi* Melvill, 1900♦—III, 63

*dunkeri* Pfeiffer, 1846—III, 64

*durangoanus* Martens, 1893—III, 64

*dutaillyi* Pfeiffer, 1857—III, 65

*dux* Pfeiffer, 1861—II, 15

*dysoni* Pfeiffer, 1846—III, 65

*eddystonensis* Pfeiffer, 1855—II, 15

*edwardsi* Morelet, 1863—III, 65

*edwardsianus* Gassies, 1863—II, 16

*effeminatus* Reeve, 1848—III, 66

*eganus* Pfeiffer, 1853†—IV, 42

*elaeodes* Pfeiffer, 1853—I, 46

*elata* Gould, 1847—IV, 13

*electrum* Reeve, 1848—III, 66

*elegans* Pfeiffer, 1842—II, 16

*elongata* d’Orbigny, 1837—II, 17

*elongatus* Röding, 1789—※III, 22

*elsteri* da Costa, 1901—III, 67

*emaciatus* Morelet, 1863—III, 67

*emeus* Say, 1830—※III, 92

*ephippium* Ancey, 1904—IV, 14

*episcopalis* Pfeiffer, 1855—I, 20

*erectus* Reeve, 1849—III, 68

*eros* Angas, 1878—I, 20

*erosus* Broderip in Broderip and Sowerby I 1832—III, 68

*erubescens* Pfeiffer, 1847—III, 69

*erythrostoma* Sowerby I, 1833†—IV, 42

*eschariferus* Sowerby I, 1838—III, 69; IV, 51

*euryomphalus* Jonas, 1844※—I, 27

*excoriatus* Pfeiffer, 1855—III, 70

*exornatus* Reeve, 1849—III, 70

*exoticus* da Costa, 1901—III, 70

*expansus* Pfeiffer, 1848—※III, 177

*expatriatus* Preston, 1909—III, 71

*fabrefactus* Reeve, 1848—III, 71

*falcicula* Gassies, 1871—II, 17

*fallax* Pfeiffer, 1853—III, 72

*farrisi* Pfeiffer, 1858—III, 72

*felix* Pfeiffer, 1862—III, 73; IV, 51

*fenestratus* Pfeiffer, 1846—III, 73

*fenestrellus* Martens, 1864—※III, 82

*feriatus* Reeve, 1848—III, 74

*fernandezae* Weyrauch, 1958—III, 74

*ferrugineus* Reeve, 1849—III, 75

*fibratus* Martyn, 1784—※II, 6; ※II, 7; ※II, 8; ※II, 17; ※II, 30; ※II,41

*fidustus* Reeve, 1849—III, 75

*filaris* Pfeiffer, 1853—III, 76; IV, 50

*flavescens* King in King and Broderip 1831†—IV, 42

*flavidulus* E.A. Smith, 1877—III, 76

*flexilabris* Pfeiffer, 1853—III, 76

*flexuosus* Pfeiffer, 1853—III, 77

*floridanus* Pfeiffer, 1857—III, 77

*fontainii* d’Orbigny, 1838—III, 78

*fourmiersi* d’Orbigny, 1837—III, 78

*foveolatus* Pfeiffer, 1848—IV, 14

*foxi* Clench, 1950—II, 18

*fraseri* Pfeiffer, 1858—IV, 15

*fucatus* Reeve, 1849—III, 79

*fuligineus* Pfeiffer, 1853—II, 18

*funckii* Nyst, 1843—※I, 14

*fuscagula* d’Orbigny, 1837—II, 19

*fuscobasis* E.A. Smith, 1877—III, 79

*fuscus* Guilding, 1828—※III, 28

*fusiformis* Menke, 1828—※II, 10

*fusoides* d’Orbigny, 1835—III, 80

*gabbi* Angas, 1879—III, 80

*galapaganus* Pfeiffer, 1855—III, 81

*gargantua* Férussac, 1821—※II, 30

*gatopensis* Crosse, 1870—II, 19

*gayi* Pfeiffer, 1857—III, 81; ※III, 193

*gealei* H. Adams, 1867—III, 82

*gelidus* Reeve, 1849♦—III, 82

*geometricus* Pfeiffer, 1846—III, 83

*glandiniformis* Sowerby III, 1892*—IV, 51

*glomeratus* Weyrauch, 1960—III, 83

*gloriosus* Pfeiffer, 1862—IV, 15

*gomesae* da Silva & Thomé, 2006—IV, 16

*goroensis* Souverbie, 1870—※II, 20

*gracilis* E.A. Smith, 1902—IV, 16

*grammica* Crosse, 1870—II, 20

*granadensis* Pfeiffer, 1848—※III, 58; ※III, 93; ※III, 112

*grandiventris* Weyrauch, 1960—III, 84

*granulosus* Broderip in Broderip and Sowerby I 1832†—IV, 42

*gravesii* King in King and Broderip 1831†—IV, 42

*grayanus* Pfeiffer, 1845—II, 20

*grenadensis* Guppy, 1868†—IV, 42

*gruneri* Pfeiffer, 1846—III, 84

*guadalupensis* Bruguière, 1789—※III, 171

*guarani* d’Orbigny, 1835—II, 20

*gueinzii* Pfeiffer, 1857—III, 85

*guentheri* Sowerby III, 1892†—IV, 42

*guerini* Pfeiffer, 1846—I, 21

*guestieri* Gassies, 1869—※II, 19; ※II, 22; ※II, 32

*guppyi* E.A. Smith, 1891—II, 21

*guttatus* Broderip, 1832—III, 85

*guttula* Pfeiffer, 1854†—IV, 42

*haasi* Weyrauch, 1960—※III, 121

*habeli* Dall, 1892—※III, 191

*hachensis* Reeve, 1850—III, 86

*hamiltoni* Reeve, 1849—III, 86

*haplochrous* Pfeiffer, 1855—III, 87

*hargravesi* Cox, 1871—II, 21

*hartwegi* Pfeiffer, 1846—IV, 17

*hegewischi* Pfeiffer, 1842†—IV, 42

*heloica* d’Orbigny, 1835—III, 87

*hennahi* J.E. Gray, 1828†—※III, 36; IV, 42

*hepatostomus* Pfeiffer, 1861—III, 88

*hidalgoi* da Costa, 1898—III, 88

*hoffmanni* Martens, 1893—※III, 38

*holostoma* Pfeiffer, 1846—III, 89

*hondurasanus* Pfeiffer, 1846—III, 89

*huascensis* Reeve, 1848—III, 90

*humboldtii* Reeve, 1849—III, 90

*hyaloideus* Pfeiffer, 1855—IV, 17

*hyematus* Reeve, 1848†—IV, 42

*hygrohylaea* d’Orbigny, 1835—III, 91

*hypozonus* Martens, 1893—III, 91

*ictericus* Martens, 1893†—IV, 42

*ignavus* Reeve, 1849—III, 92

*illustris* Rolle, 1904—III, 92

*imeldae* Weyrauch, 1958—※III, 54

*immaculatus* C.B. Adams in Reeve, 1850—III, 93

*imperfectus* Guppy, 1866—※III, 197

*inca* d’Orbigny, 1835—IV, 18

*inaequalis* Pfeiffer, 1857†—IV, 42

*incarnatus* Pfeiffer, 1855—III, 93

*inclinatus* Pfeiffer, 1862—III, 94

*incognita* da Costa, 1907—III, 94

*incrassatus* Pfeiffer, 1853—III, 94

*indentatus* da Costa, 1901—IV, 18

*inermis* Morelet, 1851*—III, 214

*inflatus* Broderip, 1836†—IV, 42

*infundibulum* Gassies, 1871—II, 22

*infundibulum* Pfeiffer, 1853—III, 95

*inglorius* Reeve, 1848—III, 95

*insolitus* Preston, 1909—IV, 19

*integer* Pfeiffer, 1855—IV, 19

*interruptus* Preston, 1909—III, 96; ※IV, 42

*inusitatus* Fulton, 1900—III, 96

*inutilis* Reeve, 1850—III, 97

*iodostylus* Pfeiffer, 1861—III, 97

*iostoma* Sowerby I, 1824†—IV, 43

*iris* Pfeiffer, 1853—IV, 20

*irregularis* Pfeiffer, 1848—III, 98

*irroratus* Reeve, 1849—IV, 20

*ischnus* Pilsbry, 1902—※III, 192

*istapensis* Crosse & Fischer, 1873—III, 98

*jacobi* Sowerby I, 1833—III, 99; ※III, 142

*janeirensis* Sowerby I, 1833†—IV, 43

*jansoni* Martens, 1893—III, 99

*jeffreysi* Pfeiffer, 1852—IV, 21

*jonasi* Pfeiffer, 1846—III, 100

*josephus* Angas, 1878—III, 100

*juarezi* Pfeiffer, 1866—III, 101

*jubeus* Fulton, 1908—I, 21

*jucundus* Pfeiffer, 1855†—IV, 43

*jussieui* Pfeiffer, 1846—III, 101; IV, 51

*juvenilis* Pfeiffer, 1855—III, 102

*kathiae* Breure, 1978—III, 102

*kelletti* Reeve, 1850—IV, 21

*keppelli* Pfeiffer, 1853—III, 102

*kingii* J.E. Gray, 1825—II, 22

*knorri* Pfeiffer in Philippi, 1846—III, 103

*koppeli* Sowerby III, 1892—III, 103

*koroensis* Garrett, 1872—II, 23

*kreftii* Cox, 1872—II, 23

*labeo* Broderip, 1828†—IV, 43

*labeo* Reeve, 1848—※IV, 35

*lacerta* Pfeiffer, 1855—I, 22

*lactifluus* Pfeiffer, 1857—III, 104

*laetus* Reeve, 1849—III, 104

*lamarckianus* Pfeiffer, 1848—I, 23

*lamas* Higgins, 1868—III, 105

*largillierti* Philippi, 1842—※IV, 10

*lascellianus* E.A. Smith, 1895—III, 105

*latecolumellaris* Preston, 1909—III, 106

*latilabris* Pfeiffer, 1855—I, 23

*lattrei* Pfeiffer in Philippi, 1846—III, 106

*laurentii* Sowerby I, 1833†—IV, 43

*laxostylus* Rolle, 1904—III, 107

*leeuwinensis* E.A. Smith, 1894—II, 24

*lesueureanus* Morelet, 1860—III, 107

*lichnorum* d’Orbigny, 1835—III, 108

*lilacinus* Reeve, 1849—III, 108; ※III, 144

*limensis* Reeve, 1849—III, 109

*limonoica* d’Orbigny, 1835—III, 109

*lindeni* Reeve, 1848†—IV, 43

*linostoma* d’Orbigny, 1835—III, 110

*linterae* Sowerby III, 1890—I, 24

*liquabilis* Reeve, 1848—III, 110

*lirinus* Morelet, 1851—III, 111

*listeri* Wood, 1828†—IV, 43

*lithoica* d’Orbigny, 1835—III, 111

*lividus* Reeve, 1850—III, 112

*lobbii* Reeve, 1849—III, 112; ※III, 159

*longinquus* Morelet, 1863—III, 112

*longulus* ‘Behn’ Pfeiffer, 1859—II, 24

*lophoica* d’Orbigny, 1835—III, 113

*loveni* Pfeiffer, 1848—I, 24

*loxanus* Higgins, 1872—¶III, 113

*loxensis* Pfeiffer, 1846—III, 114

*loxostomus* Pfeiffer, 1853—IV, 22

*lucidus* da Costa, 1898—III, 114

*lucidus* Reeve, 1848—III, 115

*luridus* Pfeiffer, 1863—III, 115

*lusorius* Pfeiffer, 1855—III, 116

*lycodus* Dall, 1917—III, 116

*macandrewi* Sowerby III, 1889†—IV, 43

*macgillivrayi* Pfeiffer, 1855—II, 25

*magnifica* Pfeiffer, 1848—IV, 22

*magnificus* Grateloup, 1839—IV, 23

*mahogany* Pfeiffer, 1841—※IV, 14

*major* d’Orbigny, 1837—II, 25; IV, 45

*malleatus* da Costa, 1898—III, 117

*manupictus* Reeve, 1848—III, 117

*marcidus* Pfeiffer, 1853—III, 117

*marmarina* d’Orbigny, 1835—III, 118

*marmatensis* Pfeiffer, 1855—IV, 24

*marmoratus* Dunker in Philippi, 1844—I, 25

*martinicensis* Pfeiffer, 1846—III, 118

*mars* Pfeiffer, 1861—IV, 24

*mejillonensis* Pfeiffer, 1857—III, 119

*meleagris* Pfeiffer, 1853—III, 119

*melo* Quoy and Gaimard, 1832—※II, 31

*meobambensis* Pfeiffer, 1855—IV, 25

*meridanus* Pfeiffer, 1846—III, 120

*metagyra* Pilsbry & Olsson, 1949—III, 120

*mexicanus* Lamarck, 1822—※III, 90

*miersi* Pfeiffer, 1856—IV, 25

*miliola* d’Orbigny, 1835¶—IV, 39

*miltocheilus* Reeve, 1848—II, 26

*minor* d’Orbigny, 1837—II, 26

*minor* Weyrauch, 1960—III, 121

*modestus* Broderip in Broderip and Sowerby I 1832—※III, 109; III, 121; ※III, 150

*mollicellus* Reeve, 1849—III, 121

*monachus* Pfeiffer, 1857—III, 122

*moniezi* Dautzenberg, 1896—III, 122

*monilifer* Reeve, 1848—III, 123

*montagnei* d’Orbigny, 1837—III, 123

*montevidensis* Pfeiffer, 1846—III, 124

*montivaga* d’Orbigny, 1835—III, 124

*mordani* Breure, 1978—III, 125

*moricandi* Pfeiffer, 1847—III, 125

*moritinctus* Martens, 1893—III, 126

*mossi* E.A. Smith, 1896—III, 126

*moussoni* Pfeiffer, 1853—III, 127

*muliebris* Reeve, 1849—III, 127

*multilineatus* Say, 1825—※III, 180

*multispira* da Costa, 1904—III, 128

*munsterii* d’Orbigny, 1837—III, 128

*murrea* Reeve, 1849—IV, 26

*murrinus* Reeve, 1848—III, 129; ※III, 151

*musivus* Pfeiffer, 1855—III, 129

*mutabilis* Broderip in Broderip and Sowerby I 1832†—IV, 43

*myristicus* Reeve, 1849—III, 130

*nanus* Reeve, 1849—III, 130

*napo* Angas, 1878—III, 130

*navarrensis* Angas, 1878†—IV, 43

*nebulosus* Martens, 1893†—IV, 43

*necouensis* Gassies, 1871—II, 27

*neglectus* Pfeiffer, 1847—II, 27; ※II, 28

*nigrofasciatus* Pfeiffer in Philippi, 1846—III, 131

*nigrolimbatus* Pfeiffer, 1853—III, 132

*nigropileatus* Reeve, 1849—※III, 27; III, 132; ※III, 166

*nigroumbilicatus* Preston, 1907†—IV, 43

*nitelinus* Reeve, 1849—III, 132

*nitidus* Broderip in Broderip and Sowerby I 1832—III, 133

*nivalis* d’Orbigny, 1835♦—III, 134

*niveus* Preston, 1909—IV, 26

*notabilis* da Costa, 1906—III, 134

*notatus* da Costa, 1906—III, 134

*nubeculatus* Pfeiffer, 1853—III, 135

*nubilus* Preston, 1903—III, 135

*nucinus* Reeve, 1850—III, 136; IV, 50

*nucula* Pfeiffer, 1853—III, 136

*nuptialis* Melvill & Ponsonby, 1894—II, 28

*nux* Broderip, 1832—※III, 95; III, 137; ※III, 204

*nystianus* Pfeiffer, 1853—III, 137

*obliquistriatus* da Costa, 1901—III, 138

*obliquus* Reeve, 1849—※IV, 21; IV, 26

*oblitus* Reeve, 1848—II, 28

*occultus* Reeve, 1849—II, 28; ※II, 31

*ochraceus* Morelet, 1863—III, 138; IV, 50

*ochrocheilus* E.A. Smith, 1877—III, 139

*ochrostoma* Garrett, 1872—II, 29

*odontostoma* Sowerby I, 1824—II, 29

*onager* Reeve, 1848—※IV, 34

*onca* d’Orbigny, 1835—I, 25

*opalinus* Sowerby I, 1833—IV, 27

*orbignyi* Pfeiffer, 1846—※III, 21; III, 139

*oreades* d’Orbigny, 1835—III, 140

*orobaena* d’Orbigny, 1835¶—IV, 43

*orophilus* Morelet, 1860—※III, 15; ※III, 41; ※III, 108; III, 140

*orthostoma* E.A. Smith, 1877—III, 141

*otostomus* Pfeiffer, 1855—I, 27

*ouensis* Gassies, 1870—II, 30, IV, 45

*ouveanus* Dotzauer in Mousson, 1869—※II, 5; ※II, 39

*ovulum* Reeve, 1849—IV, 27

*pallens* Reeve, 1849♦—III, 141

*pallidior* Sowerby I, 1833†—IV, 43

*pallidus* Preston, 1909—IV, 46

*pallidus* Reibisch, 1892—III, 142

*panamensis* Broderip, 1832—III, 142

*papillatus* Morelet, 1860—III, 143

*paposensis* Pfeiffer, 1856—III, 143

*parallelus* Pfeiffer, 1857—II, 30

*pardalina* Guppy, 1868—I, 27

*pardalis* Reeve, 1848†—IV, 43

*patagonica* d’Orbigny, 1835—II, 31

*patasensis* Pfeiffer, 1858—III, 144

*patricius* Reeve, 1849—III, 144

*paucicostatus* Breure, 1978—III, 144

*paziana* d’Orbigny, 1835—III, 145

*peaki* Breure, 1978—III, 145; IV, 50

*peelii* Reeve, 1859†—IV, 43

*pentadinus* d’Orbigny, 1835—※I, 26

*pentlandi* Reeve 1849†—IV, 43

*perdix* Pfeiffer, 1848—I, 27

*perenensis* da Costa, 1901—III, 146

*pergracilis* Rolle, 1904—III, 146

*perlucidus* Spix, 1827—※IV, 27

*perspectivus* Pfeiffer, 1846—III, 147

*pertristis* Pfeiffer, 1855—※III, 197

*pervariabilis* Pfeiffer, 1853—III, 147

*pervius* Pfeiffer, 1853—III, 148

*pessulatus* Reeve, 1848—III, 148

*petenensis* Morelet, 1851—III, 149

*petiti* Pfeiffer, 1846—III, 149

*philippii* Pfeiffer, 1842—III, 150

*phlegonis* Dall & Ochsner, 1928—III, 150

*phlogera* d’Orbigny, 1835—IV, 28

*phoebus* Pfeiffer, 1863—IV, 28

*phryne* Pfeiffer, 1863—III, 151

*physoides* Reeve, 1849—II, 31

*pictus* Pfeiffer, 1855—III, 151

*pilosus* Guppy, 1871—III, 151

*pinicola* Gassies, 1870—II, 32

*piperitus* Sowerby I, 1837—I, 28

*pittieri* Martens, 1893†—IV, 43

*platystomus* Pfeiffer, 1858—III, 152

*plectostylus* Pfeiffer, 1848—I, 30

*plicatoliratus* da Costa, 1898—III, 152

*pliculatus* Pfeiffer, 1857—III, 153

*plumbeus* Pfeiffer, 1855—IV, 29

*poecila* d’Orbigny, 1835—III, 153

*polymorpha* d’Orbigny, 1835—III, 154; IV, 50

*ponsonbyi* da Costa, 1907—III, 155

*porphyrius* Pfeiffer, 1847—IV, 30

*porphyrostomus* Pfeiffer, 1851—※II, 39

*powisiana* Petit de la Saussaye, 1843—※IV, 43

*praetextus* Reeve, 1849—III, 155

*prestoni* da Costa, 1906—III, 156

*primula* Reeve, 1848—III, 156

*primularis* Reeve, 1849†—IV, 43

*princeps* Breoderip in Sowerby I 1833†—IV, 43

*principalis* Sowerby II, 1849†—IV, 43

*priscus* Powell, 1938—II, 32

*progastor* d’Orbigny, 1835¶—IV, 40

*proteus* Broderip, 1832—III, 157

*protractus* Pfeiffer, 1855—III, 157

*pruinosus* Sowerby I, 1833—III, 158

*pseudofusoides* da Costa, 1906—III, 158

*ptychostylus* Pfeiffer, 1858—III, 159

*puellaris* Reeve, 1850—III, 159

*pulchellus* Broderip in Broderip and Sowerby I 1832†—IV, 43

*pulchellus* Sowerby I, 1833†—IV, 43

*pulcherrimus* H. Adams, 1867—III, 160

*pulicarius* Reeve, 1848—I, 31

*pumilio* Rehder, 1945—※III, 130

*punctatus* da Costa, 1907—III, 160

*punctulifer* Sowerby I, 1833—II, 33

*pupiformis* Broderip, 1832—III, 161

*purpuratus* Reeve, 1849—III, 161; IV, 50

*pustulosus* Broderip, 1832—III, 162

*pygmeus* Weyrauch, 1960—※III, 51

*pyrostomus* Pfeiffer, 1860—II, 33

*quadingensis* Connolly, 1929—II, 34

*quadrifasciatus* Angas, 1878—※III, 131; III, 162

*quadricolor* Pfeiffer, 1848—I, 31

*quechuarum* Crawford, 1939—III, 163; IV, 50

*quitensis* Pfeiffer, 1848—※III, 39; ※III, 98; III, 163

*radiatus* Morelet, 1863—III, 164

*ragsdalei* Pilsbry, 1890—III, 164

*ramagei* E.A. Smith, 1890—II, 34

*rawsonis* H. Adams, 1873—III, 165

*recedens* Pfeiffer, 1864—III, 165

*recluzianus* Pfeiffer, 1847—※III, 135

*reconditus* Reeve, 1849—III, 166

*rectilinearis* Pfeiffer, 1855—III, 166

*reflexa* Pfeiffer, 1842—II, 34

*regularis* Fulton, 1905—III, 167

*rehderi* Weyrauch, 1960—III, 167

*requieni* Pfeiffer, 1853—IV, 30

*revinctus* Hupé, 1857—※III, 193

*rhodacme* Pfeiffer, 1842†—IV, 43

*rhodinostoma* d’Orbigny, 1835—II, 35

*rhodocheilus* Reeve, 1848—I, 34

*rhodolarynx* Reeve, 1849—III, 167

*rhodostomus* J.E. Gray, 1834—II, 35

*ridleyi* E.A. Smith, 1890—II, 36

*rimatus* Pfeiffer, 1847—III, 168

*rivasii* d’Orbigny, 1837—III, 168; ※III, 188

*rocayana* d’Orbigny, 1835—III, 169

*rodriguezae* Weyrauch, 1967—III, 169

*roseatus* Reeve, 1848—III, 170

*rosenbergi* da Costa, 1906—III, 170

*roseolabrum* E.A. Smith, 1877—I, 36

*rubellus* Broderip, 1832†—IV, 44

*rubescens* Reeve, 1848†—IV, 44

*rubrifasciatus* Reeve, 1848—III, 171

*rubrovariegatus* Higgins, 1868—III, 171

*rufescens* J.E. Gray, 1825—※III, 69

*rufovirens* Moricand, 1846—※IV, 31

*rugiferus* Sowerby I, 1833—III, 171

*rugulosus* Sowerby I, 1833—III, 172

*rupicolus* Reeve, 1848†—IV, 44

*rusticellus* Morelet, 1860—III, 172

*saccatus* Pfeiffer, 1855—III, 173

*salomonia* Pfeiffer, 1853—IV, 31

*salomonis* Pfeiffer, 1853—※II, 33; II, 36; IV, 45

*salteri* Sowerby III, 1890—IV, 31

*sanchristovalensis* Cox, 1870—II, 37

*sanctaeluciae* E.A. Smith, 1889—III, 173

*sarcochilus* Pfeiffer, 1857—IV, 32

*sarcodes* Pfeiffer, 1846†—IV, 44

*saturnus* Pfeiffer, 1860—IV, 33

*savesi* Crosse, 1886—II, 37

*sayi* Pfeiffer, 1847†—IV, 44

*scabiosus* Sowerby I, 1833—III, 174

*scalaricosta* Morelet, 1860—III, 174

*scalariformis* Broderip, 1832—III, 175

*schiedeanus* Pfeiffer, 1841—III, 175

*schmidti* Pfeiffer, 1854—III, 176

*scytodes* Pfeiffer, 1853†—IV, 44

*scitulus* Reeve, 1849—III, 176

*scitus* H. Adams, 1867—III, 176

*sculpturatus* Pfeiffer, 1846—III, 177

*scytodes* Pfeiffer, 1853—I, 37

*sellersi* Cox, 1872—II, 38

*selli* Preston, 1909—III, 177

*senilis* Gassies, 1869—IV, 44

*serotinus* Morelet, 1860—III, 178

*serparastrus* Say, 1830—※III, 133

*serratus* Pfeiffer, 1855—III, 178

*signifer* Pfeiffer, 1855—III, 179

*simpliculus* Pfeiffer, 1855—III, 179

*simulus* Morelet, 1851—IV, 33

*singularis* Morelet, 1857—II, 38

*sinistrorsa* Crosse, 1884—II, 39

*sisalensis* Morelet, 1849—III, 180; IV, 51

*smithii* da Costa, 1898—III, 180

*solidus* Preston, 1907—III, 181

*souvillei* Morelet, 1857—II, 39

*sowerbyi* Pfeiffer, 1847♦—III, 181

*spadiceus* da Costa, 1906—III, 182

*speciosus* Pfeiffer, 1854—I, 38

*spectatus* Reeve, 1849—III, 182

*spenceri* Tate, 1894—II, 40

*spiculatus* Morelet, 1860—III, 183

*sporadica* d’Orbigny, 1835—III, 183

*sporadicus* Reeve, 1848†—IV, 44

*stenacme* Pfeiffer, 1857—III, 184

*stramineus* Guilding, 1824—※III, 115

*strangei* Pfeiffer, 1855—II, 40

*striata* Spix, 1827—※II, 26

*striatulus* Sowerby I, 1833†—IV, 44

*striatus* ‘King’ Sowerby I, 1833†—IV, 44

*strigatus* Sowerby I, 1833—※III, 129; ※III, 173; III, 185

*studeri* Pfeiffer, 1847—※III, 156; III, 185

*subfasciatus* Pfeiffer, 1853—III, 186; IV, 50

*subhybridus* da Costa, 1906—III, 186

*subinterruptus* Pfeiffer, 1853—III, 187

*subirroratus* da Costa, 1898—IV, 34

*subpellucidus* E.A. Smith, 1877—III, 187

*subroseus* Fulton, 1915—II, 41

*subtuszonatus* Pilsbry, 1899—IV, 34

*subventricosus* da Costa, 1901—III, 188

*succineoides* Petit de la Saussaye, 1840—※I, 24

*sufflatus* Gould, 1859—※III, 101

*sugillatus* Pfeiffer, 1857—III, 188

*sulcosus* Pfeiffer, 1841—III, 188

*sulphureus* Pfeiffer, 1857—※III, 45; III, 189

*superfasciatus* Gassies, 1871—II, 41

*superstriatus* Sowerby III, 1890—I, 40

*swainsoni* Pfeiffer, 1845—III, 189

*sykesi* da Costa, 1906—III, 190

*taquinensis* Pfeiffer, 1855—I, 41

*tasmanicus* Pfeiffer, 1853—II, 41

*taunaisii* Férussac, 1822—※IV, 5

*taylori* Pfeiffer, 1861†—IV, 44

*taylorianus* Reeve, 1849—I, 42

*tenuilabris* Pfeiffer, 1866—III, 190

*terebra* Reibisch, 1892—III, 191

*terebralis* Pfeiffer, 1842—III, 191

*thamnoica* d’Orbigny, 1835—III, 192

*thompsonii* Pfeiffer, 1845—IV, 35

*tigrinus* da Costa, 1898—III, 193

*tigris* Broderip in Broderip and Sowerby I 1832†—IV, 44

*toleratus* Fulton, 1903—II, 42

*torallyi* d’Orbigny, 1835—※III, 128; III, 194

*translucens* Broderip in Broderip and Sowerby I 1832—III, 194

*transparens* Reeve, 1849—III, 195

*trichoda* d’Orbigny, 1835—III, 195

*tricinctus* Reeve, 1848—III, 196

*trigonostomus* Jonas, 1844—※III, 103

*trimarianus* Martens, 1893—III, 196

*trinitarius* E.A. Smith, 1896—III, 197

*tristis* Pfeiffer, 1855—III, 197

*tropicalis* Morelet, 1849—III, 198

*trujillensis* Philippi, 1867—※III, 105

*tumidulus* Pfeiffer, 1842—III, 198

*tupacii* d’Orbigny, 1835—III, 199

*turbinatus* Pfeiffer, 1845—II, 42

*turneri* Pfeiffer, 1860—II, 43

*turritella* d’Orbigny, 1835—III, 199

*turritellatus* Beck, 1838—※III, 200

*turritus* Broderip in Broderip and Sowerby I 1832—III, 200

*umbilicaris* Souleyet, 1842—III, 200

*umbraticus* Reeve, 1850—※III, 77

*umbricatus* Reeve, 1849—III, 201

*undulosus* Martens, 1893†—IV, 44

*unicolor* Sowerby I, 1833—※III, 92; ※III, 98; ※III, 149

*unifasciatus* Sowerby I, 1833—III, 202

*ustulatus* Sowerby I, 1833—※III, 150; III, 202

*varians* Broderip in Broderip and Sowerby I 1832†—IV, 44

*varicosus* Pfeiffer, 1853—III, 203

*variegata* Pfeiffer, 1842—II, 43

*venezuelensis* Pfeiffer, 1856†—IV, 44

*venosus* Reeve, 1848†—IV, 44

*ventricosus* Preston, 1907†—IV, 44

*ventrosus* Reibisch, 1892—III, 203

*veranyi* Pfeiffer, 1848—※I, 38; I, 44

*verrucosus* Pfeiffer, 1855—III, 203

*versicolor* Broderip in Broderip and Sowerby I 1832—※III, 46; III, 204

*veruculum* Morelet, 1860—III, 204

*vesicalis* Pfeiffer, 1853—III, 205

*vespertinus* Pfeiffer, 1858—III, 205

*vexillum* Broderip in Broderip and Sowerby I 1832†—IV, 44

*vexillum* Wood, 1828†—※III, 103; IV, 44

*vicaria* Fulton, 1896—IV, 35

*vicinus* Preston, 1907†—IV, 44

*victor* Pfeiffer, 1854—IV, 36

*vilchezi* Weyrauch, 1960—III, 206

*vincentina* E.A. Smith, 1895—IV, 37

*vincentinus* Pfeiffer, 1846—III, 206

*virginalis* Pfeiffer, 1856—III, 207

*virgultorum* Morelet, 1863—III, 207

*vitrinoides* Reeve, 1848¶—IV, 40

*vittatus* Broderip in Broderip and Sowerby I 1832†—IV, 44

*voithianus* Pfeiffer, 1847—III, 208

*volsus* Fulton, 1907—III, 208

*wintlei* Finch, 1929—III, 209

*wolfi* Reibisch, 1892—III, 209

*woodwardi* Pfeiffer, 1857—III, 210

*xanthostoma* d’Orbigny, 1835—III, 210

*yanamensis* Morelet, 1863—IV, 37

*yatesi* Pfeiffer, 1855—※IV, 36; IV, 38

*yungasensis* d’Orbigny, 1837—III, 211

*zhorquinensis* Angas, 1879—III, 211

*ziczac* da Costa, 1898—III, 212

*ziegleri* Pfeiffer, 1847—※III, 37; ¶III, 212

*ziegleri* Reeve, 1849†—IV, 44

*zilchi* Breure, 1977—I, 45

*zilchi* Weyrauch, 1958—※III, 83

*zoographica* d’Orbigny, 1835—III, 213
